# Alkyne as a Latent Warhead to Covalently Target SARS-CoV-2 Main Protease

**DOI:** 10.1021/acs.jmedchem.3c00810

**Published:** 2023-08-18

**Authors:** Chau Ngo, William Fried, Saba Aliyari, Joshua Feng, Chao Qin, Shilei Zhang, Hanjing Yang, Jean Shanaa, Pinghui Feng, Genhong Cheng, Xiaojiang S. Chen, Chao Zhang

**Affiliations:** † Department of Chemistry and Loker Hydrocarbon Research Institute, University of Southern California, Los Angeles, California 90089, United States; ‡ Molecular and Computational Biology, Department of Biological Sciences, University of Southern California, Los Angeles, California 90089, United States; § Department of Microbiology, Immunology and Molecular Genetics, University of California Los Angeles, Los Angeles, California 90095, United States; ∥ Section of Infection and Immunity, Herman Ostrow School of Dentistry, University of Southern California, Los Angeles, California 90089, United States

## Abstract

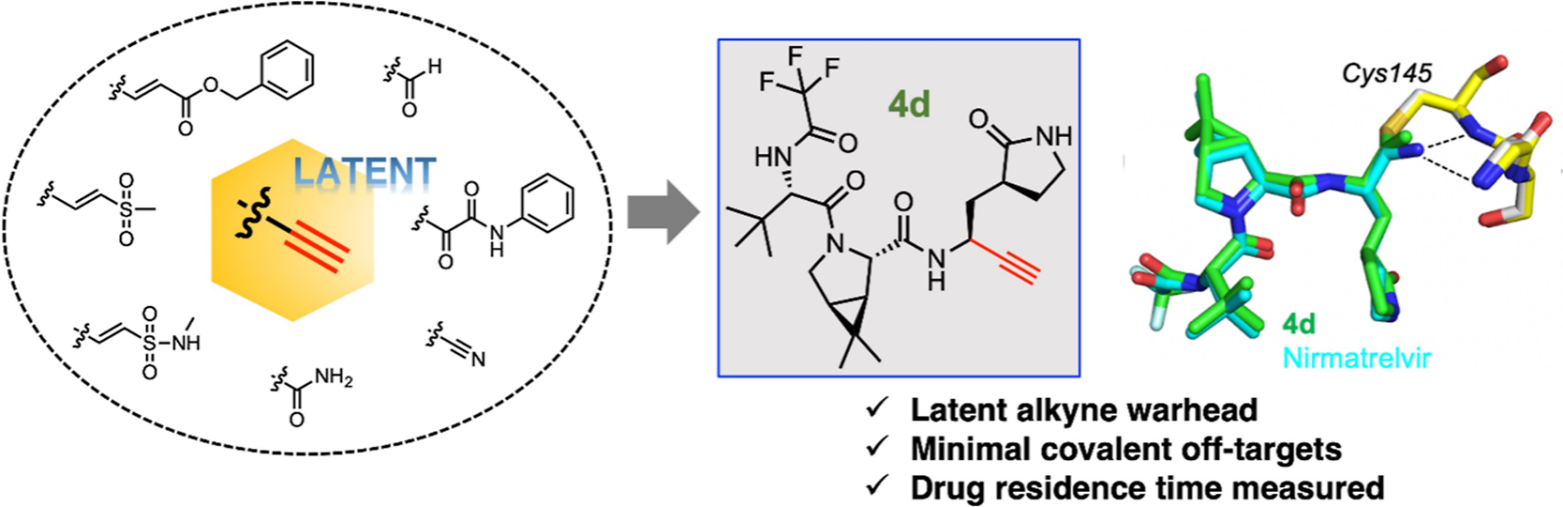

There is an urgent need for improved therapy to better control the ongoing COVID-19 pandemic. The main protease M^pro^ plays a pivotal role in SARS-CoV-2 replications, thereby representing an attractive target for antiviral development. We seek to identify novel electrophilic warheads for efficient, covalent inhibition of M^pro^. By comparing the efficacy of a panel of warheads installed on a common scaffold against M^pro^, we discovered that the terminal alkyne could covalently modify M^pro^ as a latent warhead. Our biochemical and X-ray structural analyses revealed the irreversible formation of the vinyl-sulfide linkage between the alkyne and the catalytic cysteine of M^pro^. Clickable probes based on the alkyne inhibitors were developed to measure target engagement, drug residence time, and off-target effects. The best alkyne-containing inhibitors potently inhibited SARS-CoV-2 infection in cell infection models. Our findings highlight great potentials of alkyne as a latent warhead to target cystine proteases in viruses and beyond.

## Introduction

The coronavirus disease 2019 (COVID-19), caused by the novel severe acute respiratory syndrome coronavirus 2 (SARS-CoV-2), has emerged as a global pandemic since its outbreak in December 2019.^1,2^ As of January 2023, roughly three years after its initial breakout, COVID-19 continues to pose serious threats to human health and public safety, with more than 762-million confirmed cases and 6.8 million deaths worldwide.^3^ Although vaccination continues to remain the most effective therapeutic strategy to protect people against serious illness or death from COVID-19, the emergence and global spread of highly contagious SARS-CoV-2 variants harboring spike mutations have raised concerns about vaccine effectiveness due to the potential of these variants to escape existing SARS-CoV-2 neutralizing antibodies.^4−6^ In addition, individuals with immunocompromised conditions often exhibit significantly low seroconversion rates after vaccination, resulting in poor vaccine protection against COVID-19.^7,8^ The current vaccination approach is also limited in its ability to protect people with historically severe allergic reactions to vaccines by putting this population at an increased risk of life-threatening hypersensitivity adverse events such as anaphylaxis after getting vaccinated, thereby giving rise to hesitation in receiving COVID-19 vaccines and bringing challenges to achieving herd immunity against this infection.^9,10^ Thus, despite the wide availability of vaccines, there is still an urgent need for the development of other broadly protective interventions to halt the devastation of the evolving pandemic. Among therapeutic interventions, drug discovery efforts in developing specific antiviral agents against SARS-CoV-2 proffer a powerful addition to the host defense mechanisms for combating COVID-19 and eradicating future pandemics.

SARS-CoV-2 is an enveloped positive-sense single-stranded RNA virus of approximately 30 kb in size, belonging to the genus *Betacoronavirus* of the family Coronaviridae.^11,12^ Upon host cell entry, the SARS-CoV-2 genomic RNA is translated by the host’s machinery into two large overlapping polyproteins (pp1a and pp1ab), four structural proteins, and other accessory proteins.^11,13^ One of the key steps in the viral replication cycle involves the proteolytic cleavage of the polyproteins pp1a and pp1ab into 16 highly conserved non-structural proteins (Nsps) for the subsequent formation of the replication–transcription complex.^13,14^ Together with the papain-like protease, the main protease (M^pro^) of SARS-CoV-2, also known as 3-chymotrypsin-like protease (3CLpro) or Nsp5, performs critical proteolytic processing at distinct cleavage sites to yield the 11 mature Nsps required for viral replication.^14,15^ SARS-CoV-2 M^pro^ therefore represents one of the most attractive therapeutic targets for the development of antiviral therapy for treating COVID-19.

SARS-CoV-2 M^pro^ (abbreviated as M^pro^ in the remainder of the paper) is a cysteine (Cys) protease that comprises three domains that are highly conserved in all known coronaviruses and contains an active site for enzymatic proteolytic function.^16,17^ M^pro^ preferentially cleaves substrates with the consensus sequence (**P2:Leu**/Met/Phe/Val)–**P1:Gln**↓(**P1**^**’**^**:Ser/Ala**/Gly) (↓ indicates the cleavage site).^18^ The absolute requirement of the Gln residue at the P1 position is notably advantageous, as no known human proteases have such unique substrate selectivity, thereby promising high safety profiles for specific antiviral agents against M^pro^.^19,20^ More importantly, the presence of a catalytic Cys^145^ residue from the catalytic Cys^145^–His^41^ dyad in the active site renders M^pro^ susceptible to targeted covalent inhibition by small-molecule inhibitors featuring reactive electrophilic groups.^19,21^

Since the onset of the COVID-19 pandemic, a large number of covalent inhibitors containing diverse electrophilic warheads, including α-ketoamides, aldehydes, α,β-unsaturated ketones, hydroxymethylketones, vinyl sulfones, and nitriles, have been reported to covalently inhibit M^pro^.^19,21−25^ Paxlovid (Pfizer), consisting of primarily nirmatrelvir along with ritonavir, has been granted emergency use for the treatment of COVID-19 by the U.S. Food and Drug Administration (FDA) as the first antiviral acting via covalent inhibition of SARS-CoV-2 M^pro^.^25^ Treatment with Paxlovid early in COVID-19 illness has shown an approximately 90% reduction in the risk of progression to severe disease and been associated with a significant decrease in COVID-19-related hospitalizations or deaths.^26^ Paxlovid owes its potent antiviral activity to the ability of its active ingredient, nirmatrelvir, to reversibly and covalently target the catalytic Cys^145^ residue via a nitrile warhead.^25^ Despite the proven effectiveness of nirmatrelvir in disarming SARS-CoV-2, Paxlovid is not recommended for individuals with severe renal or hepatic impairment and contraindicated with CYP3A-dependent medications.^27^ Furthermore, mutations in M^pro^ have emerged and conferred resistance against nirmatrelvir in COVID-19 patients.^28^ These gaps highlight the urgent need to develop novel inhibitors against this viral protease as second-generation anti-COVID therapies.

Electrophilic warheads usually have intrinsic reactivity toward certain nucleophilic groups. For example, the acrylamide that is commonly employed to target the cysteine thiol group can react with thiols in a bi-molecular reaction, which is responsible for nonspecific binding to undesired proteins (off targets), especially abundant cellular proteins.^29,30^ In contrast, latent warheads do not have intrinsic reactivity but can react with certain functional groups only upon activation at the active site of certain enzymes. Eflornithine, a drug that is used to treat sleeping sickness and excessive hair growth, cannot react with thiols or cysteine on its own.^31^ However, when bound to ornithine decarboxylase, eflornithine is activated by the catalytic environment to produce a derivative that covalently modifies a cysteine in the enzyme.^32^ Latent warheads, with their lack of intrinsic chemical reactivity, are often considered advantageous over regular warheads because they tend to be more specific for intended enzyme targets and have much fewer covalent off-targets in cells and organisms.^30,33,34^

We set out to discover M^pro^ inhibitors harboring novel electrophilic warheads that may offer new opportunities to treat COVID-19. We began this campaign by installing a panel of cysteine-targeting warheads on a common peptidomimetic scaffold and comparing their inhibition against M^pro^. A terminal alkyne, acting as a latent warhead, was found to afford strong inhibition of M^pro^ in the panel of resulting peptidomimetic derivatives. Installation of the terminal alkyne on more elaborate scaffolds led to the identification of inhibitors with comparable potency of biochemical inhibition against M^pro^. The irreversible inhibition of M^pro^ by these alkyne-containing inhibitors was verified by both biochemical and X-ray structural characterizations and was exploited to generate “clickable” probes for measuring targeting engagement in vitro and in situ. Finally, our alkyne-containing inhibitors exhibited anti-viral activity in cellular models of COVID-19 infection.

## Results and Discussion

### Design, Synthesis, and Biochemical Characterization of M^pro^ Inhibitors

**Figure 1:**
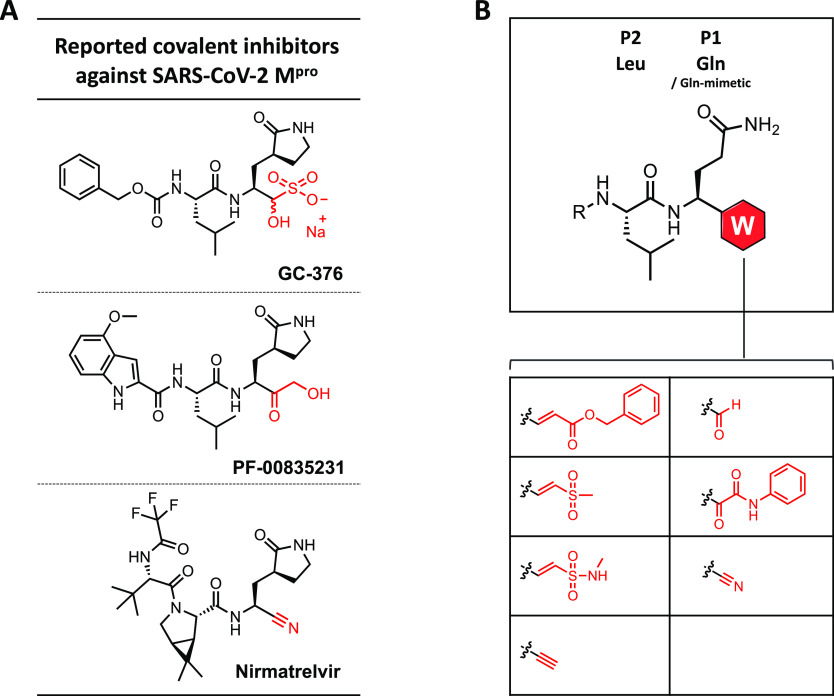
Clinical-stage inhibitors of M^pro^ and a panel of initial peptidomimetic inhibitors. (A) Covalent inhibitors of M^pro^ that are clinically used or underwent clinical evaluation. (B) General design of a panel of peptidomimetic inhibitors featuring various electrophilic warheads, including α,β-unsaturated ester, vinylsulfone, vinylsulfonamide, terminal alkyne, aldehyde, ketoamide, and nitrile.

Aiming to identify the most attractive warheads in an unbiased manner, we first chose to install a panel of electrophilic warheads on a common dipeptide scaffold. Given that Gln and Leu were found as the highly preferred residues at the P1 and P2 positions, respectively, in peptide substrates of M^pro^, this panel of peptidomimetics was designed to consist of three components: a benzyloxycarbonyl (Cbz) cap at the N terminus, a Leu residue, and a Gln analogue linked to a Cys-reactive electrophile at the C terminus. The electrophilic warheads that we selected include α,β-unsaturated esters, vinylsulfones, vinylsulfonamides, terminal alkynes, aldehydes, ketoamides, and nitriles (Figure 1B).^21,22,24,25,35,36^ Five warheads within the panel were previously employed to covalently target M^pro^, whereas vinylsulfonamides and terminal alkynes were not until during the preparation of our manuscript.^37^

The general synthesis of these peptidomimetics included the generation of a Weinreb amide from Fmoc-Gln(Trt)-OH, followed by the installation of the second required Leu residue from N-carbobenzyloxy-l-leucine to finally yield a N-protected dipeptide intermediate (Schemes S1–S7). Selective reduction of this Weinreb amide intermediate was then carried out in the presence of lithium aluminum hydride to form an important aldehyde precursor, which was used for synthesizing six compounds (**1a–f**) within the series (Table 1). Covalent warheads featured in these compounds include α,β-unsaturated ester, vinylsulfone, vinylsulfonamide, terminal alkyne, aldehyde, and ketoamide. The final nitrile-containing compound **1g** was synthesized in a similar route but involved key conversion of the primary amide to the target nitrile group.

**Table 1:**
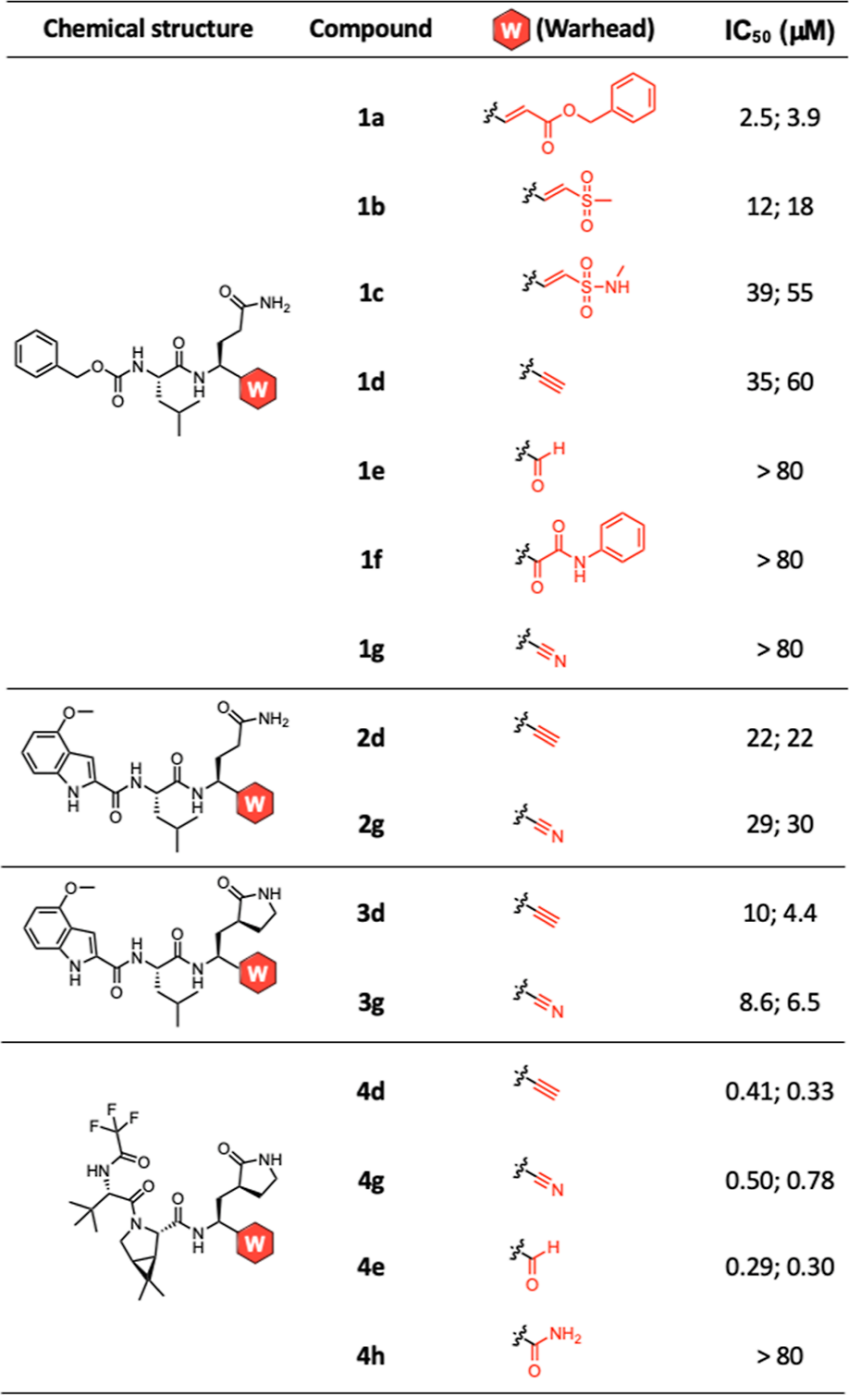
Biochemical IC_50_ Values (μM) of Covalent Inhibitors against M^pro^ via a FRET-Based Enzymatic Assay^a^

a IC_50_ values were determined in vitro in the presence of the FRET substrate (20 μM) after 15-min treatment of M^pro^ (0.5 μM) with each compound at various concentrations. Measurements of the inhibitory activities of the compounds were performed in duplicate. IC_50_ values in duplicates were shown except for those that caused little inhibition at up to 80 μM.

Having synthesized the panel of Cbz-capped peptidomimetics, we screened them for inhibition against M^pro^ in a fluorescence resonance energy transfer (FRET)-based cleavage assay in vitro.^36,38^ M^pro^ was subcloned and expressed in *E. coli* before affinity purification via a hexa-His tag (Figure S1). Dose response studies revealed the half-maximal inhibitory concentration (IC_50_) values of compounds **1a–1g**. Out of the 7 compounds, **1a**, which contains an acrylate warhead, exhibited the strongest inhibitory activity against M^pro^ with an IC_50_ of 5.0 μM (average of duplicate, Table 1). **1b** and **1c**, which contain a warhead of vinyl sulfone and vinyl sulfonamide, have IC_50_ values of 18 and 47 μM, respectively. It was previously reported that vinyl sulfones reacted with thiols more rapidly than the analogous acrylates due to the stronger electrophilicity of the former group.^39,40^ However, we found that the acrylate-containing **1a** inhibited M^pro^ more potently than the corresponding vinyl sulfone analogue. This discrepancy may be due to the better fit of the ester, including the benzyl group, than the sulfone group in the S1′ pocket.^21^ The potency difference between **1b** and **1c** at inhibiting M^pro^ is consistent with the thiol reactivity difference between vinyl sulfones and vinyl sulfonamides.

Compounds **1e**, **1f,** and **1g**, which all contain reversible covalent warheads, failed to cause significant inhibition against M^pro^ up to 80 μM. These results suggest that the efficacy of the reversible warheads requires a scaffold of moderate or high noncovalent binding affinity to M^pro^. The Cbz-Leu-Gln scaffold apparently cannot deliver sufficient levels of noncovalent binding to M^pro^ to manifest the effects of these reversible covalent warheads. Compound **1d**, which contains a terminal alkyne, inhibited M^pro^ with an IC_50_ of 61 μM. A terminal alkyne has been commonly used as a “clickable” tag because it is largely considered chemically inert toward various compounds in cells.^41,42^ We chose to include the terminal alkyne in the panel of Cbz-Leu-Gln derivatives because prior studies demonstrated that it served as a latent warhead to covalently target cathepsin K protein and deubiquitinase.^33,35,43^ In these applications, the terminal alkyne covalently modified the catalytic cysteine of the targeted cysteine protease by forming a vinyl thioether linkage.^33,35,43^ Unlike nitrile, which acts as a reversible warhead towards cysteine, the thiol–alkyne addition is irreversible, yielding a permanent covalent ligand–protein adduct.^44^ The advantages of irreversible covalency, the lack of indiscriminate thiol reactivity, and the common applications in targeting diverse cysteine proteases highlight our selection of alkyne as an ideal warhead candidate in the development of covalent inhibitors against M^pro^. Attracted by the unique advantages of a latent irreversible warhead, we decided to install terminal alkyne on more sophisticated scaffolds in the hope that more potent inhibition of M^pro^ could be attained.

We next replaced the Cbz moiety with a 4-methoxy indole cap, which was adopted from a clinical candidate PF-00835231 (Figure 1A), in the Cbz-Leu-Gln derivatives.^23^ Previous structural analysis revealed extensive van der Waals interactions of the indole group in PF-00835231 with residues in the S3 subsite.^23^ Both terminal alkyne and its isostere nitrile were installed on the In-Leu-Gln scaffold, yielding **2d** and **2g**, respectively (Schemes S8 and S9).^33^ Both **2d** and **2g** were found to be a few times more potent at inhibiting M^pro^ than **1d** and **1g**, confirming that the indole cap afforded improved binding to the protease than Cbz. Importantly, **2d** is slightly more potent than **2g**, indicating that the terminal alkyne was comparable to or slightly better than the nitrile as the warhead targeting M^pro^. The use of a lactam analogue of Gln, first featured in the discovery of rupintrivir (Pfizer),^45^ has been found to enhance binding to viral proteases compared to the more flexible Gln residue.^46,47^ We thus substituted the Gln with its lactam analogue (Qla) surrogate in the additional compounds. Installation of alkyne and nitrile on a modified scaffold of In-Leu-Qla led to compounds **3d** and **3g** (Scheme S10), which showed comparable potency (both ∼7 μM) at inhibiting M^pro^. The improved potency of **3d** over **2d** and over **1d** highlights the importance of the scaffold as the foundation of covalent inhibition.

Our further medicinal chemistry efforts involved replacing the nitrile warhead with alkyne in nirmatrelvir, the clinically used M^pro^ inhibitor that has undergone extensive chemical optimization (Figure 1A).^25^ In addition to the lactam analogue of Gln, nirmatrelvir also contains a cyclic variant (6,6-di-methyl-3-azabicyclo[3.1.0]hexane) of Leu and a trifluoroacetyl capping group at the N terminus.^25^ The benefits of this cyclized form of Leu include the removal of a hydrogen bond donor to increase cell permeability and improved fit into M^pro^ S3 pocket because of preorganization or entropic gain.^25^ Compound **4d** was synthesized following routes that were modified based on the reported synthesis of nirmatrelvir (Scheme S11).^48^ We also synthesized the nitrile-containing nirmatrelvir and the aldehyde-containing **4e** for comparison among the warheads. When tested in M^pro^ FRET assays, **4d** was found to have an IC_50_ of 0.30 μM, roughly two times lower than that of nirmatrelvir (0.56 μM), which was determined under the same conditions. The potency of **4d** is similar to the aldehyde-bearing analogue **4e** with an IC_50_ of 0.30 μM, indicating that the latent alkyne warhead appears to work as well as aldehyde, a warhead with high intrinsic reactivity. To evaluate the non-covalent binding afforded by the nirmatrelvir scaffold, we also synthesized an additional nirmatrelvir analogue that contains a primary amide in place of the warhead. Interestingly, this control compound caused no significant inhibition against M^pro^ in the FRET assay. These results highlight the effectiveness of the latent alkyne warhead at covalently inhibiting M^pro^.

### Irreversible Inhibition of M^pro^ by the Alkyne Inhibitors

**Figure 2:**
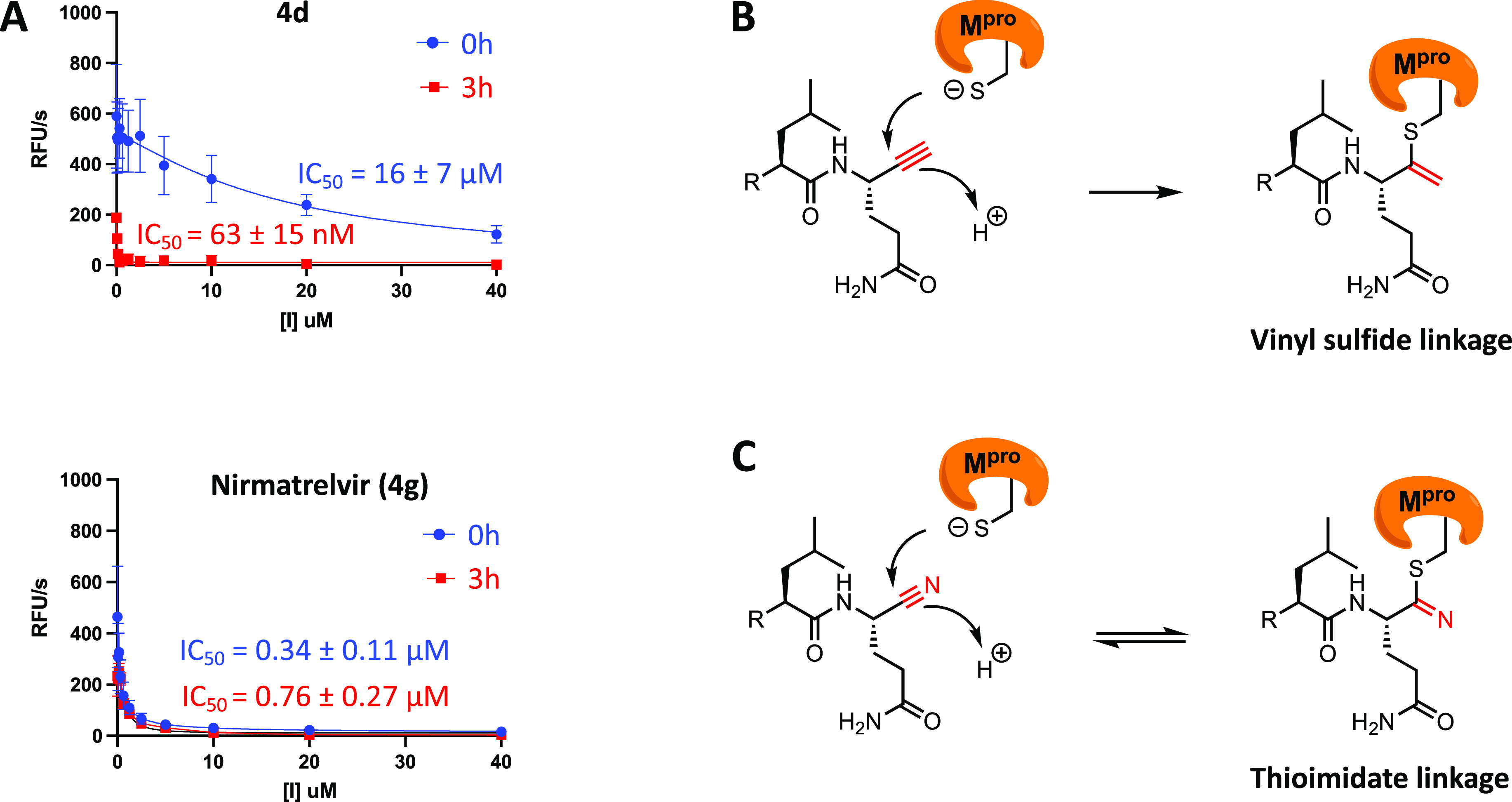
Time-dependent inhibition of M^pro^ by alkyne **4d** and nirmatrelvir (**4g**). (A) M^pro^ (0.5 μM) was incubated with increasing concentrations (up to 40 μM) of **4d** or nirmatrelvir (**4g**) in the reaction buffer at 30 °C for 0 or 3 h. The reactions were then initiated by the addition of the FRET substrate (20 μM) followed by continuous measurements of fluorescence for 1 h. IC_50_ values were determined in duplicates and shown as the mean ± SD. (B) Proposed irreversible thiol–alkyne addition of alkyne-containing inhibitors with M^pro^ to form a vinyl sulfide linkage. (C) Known reversible reaction of nitrile inhibitors, such as nirmatrelvir, with M^pro^ to form a thioimidate linkage.

Thus, unlike the nitrile warhead in nirmatrelvir, which yields a reversible covalent thioimidate adduct (Figure 2C),^25^ our alkyne inhibitors are expected to undergo a two-step irreversible binding involving the initial reversible association with M^pro^ followed by an irreversible modification of the protease at the catalytic Cys^145^ residue.^49^ Specifically, upon reversibly binding at the catalytic site that enables precise positioning toward Cys^145^, the latent alkyne would be no longer bioinert but activated by the catalytic environment toward a hydrothiolation reaction, forming an irreversible vinyl thioether linkage and permanently inactivating M^pro^ (Figure 2B).

To determine the reversibility of their inhibition, we subjected both **4d** and nirmatrelvir (**4g**) to the same FRET-based cleavage assay and examined their time-dependent inhibition of M^pro^ (Figure 2A). Dose response inhibition of recombinant M^pro^ by **4d** or nirmatrelvir using the FRET assay was performed either without preincubation or with a 3 h preincubation. The IC_50_ of nirmatrelvir did not change significantly after the 3 h preincubation, consistent with the reversible inhibition via the nitrile warhead. In contrast, the 3 h preincubation of M^pro^ with compound **4d** lead to a 254-time increase of IC_50_, supporting the inhibition acting in an irreversible process.^50^ Furthermore, the dramatic change of IC_50_ of **4d** against M^pro^ upon alteration of the preincubation time suggests that the rate of its inactivation (*k*_inact_) is relatively low, which is consistent with the nature of a latent warhead.

Taking advantage of the irreversibility of alkynes as latent electrophiles,^33,43^ we went on to identify the amino acid within M^pro^ that was covalently modified by **4d** using liquid chromatography tandem mass spectrometry (LC–MS/MS). Recombinant M^pro^ was incubated with excess **4d** for 4 h, followed by digestion with trypsin and Glu-C prior to LC–MS/MS analysis. LC–MS/MS data revealed that the catalytic Cys (Cys^145^) was modified by **4d,** as predicted (Figure S3). No other residues were found to be modified in the mass spec analysis, highlighting the exquisite selectivity of our alkyne inhibitor for the catalytic Cys residue.

### In Vitro and In Situ Labeling of M^pro^ by a Clickable Analogue of **4d**

To further confirm the irreversible binding of **4d** to M^pro^ and allow for measurement of its target engagement, we synthesized **Alk-4d**, an analogue of **4d**, by replacing the *tert*-butyl group at the P3 position in **4d** with a propargyl group (Figure S4A). This replacement was predicted to cause minimal perturbation to **4d**′s binding to M^pro^ since the *tert*-butyl group was found to protrude toward the solvent in the solved crystal structures of M^pro^-nirmatrelvir complexes. **Alk-4d** was first evaluated for its ability to label recombinant M^pro^ in an in-gel fluorescence experiment, which involved incubation of the probe with the protein, click conjugation to TAMRA azide, SDS-PAGE resolution, and fluorescence imaging of the gel (Figure S4B). With 1 h of pre-treatment, **Alk-4d** efficiently labeled M^pro^ at concentrations above 1 μM, reaching saturation at 3 μM. We next turned to competitive labeling experiments to confirm the target engagement of **4d**. Pre-treating recombinant M^pro^ with **4d** dramatically reduced the labeling of M^pro^ by **Alk-4d** due to the prior occupancy of M^pro^ by **4d**. Nirmatrelvir also efficiently blocked the labeling of M^pro^ by **Alk-4d** in co-treatment experiments.

We net examined the labeling of M^pro^ by **Alk-4d** in live cells. By treating HEK293T cells that were transiently transfected with M^pro^ with **Alk-4d**, we observed dose-dependent labeling of M^pro^ by the probe (Figure S4C). The analogous experiment with the C145A mutant of M^pro^ yielded no labeling, supporting Cys^145^ being the residue being modified by **Alk-4d** (Figure S4D). Interestingly, treatment of parental HEK293T cells with **Alk-4d** with high concentrations of **Alk-4d** did not label any proteins, as revealed by the in-gel fluorescence experiment, indicating that there were no prominent covalent off-targets of this probe in human cells, including HEK293T and HeLa cells (Figure S5A). To answer the question of whether our alkyne inhibitors might hit host cysteine proteases in view of the precedence of alkyne-containing inhibitors of cathepsin K,^33^ we tested **4d** on three human cysteine proteases—cathepsin B, cathepsin K, and cathepsin L. Despite a 20 min preincubation in the cathepsin enzymatic assays, **4d** at up to 40 μM caused little inhibition against the cathepsin proteases (Figure S6). This is consistent with the distinct substrate recognition motif of SARS-CoV-2 M^pro^ from those of human cysteine proteases.^19^ Taken together, the proteomic labeling and biochemical data support the high selectivity of our alkyne inhibitors for SARS-CoV-2 M^pro^.

While **Alk-4d** succeeded in labeling M^pro^ in situ, we noticed that a relatively high concentration (20 or 40 μM) of **Alk-4d** was often needed to produce consistent labeling. We attribute the poor in situ labeling to the slow kinetics of M^pro^ modification by **Alk-4d**, which motivated us to tune up the reactivity of the alkyne group through chemical modifications.

### Tuning of Alkyne Reactivity via Substitution with an Electron-Withdrawing Group

**Figure 3:**
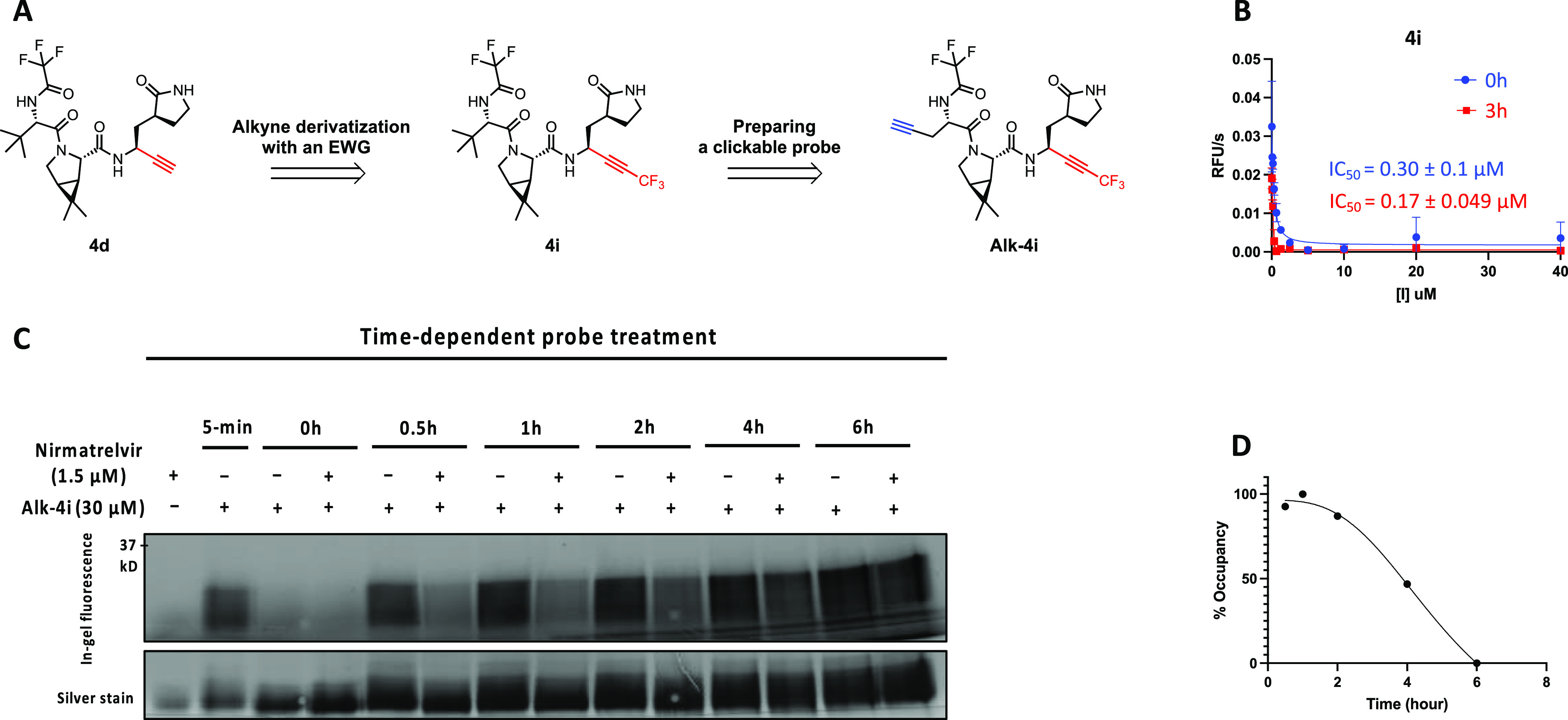
Tuning up alkyne reactivity through a chemical modification. (A) Alkyne substitution with an electron-withdrawing group (EWG), trifluoromethyl (CF3), to tune its warhead reactivity. A clickable probe, **Alk-4i**, was derived from **4i** by replacing the solvent-exposed *tert*-butyl group with an alkyne reporter tag. (B) **4i** inhibited M^pro^ and dramatically increased the rate of M^pro^ inactivation in the FRET-based enzymatic assay. (C) Measurements of the residence time of nirmatrelvir **(4g**) with M^pro^ by using **Alk-4i** in a competitive labeling experiment. Treatment of M^pro^ with a saturating concentration of nirmatrelvir (1.5 μM) for 30 min, followed by treatment with an excessive concentration (30 μM) of **Alk-4i**, revealed the dissociation kinetics of nirmatrelvir from M^pro^. (D) Change of M^pro^ occupancy by nirmatrelvir over time. The data in (C) were quantified and normalized to generate the temporal curve.

The inhibitors and probes that harbor a terminal alkyne were found to have relatively slow kinetics at covalent inactivation of M^pro^, as described above. We reasoned that attachment of an electron-withdrawing group (EWD) at the terminal carbon would increase the reactivity of the alkyne toward the catalytic cysteine in M^pro^. We chose to install a trifluoromethyl (CF_3_) group at the alkyne to yield a trifluoromethylated analogue **4i** (Figure 3A), inspired by the successful introduction of CF_3_ on a terminal alkyne to better covalently target deubiquitinases.^44^

**4i** was synthesized following a similar route to that of **4d** (Scheme S11). When subjected to the FRET-based cleavage assay (Figure 3B), **4i** showed an IC_50_ of 0.30 μM without pre-incubation with the enzyme. Adding a 3 h preincubation prior to the assay only reduced **4i**’s IC_50_ slightly to 0.17 μM. These results are consistent with the prediction that the introduction of a CF_3_ group dramatically increased the rate of M^pro^ inactivation.

We next examined the potency of **4i**′s engagement to M^pro^ in vitro by performing a competitive labeling experiment. A dose response study revealed that 1 μM of **4i** was sufficient to abolish the labeling of M^pro^ by 10 μM of **Alk-4d** (Figure S7A). Similar dose–response studies were performed for **4d** and nirmatrelvir. The data indicate that **4i** has comparable potency at engaging M^pro^ to nirmatrelvir and that **4i** is approximately 10-fold more potent at engaging M^pro^ than **4d** in vitro. Kinetic characterizations allowed us to determine the *k*_inact_/*K*_I_ values of 5.3 × 10^7^ (M^–1^ s^–1^) for **4d** and 4.9 × 10^8^ (M^–1^ s^–1^) for **4i**, which differ by ∼10 folds. Taken together, these results suggest that the introduction of a CF3 group at alkynes substantially increased the binding affinity of M^pro^, apparently through enhancing the rate of enzyme inactivation.

### Measurement of Nirmatrelvir’s M^pro^ Residence Time

We next derivatized the more potent and faster-acting alkyne inhibitor **4i** with a propargyl group at P3 to yield a clickable probe **Alk-4i** (Scheme S13, Figure 3A). In-gel fluorescence analysis of the probe-treated M^pro^ showed that **Alk-4i** covalently targeted the main protease in a concentration-dependent manner and exhibited intense labeling at the lowest concentration tested, 300 nM. Moreover, **Alk-4i** labeled M^pro^ near saturation after a mere 5 min incubation, demonstrating rapid labeling kinetics as predicted (Figure S7B).

With a more potent and faster-acting clickable probe **4i** available, we used it to directly measure the residence time of nirmatrelvir with M^pro^. A pulse-chase like labeling experiment was performed (Figure 3C), involving an initial 30 min treatment of recombinant M^pro^ with nirmatrelvir (1.5 μM) for the drug to occupy the most active sites, followed by adding an excessive concentration (30 μM) of **Alk-4i** to label sites vacated after nirmatrelvir dissociation at different time points. Without nirmatrelvir pretreatment, the labeling of M^pro^ by **Alk-4i** did not change much during 6 h as expected. With nirmatrelvir pretreatment, the labeling of M^pro^ by **Alk-4i** increased over 6 h, consistent with the slow dissociation of nirmatrelvir from M^pro^. From the curve (Figure 3D), an approximate half-life (*t*_1/2_) of 3.5 h was derived, which is the first reported residence time for M^pro^-nirmatrelvir complex to our knowledge.

To investigate whether the derivatization of alkynes with an electron-withdrawing trifluoromethyl group would leave **Alk-4i** with greater non-specific proteomic labeling because of the boosted reactivity of the alkyne group, we turn to in-gel fluorescence experiments. Parental HEK293T cells were treated with either **Alk-4d** or **Alk-4i** at various concentrations for 5 h, harvested, and processed for in-gel fluorescence analysis (Figure S5B). While **Alk-4d** at all tested concentrations caused little proteome labeling, 10 μM of **Alk-4i** induced significant labeling of the HEK293T proteome. These data suggest that boosting the reactivity of alkynes with a CF_3_ group resulted in non-specific proteome labeling, which was not significant at low micromolar concentrations.

### Structure Basis of Covalent Inhibition of M^pro^ by Alkyne-Containing Compounds

To elucidate the molecular basis for the covalent inhibition of M^pro^ by alkyne-containing inhibitors, we solved the X-ray crystal structures of the protease in complex with **3d** and **4d**. Recombinant M^pro^ was purified using a RESOURCE Q anion exchange column after affinity purification using the Ni-NTA column. The purified M^pro^ was then co-crystalized with either **3d** or **4d**, with the resulting crystals diffracting at synchrotron to resolutions of 1.9 and 2.0 Å, respectively. Molecular replacement using M^pro^ with nirmatrelvir (PDB: 7RFS) as the search model was then performed to solve the structures.

**Figure 4:**
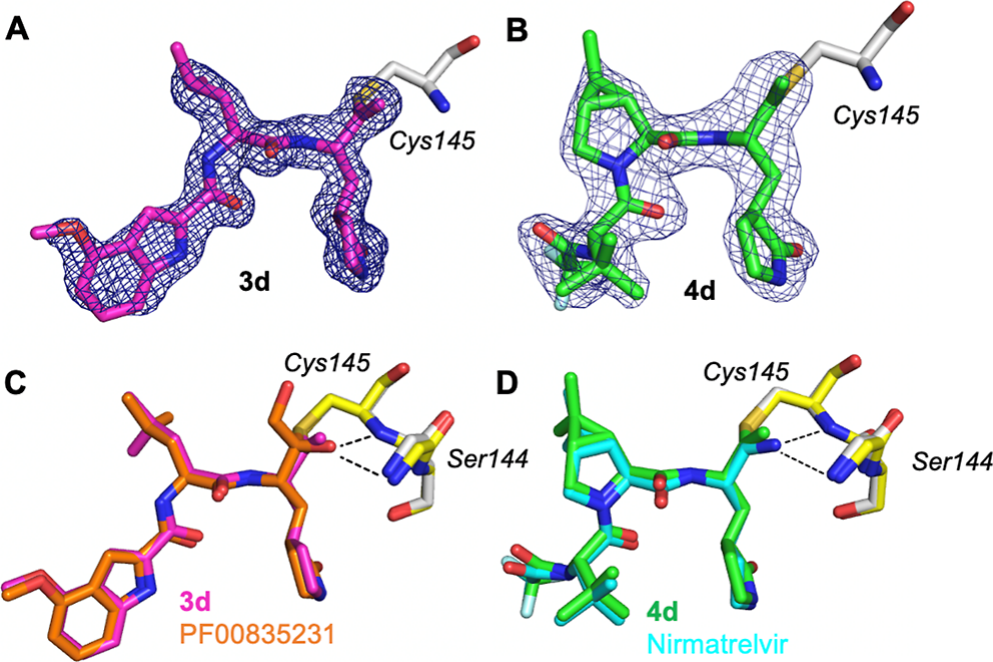
Crystal structures of M^pro^ in complex with two alkyne-containing inhibitors. (A) Representative OMIT electron density map (mF_o_ – DF_c_) contoured to 3σ for **3d** in complex with M^pro^ (PDB: 8FY7). (B) Representative OMIT electron density map (mF_o_ – DF_c_) contoured to 3σ for **4d** in complex with M^pro^ (PDB: 8FY6). (C) Structural overlay of M^pro^-**3d** complex (PDB: 8FY7) with the reported M^pro^-PF00835231 complex (orange). (D) Structural overlay of M^pro^-**4d** complex (PDB: 8FY6) with the reported M^pro^-nirmatrelvir complex (cyan).

The complex structures of M^pro^-**3d** and M^pro^-**4d** both confirmed the presence of a covalent bond between the sulfur atom of Cys^145^ and the internal carbon of alkyne (Figure 4). The indole ring N-terminal capping group within **3d** forms extensive contacts with several hydrophobic residues in M^pro^, while the leucine residue and the lactam moiety occupy the S2 and S1 pockets of the protease, as predicted. The thiol–alkene adduct resulting from the addition of thiol to alkyne can be clearly visualized based on the electron density. A structural overlay of M^pro^ in complex with **3d** or PF00835231 (PDB: 6XHM), a close analogue containing the same indole-peptidic scaffold, revealed the inhibitors are largely superimposable with the exception of the thiol-modified warheads (thioalkene vs hemithiolketal). The structure of M^pro^-**4d** is very similar to the previously reported M^pro^-nirmatrelvir structures. The RMSD between **4d** and nirmatrelvir is merely 0.25 Å, consistent with the isosteric nature of the two inhibitors. Despite their different chemical structures, the thiol–alkene, resulting from the reaction of **4d** with M^pro^, and the imidothiol ester, resulting from the reaction between nirmatrelvir and M^pro^, are essentially superimposable in an overlay of our M^pro^-**4d** structure and a M^pro^-nirmatrelvir structure (PDB: 7RFS). These observations support the notion that a similar mechanism for thiol addition is shared between a nitrile and an alkyne.

### Evaluation of the Anti-COVID Activity of Alkyne-Containing Compounds

We next evaluated the antiviral activity of our most potent M^pro^ inhibitors in a SARS-CoV-2 in vitro infection model. Specifically, we infected Hela cells constitutively expressing ACE2 (HeLa-ACE2)^51^ with SARS-CoV-2 at a multiplicity of infection (MOI) of 0.01 for 1 h to allow for cell entry of SARS-CoV-2. The infected cells were then treated with either **4d**, **4i**, or nirmatrelvir for 24 h, followed by quantification of the viral RNA transcripts in the cell lysates.

**Figure 5:**
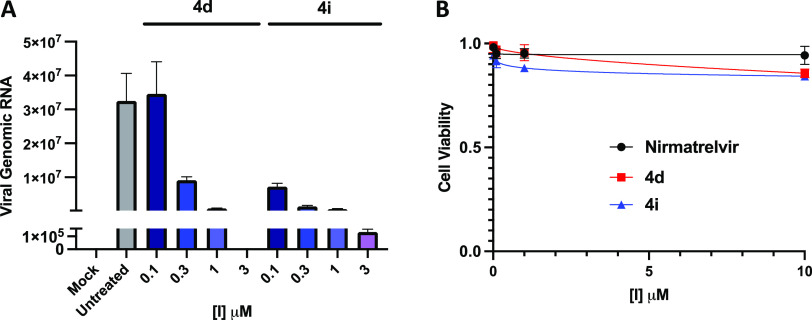
Alkyne inhibitors exhibit strong anti-COVID activity and minimal cell toxicity. (A) Anti-SARS-CoV-2 efficacy of the compounds in HeLa-ACE2 cells. (B) Cytotoxicity of the compounds in HeLa-ACE2 cells.

Both **4d** and **4i** showed strong, dose-dependent antiviral activity, as predicted by their potent inhibition against recombinant M^pro^ in vitro (Figure 5A). At 3 μM, **4d** achieved nearly complete inhibition of SARS-CoV-2 infection. **4i** at sub-micromolar concentrations afforded significant anti-COVID activity, showing apparent higher potency than **4d** under the same conditions. This is consistent with the activating effects on the alkyne group from the trifluoromethyl substituent.

Finally, we determined the cytotoxicity of nirmatrelvir, **4d**, and **4i** by treating non-infected Hela-ACE2 cells with these compounds for 20 h, followed by the collection and analysis of cell viability data (Figure 5B). Similar to nirmatrelvir, both of the alkyne-containing inhibitors, **4d** and **4i**, showed no notable cytotoxicity up to the highest concentration tested, 10 μM. Taken together, these data suggest that a latent warhead of terminal alkyne has moderate anti-COVID activity and little toxicity at up to 10 μM to cells.

## Conclusions

We sought novel electrophilic warheads that can be used to covalently target SARS-CoV-2 M^pro^, a crucial component of the virus’s replication process. By synthesizing and screening a panel of warheads, we found that the terminal alkyne could serve as a latent warhead to covalently modify M^pro^. Biochemical and X-ray structural analyses support the notion that the alkyne forms an irreversible vinyl-sulfide linkage with the catalytic cysteine of M^pro^. The best alkyne-containing inhibitors effectively prevented SARS-CoV-2 infection in cell models, indicating the potential of using alkyne as a latent warhead to target cystine proteases in viruses and beyond. The major advantage of latent warheads is that they have little intrinsic chemical reactivity and are only activated for covalent inhibition in the active site of appropriate enzymes.^33^ A previous study reported that while compounds with a nitrile warhead formed covalent adducts with cysteine, alkyne-bearing counterparts yielded 0 to <1% of adducts after incubation with cysteine.^33^ As a result, compounds containing latent warheads tend to have fewer covalent off-targets than their counterparts harboring regular warheads. We found that a terminal alkyne was an efficient latent warhead for targeting SARS-CoV-2 M^pro^, reflected by the observed potent biochemical inhibition, especially with prolonged incubation. However, the rates of inactivation of M^pro^ by inhibitors containing a latent alkyne warhead, **4d**, are not high, with a preincubation of tens of min being required to attain nanomolar inhibition in vitro. We suspect that the relatively slow inactivation of M^pro^ disfavors **4d**-like compounds in the tight race against SARS-CoV-2 infection and replication, which occurs within minutes, and underlies their moderate potency against SARS-CoV-2 infection in cell culture models. We succeeded in increasing the rate of M^pro^ inactivation through derivatization of the alkyne warhead with the electron-withdrawing group CF_3_. Future studies will be required to determine if a terminal or appropriately substituted alkyne can serve as a latent warhead for targeted covalent inhibition in therapeutic development.

## Experimental Section

### Synthetic Procedures

All of the reagents and solvents were obtained via commercial sources and used without further purification, unless otherwise stated. All anhydrous reactions were carried out under a nitrogen atmosphere. Reactions were monitored by thin-layer chromatography (TLC) on glass TLC plates with silica gel coated with fluorescent indicator F254. UV light and TLC stains, including ninhydrin and 2,4-dinitrophenylhydrazine, were used as visualizing agents. Individual intermediates and final compounds were purified by flash column chromatography using an automated Teledyne CombiFlash system (RF + UV–vis). All ^1^H and ^13^C nuclear magnetic resonance (NMR) spectra were obtained on a Varian Mercury 400, 400 MR, or Varian VNMRS-600 spectrometer at ambient temperature. All target compounds were found to be ≥95% pure based on analytical high-performance liquid chromatography analysis. The purities were determined on a Shimadzu LC-20AP system equipped with an SPD-M21A PDA detector set to λ = 254 nm and a Phenomenex C18 (250 × 3.9 mm) column. The analyses were carried out using acetonitrile with 0.1% trifluoroacetic acid (v/v) in the isocratic mode at a flow rate of 1.8 mL/ min for 14 min. The injection peak at ∼2 min was excluded in the quantification of all compounds. The area % of the major peak is ≥95% with respect to the sum of the area % of other detected peaks.

### Protease Inhibition Assays

#### M^pro^

The expression and purification of recombinant M^pro^ were performed as previously described with minor modifications to the protocol.^36^ Briefly, after expression in *E. coli*, M^pro^ was purified using polyHis-Ni affinity chromatography, cleaved with PreScission protease, and then purified by using a ResQ column on an AKTA Pure FPLC system. Purified M^pro^ was concentrated to about 1 mg/mL and stored in a 20% glycerol solution at −80 °C for later use in biochemical studies.

A fluorescent peptide substrate of the sequence Dabcyl-KTSAVLQSGFRKM-E(Edans)-NH_2_ and a reaction buffer composed of 20 mM Tris-HCl, 100 mM NaCl, 1 mM EDTA, 1 mM TCEP, pH 7.3 were used in the enzyme assay.^36^ In the FRET-based cleavage assay, M^pro^ diluted in the reaction buffer was pre-incubated with compounds at various concentrations at 30 °C for a period (0 min, 15 min, or 3 h) before 20 μM FRET substrate was added to initiate the enzymatic reaction. The fluorescence signal was monitored using a Cytation 5 imaging reader (Thermo Fisher Scientific) or a Spectra Max^R^ iD5 (Molecular Devices) with excitation at 340 nm and emission at 475 nm at 30 °C every 38 s for 1 h. The linear section of the measurement was used to calculate initial velocity via linear regression in Prism 9.

#### Cathepsin B, Cathepsin K, and Cathepsin L

Recombinant cathepsin B, cathepsin K, and cathepsin L at 0.5, 0.042, and 0.25 nM, respectively, were pre-incubated with **4d** at various concentrations in the appropriate reaction buffers for 20 min. Z-FR-AMC fluorogenic peptide substrate was then added to the pre-treated cathepsin B, cathepsin K, and cathepsin L at 10, 5, and 10 μM, respectively, to initiate the enzymatic reactions. The fluorescence signal was monitored using EnVision with excitation at 355 nm and emission at 460 nm at room temperature every 5 min interval for 2 h. The linear portion of the measurement was used to calculate the initial velocity via Excel. Curve fits were performed using Prism 9. The reaction buffer used in the assay against cathepsin B contained 25 mM MES pH 6, 50 mM NaCl, 0.005% Brij35, 5 mM DTT, and 1% DMSO. The reaction buffer used in the assay against cathepsin K contained 50 mM NaOAc, pH 5.5, 5 mM ethylenediaminetetraacetic acid (EDTA), 0.005% Triton X-100, 5 mM dithiothreitol (DTT), and 1% dimethyl sulfoxide (DMSO). The reaction buffer used in the assay against cathepsin L contained 400 mM NaOAc, pH 5.5, 4 mM EDTA, 8 mM DTT, and 1% DMSO.

#### LC–MS/MS Analysis

Recombinantly purified M^pro^ (34 μg, 10 μM) was treated with DMSO or excess **4d** (100 μM) for 4 h at 30°C. Chloroform-methanol precipitation was done, followed by resuspension of M^pro^ in 8 M urea prepared in 50 mM NH_4_HCO_3_, pH 7.8. The protein was then reduced with 5 mM DTT at 60 °C for 1 h and alkylated with 15 mM iodoacetamide for 40 min at room temperature in the dark. The reaction was diluted with 50 mM NH_4_HCO_3_, pH 7.8, to reduce the urea concentration to 1 M. M^pro^ was digested by trypsin (V5113, Promega) overnight at 37°C with a trypsin-to-protein ratio of 1:50 (w/w). The digested peptides were desalted using Pierce C18 tips, followed by drying via speedvac. Digestion with Glu-C, Sequencing Grade (Promega, V1651) was carried out in 50 mM sodium phosphate buffer pH 7.8 overnight at 37 °C with a Glu-C to protein ratio of 1:50 (w/w). The same desalting and speedvac steps were done, followed by LC–MS/MS analysis. LC–MS/MS analysis was performed with an EASY-nLC 1200 (Thermo Fisher Scientific, San Jose, CA) coupled to an Orbitrap Eclipse Tribrid mass spectrometer (Thermo Fisher Scientific, San Jose, CA). Peptides were separated on an Aurora UHPLC Column (25 cm × 75 μm, 1.6 μm C18, AUR2-25075C18A, Ion Opticks) with a flow rate of 0.35 μL/min for a total duration of 135 min and ionized at 1.6 kV in the positive ion mode. The gradient was composed of 6% solvent B (7.5 min), 6–25% B (82.5 min), 25–40% B (30 min), and 40–98% B (15 min); solvent A: 0.1% formic acid in water; solvent B: 80% ACN and 0.1% formic acid. MS1 scans were acquired at a resolution of 120,000 from 350 to 2000 *m*/*z*, an AGC target of 1e6, and a maximum injection time of 50 ms. MS2 scans were acquired in the ion trap using the fast scan rate on precursors with 2–7 charge states and the quadrupole isolation mode (isolation window: 0.7 *m*/*z*) with higher-energy collisional dissociation (HCD, 30%) activation type. Dynamic exclusion was set to 30 s. The temperature of the ion transfer tube was 300 °C and the S-lens RF level was set to 30. MS2 fragmentation spectra were searched with Proteome Discoverer SEQUEST (version 2.5, Thermo Scientific) against the in silico tryptic-digested Uniprot Human herpesvirus 1 (HHV-1) database. The maximum missed cleavages were set to 2. Dynamic modifications were set to oxidation on methionine (M, +15.995 Da), phosphoribosylation (D, E, R and K, +212.009 Da), deamidation (N and Q, +0.984 Da), protein N-terminal acetylation (+42.011 Da), and Met-loss (−131.040 Da). Carbamidomethylation on cysteine residues (C, +57.021 Da) was set as a fixed modification. The maximum parental mass error was set to 10 ppm, and the MS2 mass tolerance was set to 0.6 Da. The false discovery threshold was set strictly to 0.01 using the percolator node validated by *q*-value. The relative abundance of parental peptides was calculated by integrating the area under the curve of the MS1 peaks using the Minora LFQ node.

#### Kinetic Measurements of Covalent Inactivation of M^pro^

The FRET substrate (25 μM) was first diluted in the reaction buffer containing compounds **4d** or **4i** at concentrations ranging from 10 nM to 10 μM. M^pro^ (0.05 μg, 0.015 μM) was then added to initiate the reaction, with the fluorescence changes being continuously monitored for 1 to 3 h. The progress curves were fit to a slow-binding Morrison equation, as described previously.^52,53^ The kinetic *k*_inact_/*K*_I_ parameter was derived using Prism 9.

### Crystallization and Structure Determination

M^pro^ protein was diluted to ∼7 mg/mL in the same buffer before the inhibitor (**3d** or **4d**) was added to the protein solution at about a 1:5 molar ratio of protein to inhibitor. The protein-inhibitor solution was incubated for 18 h at 4 °C. After incubation, the sample was spun down at 12,000 rpm for 2 min to remove the precipitate. Hanging drop trays were set up in an 18 °C room with 1 μL sample mixed with 1 μL crystallization solution composed of 0.1 M MIB pH 5.5 and 29%(w/v) PEG 1500 (**3d**). Hanging drop trays were set up in a 4 C room with drops made with 1.5 μL solution and with 1 μL crystallization solution composed of 0.1 MIB pH 6.5, 13% (w/v) PEG 1500, and 10% MPD (**4d**). Crystals grew overnight and were harvested seven days later using a 20% ethylene glycol cryobuffer.

Diffraction data for M^pro^ in complex with **3d** and **4d** were collected at beamlines 23ID-D and 23ID-B, respectively, at the National Institute of General Medical Sciences and the National Cancer Institute Structural Biology Facility at the Advanced Photon Source. A complete dataset was collected for each structure and processed using the DIALS data processing pipeline on the APS server, including indexing, integration, and scaling. Molecular replacement was performed using the Phaser-MR program from the PHENIX package, while model rebuilding and refinement were carried out using COOT and Phenix for simulated annealing and refinement, respectively. Ligands were generated using the eLBOW program in Phenix based on their SMILES code. The search model for molecular replacement was M^pro^ with nirmatrelvir (PDB: 7RFS) with the ligand removed. The initial phases of the MR protein model were improved by cyclic model building and refinement until a good model for each of the complexes was achieved. The final models for M^pro^ in complex with **3d** and **4d** were solved at a resolution range of 1.94 and 2.00 Å, respectively.

### In Vitro Labeling of Recombinant M^pro^ by Clickable Probes

Recombinant M^pro^ was incubated with probes **Alk-4d** or **Alk-4i** at indicated concentrations at 30 °C for 1 h or with the incubation time indicated for the time-course experiment. Next, click chemistry was performed at a final concentration of 25 μM TAMRA-azide, 1 mM tris(2-carboxyethyl)phosphine (TCEP, Thermo Scientific), 100 μM Tris-hydroxypropyltriazolylmethylamine (THPTA, Sigma-Aldrich), and 1 mM CuSO_4_ (Sigma-Aldrich) in a total volume of 21 μL. The reactions were carried out at room temperature for 1 h in the dark. Click reactions were terminated by the addition of 7 μL of 4× Laemmli sample loading buffer (Bio-Rad), boiled for 7 min, and resolved onto 4–20% sodium dodecyl-sulfate polyacrylamide gel electrophoresis (SDS-PAGE) gel (Bio-Rad). Fluorescence was visualized at 532 nm for excitation and at 600 nm for emission on a Typhoon 9400 Variable Mode Imager (GE Healthcare), and images were displayed as grayscale. After fluorescence scanning, protein loadings were visualized by silver staining using the Thermo Scientific Pierce Silver Stain Kit. Competition labeling involved a cotreatment of recombinant M^pro^ (1 μg) with a competitor (nirmatrelvir, **4d**, or **4i**) and a clickable probe (**Alk-4d** or **Alk-4i**) at indicated concentrations at 30 °C for 1 h. All subsequent steps on click reaction and gel imaging were the same as those above.

#### Pulse-Chase Style Competition Labeling for Measuring Nirmatrelvir Residence Time

Recombinant M^pro^ was first incubated with 1.5 μΜ of nirmatrelvir at 30 °C for 1 h, followed by treatment with the **Alk-4i** probe at 30 μM for different periods of time. All subsequent steps on click reaction and gel imaging were the same as those above.

### Measurement of M^pro^ Engagement Using Clickable Probes in Cells

HEK293T or HeLa cells were grown in 6-well plates and transfected with an appropriate plasmid harboring M^pro^. At 24 h post-transfection, the growth media [Dulbecco’s modified Eagle medium (DMEM) supplemented with 10% fetal bovine serum (FBS)] was aspirated off, and the cells were treated with fresh media containing various concentrations of probe (1000× stock solution in DMSO) or vehicle control for the indicated time. For competitive labeling experiments, cells were first incubated with the inhibitor at various concentrations for 1 h, washed with fresh, warm medium three times, and then treated with the probe at the appropriate concentration for another hour. After probe treatment, the medium was aspirated off, and the cells were washed twice with ice-cold Dulbecco’s phosphate-buffered saline. The cells were harvested, and the pellet was resuspended in 100 μL of NP40 lysis buffer (50 mM HEPES, pH 7.4, 1% NP-40, 150 mM NaCl) with a protease inhibitor cocktail (Roche). The lysate was incubated on ice for 20 min and fractionated by centrifugation at 18,000*g* for 10 min. The protein concentration was measured from each of the supernatant samples by a BCA assay (Pierce) and normalized to 1 mg/mL. All subsequent steps on click reaction and gel imaging were the same as those above.

### Cell Viability and Cell-Based Antiviral Assays

SARS-CoV-2 infection was performed at the UCLA BSL3 facility. The recombinant SARS-CoV-2 (icSARS-CoV-2-mNG) expressing mNeonGreen^54^ was a kind gift from the World Reference Center for Emerging Viruses and Arboviruses (WRCEVA) at the University of Texas Medical Branch. Hela-ACE2 cells were grown in standard DMEM with 10% FBS and 1% penicillin/streptomycin (GIBCO) at 37 °C in a 5% CO2 humidified atmosphere. SARS-CoV-2 infection was performed, as described previously.^51^ Briefly, Hela-ACE2 cells were infected with SARS-CoV-2 (MOI: 0.01) and incubated at 37 °C. The viral inoculum was removed after 1 h and replaced with fresh, complete DMEM media, followed by treating cells with the serially diluted compounds. At 24 h post-infection, the media was collected for evaluating the viral particles released to the media, and cell lysate was evaluated for quantification of the viral RNA transcripts by using RT-qPCR. Cells were collected in TRIzol, and RNA was isolated by standard isopropanol precipitation. 1 μg of RNA was reverse transcribed using iScript (BioRad) according to the manufacturer’s protocols by using random hexamers as primers. RT-qPCR analysis was done using the iCycler thermocycler (Bio-Rad). RT-qPCR was conducted in a final volume of 20 μL. Amplification conditions were 95 °C (3 min), 40 cycles of 95 °C (20 s), 55 °C (30 s), 72 °C (20 s). The expression values from untreated control cells were used to obtain the relative fold change, which was normalized to the ribosomal RNA L32 values. For detection of SARS-CoV-2 genomic RNA, the below primers targeting SARS-CoV-2 nucleocapsid protein (NP) were used: NP-fwd, 5′-TAATCAGACAAGGAACTGATTA-3′; NP rev, 5′-CGAAGGTGTGACTTCCATG-3′ RT-qPCR cycling conditions were 95 °C for 30 s and 40 cycles of 95 °C for 5 s, followed by 55 °C for 30 s.

Evaluating the 50% cytotoxic concentration (CC_50_) by treating cells with serially diluted compounds. Twenty hours prior to the cytotoxicity assay, 2 × 10^4^ Hela-ACE-2 cells were seeded in 96 Well white/clear bottom plate, TC Surface (Thermo Fisher). Cells were treated with serially diluted compounds. The cell viability was determined by using the Cell Titer-Glo Luminescent Cell Viability Assay (Promega). IC_50_ and CC_50_ values were calculated by non-linear regression analysis using GraphPad 5, where applicable.

## Supporting Information Available

Representative gel image of fractions after the purification of M^pro^ with RESOURCE Q column, dose responses of the compounds’ inhibition of M^pro^ in FRET-based cleavage assays, LC–MS/MS analysis and identification of site-specific modification of M^pro^ using **4d**, measurements of target engagement of M^pro^ in vitro and in situ using clickable probes, chemoproteomic labeling of **4d** in mammalian cell lines, measurement of drug-target residence time using clickable probe of **4d**, synthetic methods including detailed procedures and synthetic schemes, and ^1^H NMR characterization of all compounds prepared for this study, purity analyses of representative compounds via HPLC detailing HPLC chromatograms with ≥95% purity (PDF)
Molecular formula strings of compounds 1a-g, 2d, 2g, 3d, 3g, 4d-i, Alk-4d, and Alk-4i(CSV)
Protein Data Bank (PDB) IDs for the crystal structures of Mpro in complex with 3d and 4d are 8FY7 and 8FY6, respectively. Authors will release the atomic coordinates upon article publication

## Abbreviation

SARS-CoV-2: severe acute respiratory syndrome coronavirus 2
COVID-19: coronavirus disease 2019
M^pro^: severe acute respiratory syndrome coronavirus 2 main protease
RNA: ribonucleic acid
kb: kilobase
CYP3A: cytochrome P450, family 3, subfamily A
FRET: fluorescence resonance energy transfer
EWD: electron withdrawing group
Ni-NTA: nickle-nitrilotriacetic acid
PDB: protein data bank
RMSD: root mean square deviation
HEK293T: human embryonic kidney 293 cells containing SV40 large T antigen
HeLa: A cancer cell line from Henrietta Lacks, a cervical cancer patient
HeLa-ACE2: Hela cells constitutively expressing ACE2
MOI: multiplicity of infection
NMR: nuclear magnetic resonance
HPLC: high-performance liquid chromatography
LC–MS/MS: liquid chromatography tandem mass spectrometry
DTT: dithiothreitol
