# Supplementary material for: Alkyne as a Latent Warhead to Covalently Target SARS-CoV-2 Main Protease
Source: J Med Chem. 2023 Aug 18;66(17):12237–48. doi: 10.1021/acs.jmedchem.3c00810 (PMC10510381; doi:10.1021/acs.jmedchem.3c00810)
Supplement: Supplementary file 1 — jm3c00810_si_001.pdf [file jm3c00810_si_001.pdf]

Supporting Information

Alkyne as a Latent Warhead to Covalently Target SARS-CoV-2 Main Protease

Chau Ngo[1], William Fried[2], Saba Aliyari[3], Joshua Feng[1], Chao Qin[4], Shilei Zhang[3], Hanjing Yang[2], Jean Shanaa[3], Pinghui Feng[4], Genhong Cheng[3], Xiaojiang S. Chen[2], and Chao Zhang[1]*

1. Department of Chemistry and Loker Hydrocarbon Research Institute, University of Southern California, Los Angeles, California 90089, United States
2. Molecular and Computational Biology, Department of Biological Sciences, University of Southern California, Los Angeles, California 90089, United States
3. Department of Microbiology, Immunology and Molecular Genetics, University of California, Los Angeles, Los Angeles, California 90095, United States
4. Section of Infection and Immunity, Herman Ostrow School of Dentistry, University of Southern California, Los Angeles, California 90089, United States

*Email: zhang.chao@usc.edu

Table of Contents

1. Supporting Figures S2-S6
2. Synthetic Methods S7-S31
3. ^{1}H-NMR Characterization of All compounds Prepared for This Study S32-S50
4. Purity Analyses of Representative Compounds via HPLC S51-S55

1. Supporting Figures

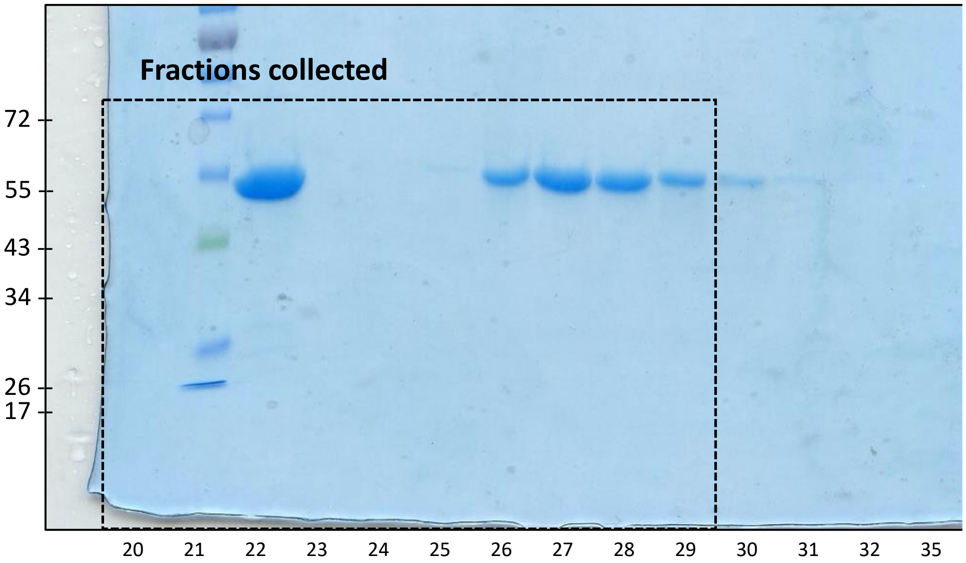

Figure S1. A representative gel image of fractions after the purification of M^{pro} with RESOURCE Q column. SDS-PAGE gel was stained with Coomassie Blue before being imaged with a ChemiDoc^{TM} MP Imaging System.

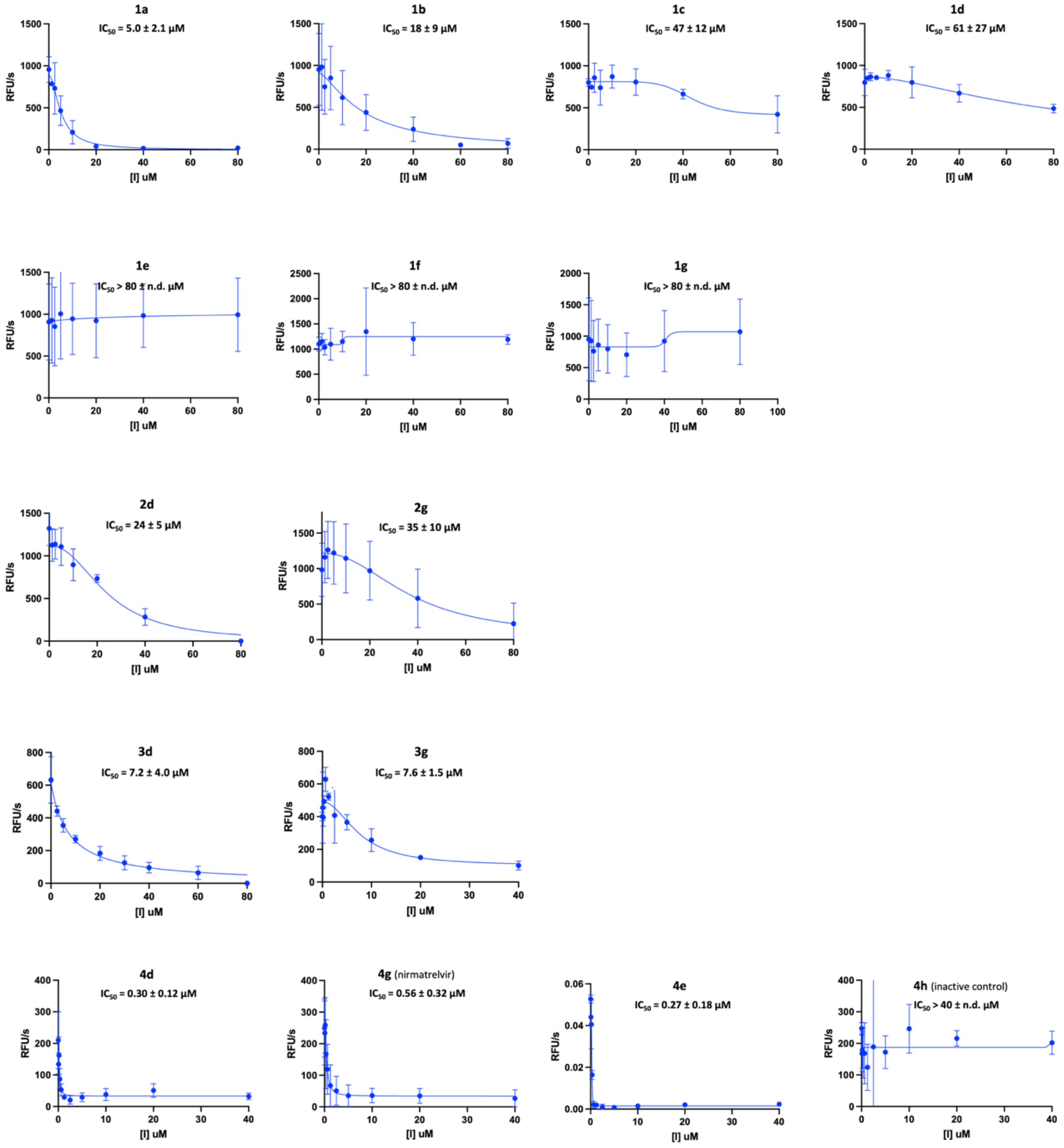

Figure S2. Dose responses of the compounds' inhibition of M^{pro} in FRET-based cleavage assays. The compound was added to the enzyme for 15 min prior to initiation of the assay via substrate addition.

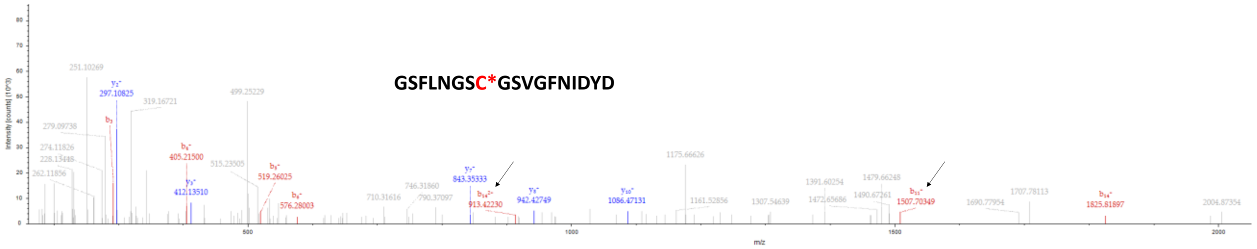

Figure S3. LC-MS/MS analysis to identify the site of modification in M^{pro} by 4d. Recombinant M^{pro} was incubated with **4d** for 4 hours before reduction, alkylation, digestion with trypsin and GluC, and LC-MS/MS analysis. A peptide containing **4d**-modified Cys^{145} (shown as C*) was detected by MS.

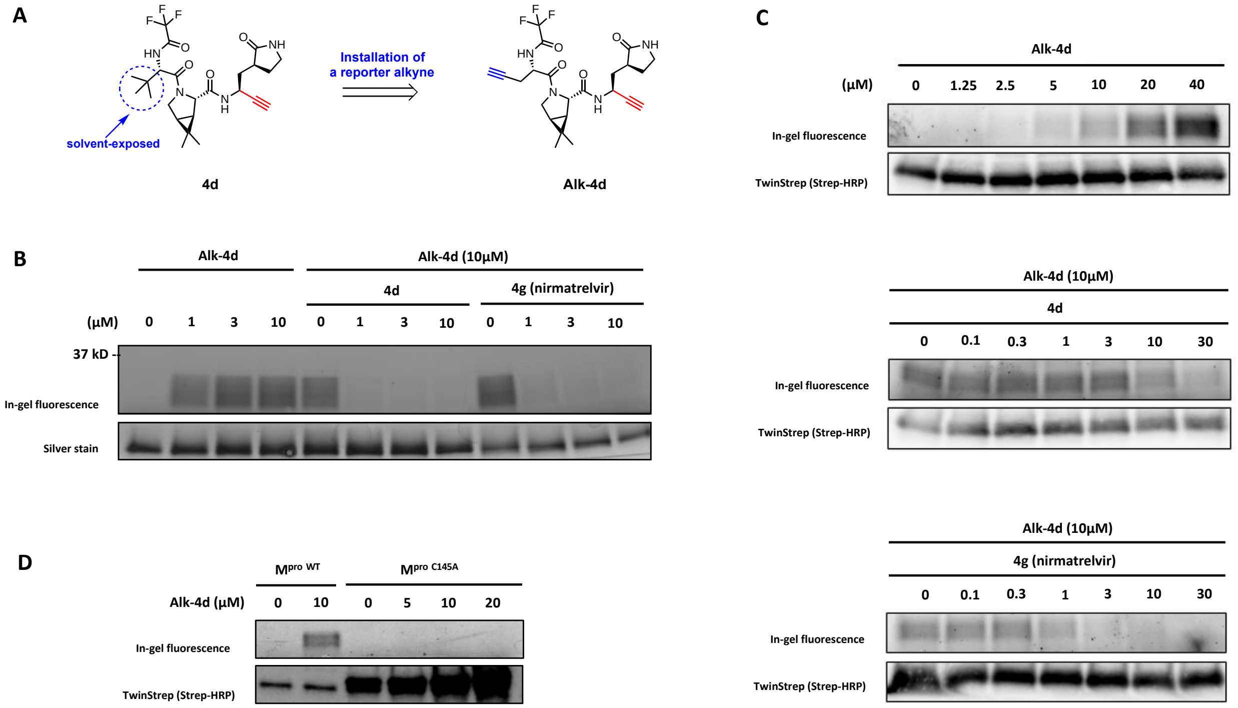

Figure S4. Use of a clickable probe Alk-4d to measure engagement to M^{pro} *in vitro* and *in situ*. **(A)** Design and chemical structure of the clickable probe, **Alk-4d**. **(B)** *In vitro* labeling of M^{pro} using **Alk-4d** in an in-gel fluorescence experiment. **(C)** *In situ* labeling of M^{pro} expressed in HEK293T cells using **Alk-4d**, along with competitors in an in-gel fluorescence experiment. **(D)** *In situ* labeling of M^{pro} by **Alk-4d** is dependent on Cys145 in cells. 2xStrep-tagged M^{pro} (wildtype or C145A mutant) were transfected to HEK293 cells prior to probe treatment.

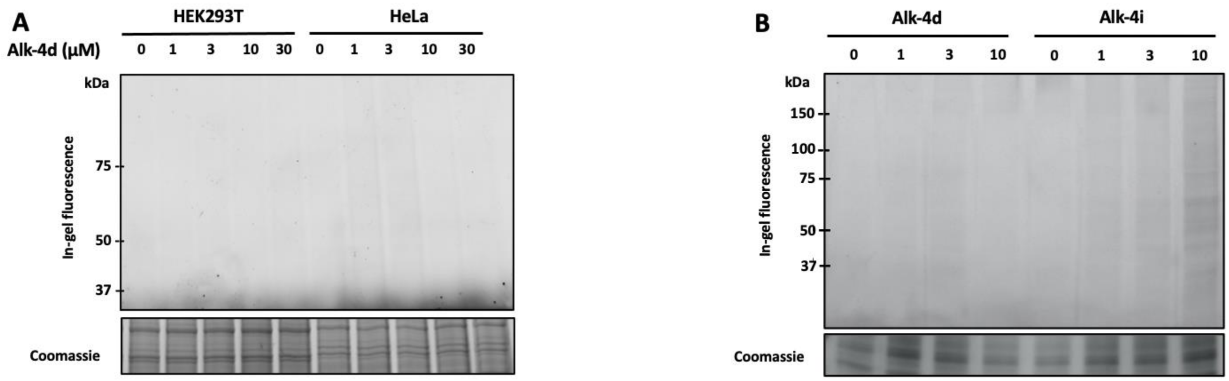

Figure S5. Proteomic labeling of live cells by Alk-4d and Alk-4i revealed in in-gel fluorescence experiments. (A) HEK293T and HeLa cells were treated with **Alk-4d** for 1 hour before cells are harvested and processed for in-gel fluorescence analysis. Up to 30 µM of **Alk-4d** produced little labeling of proteomes from HEK293T and HeLa cells. **(B)** HEK293T cells were treated with either **Alk-4d** or **Alk-4i** at indicated concentrations for 5 hours before being harvested and processed for in-gel fluorescence analysis. While **Alk-4d** at all tested concentrations caused little proteome labeling, 10 µM of **Alk-4i** induced significant labeling of HEK293T proteome.

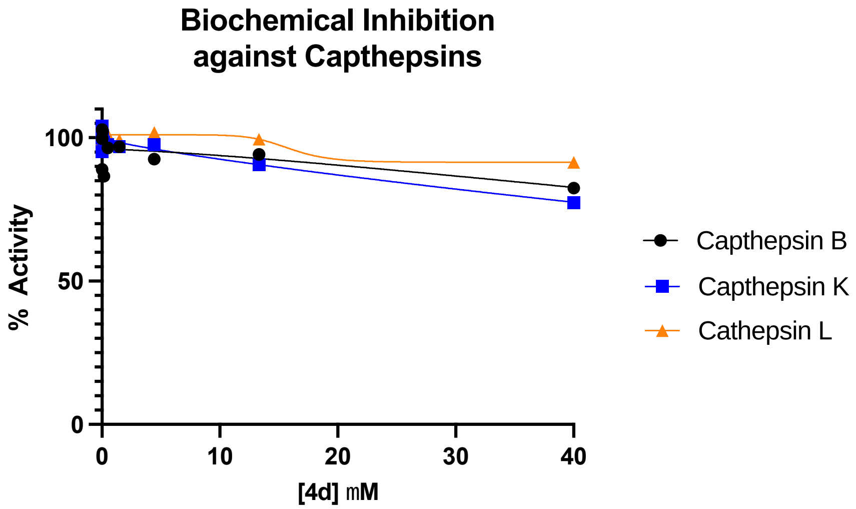

Figure S6. Dose-response curves of 4d against three human cathepsin proteases in biochemical assays. Recombinant cathepsin was incubated with **4d** for 20 minutes prior to initiation of the protease assay. Up to 40 µM of **4d** caused less than 20% inhibition of these cathepsin proteases.

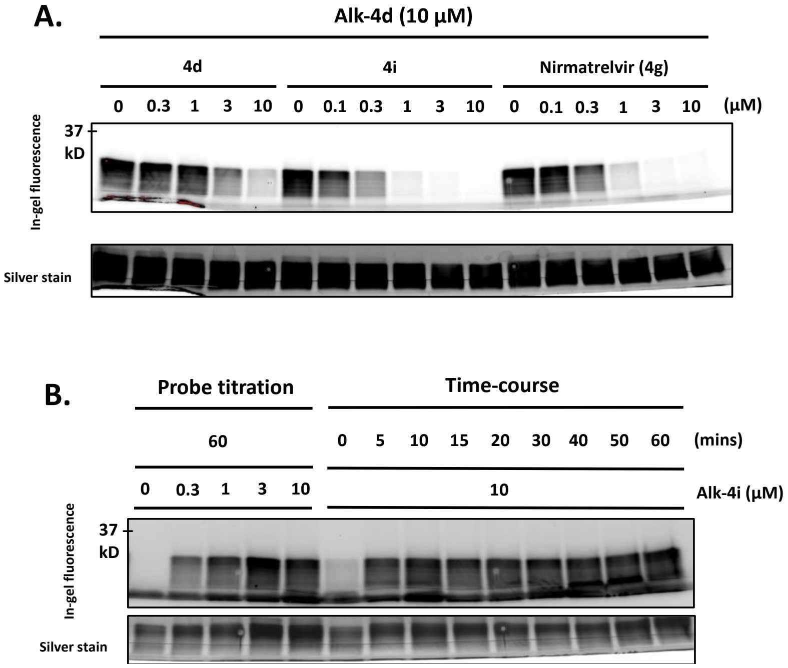

Figure S7. Use of a clickable probe Alk-4i to monitor engagement to M^{pro} *in vitro.* (**A**) Competitive labeling experiment using **Alk-4i** revealed that **4i** had comparable potency to nirmatrelvir and higher potency than **4d** at engaging M^{pro}; (**B**) **Alk-4i** afforded potent and rapid labeling of recombinant M^{pro}.

2. Synthetic Methods

Scheme S1. Synthesis of Compound 1a

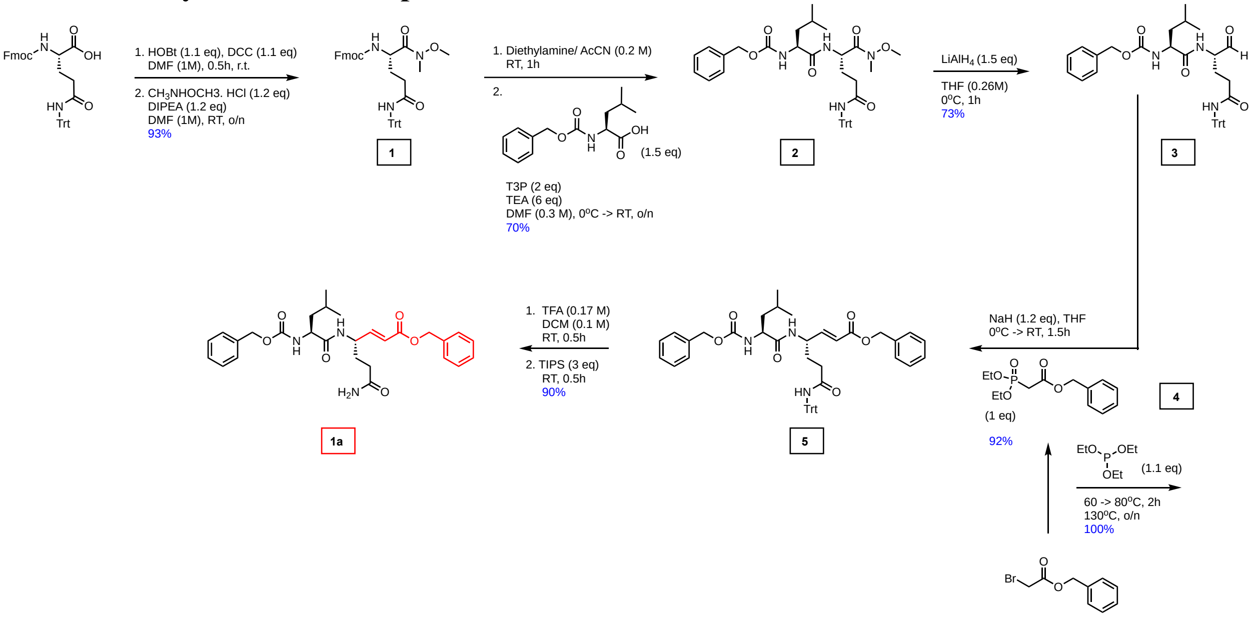

(9H-fluoren-9-yl)methyl (S)-(1-(methoxy(methyl)amino)-1,5-dioxo-5-(tritylamino)pentan-2-yl)carbamate (**1**)

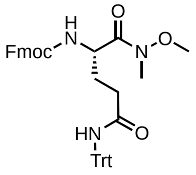

To a dried flask flushed with nitrogen was added Fmoc-Gln(Trt)-OH (4.09 mmol), N-hydroxybenzotriazole (0.608 g, 4.50 mmol), dicyclohexylcarbodiimide (0.929 g, 4.50 mmol) and DMF (4.1 ml). After stirring for 30 min at room temperature, a solid formed and was removed by filtration. N,O-dimethylhydroxylamine (0.479 g, 4.91 mmol), N,N'-diisopropylethylamine (DIPEA) (0.856 mL, 4.91 mmol) and another DMF (4.1 ml) were added. After stirring overnight, the mixture was concentrated. The crude oil was dissolved in ethyl acetate and washed with saturated sodium bicarbonate, 5% citric acid (by weight), and brine. The organic layer was dried (Na_2SO4), concentrated, and further purified by flash chromatography to yield a white solid (2.50 g, 93%).

^{1}H NMR (400 MHz, cdcl$_3$) δ 7.76 (d, J = 7.4 Hz, 2H), 7.57 (d, J = 7.6 Hz, 2H), 7.39 (d, J = 7.4 Hz, 2H), 7.29 (d, J = 7.5 Hz, 8H), 7.23 (d, J = 7.2 Hz, 9H), 6.93 (s, 1H), 5.72 – 5.61 (m, 1H), 4.77 (s, 1H), 4.38 (d, J = 7.2 Hz, 2H), 4.21 (d, J = 7.0 Hz, 1H), 3.64 (s, 3H), 3.18 (s, 3H), 2.35 (d, J = 7.2 Hz, 2H), 2.15 (s, 1H), 1.83 (s, 1H).

benzyl ((S)-1-(((S)-1-(methoxy(methyl)amino)-1,5-dioxo-5-(tritylamino)pentan-2-yl)amino)-4-methyl-1-oxopentan-2-yl)carbamate (**2**)

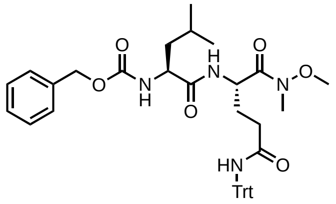

To a dried flask with **1** (2.67 g, 4.09 mmol) was added a mixture of diethylamine and acetonitrile (1:1, v/v; 20 mL). The Fmoc deprotection reaction was stirred at room temperature for 1 hour. The mixture was concentrated and further dried under high vacuum pressure. To the dried flask containing Fmoc-deprotected intermediate was added Z-Leu-OH (1.63 g, 6.14 mmol) and DMF (13.6 mL) followed by the addition of triethylamine (3.42 mL, 24.6 mmol). The reaction mixture was then cooled to 0°C using an ice water bath and stirred for 10 minutes. Propanephosphonic acid anhydride (50% in DMF; 2.38 mL, 8.19 mmol) was added dropwise to the solution at 0°C. The reaction mixture was stirred on ice for another 10 minutes and at room temperature overnight. Upon completion, the mixture was washed with brine (3 times). The organic layer was combined, washed with Na_2SO_4, and further purified by flash chromatography to yield a white solid (1.95 g, 70%).

^{1}H NMR (600 MHz, cdcl$_3$) δ 7.37 – 7.25 (m, 8H), 7.22 (ddt, *J* = 14.8, 11.1, 4.7 Hz, 12H), 7.03 (s, 1H), 6.74 (d, *J* = 8.4 Hz, 1H), 5.09 (d, *J* = 7.7 Hz, 1H), 5.03 – 4.86 (m, 3H), 4.13 (s, 1H), 3.64 (s, 3H), 3.16 (s, 3H), 2.41 – 2.29 (m, 2H), 2.18 (s, 1H), 1.80 (s, 1H), 1.66 (dt, *J* = 12.9, 6.5 Hz, 1H), 1.62 – 1.57 (m, 1H), 1.51 (dd, *J* = 9.3, 5.0 Hz, 1H), 0.92 (d, *J* = 6.5 Hz, 6H).

benzyl ((S)-1-(((S)-1,5-dioxo-5-(tritylamino)pentan-2-yl)amino)-4-methyl-1-oxopentan-2-yl)carbamate (**3**)

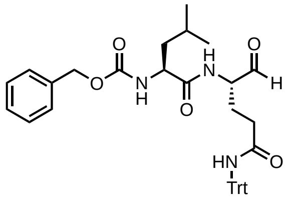

To a dried flask flushed with nitrogen was added **2** (1.10 g, 1.62 mmol), dissolved in anhydrous THF (6.35 mL). The reaction flask was further charged with nitrogen using a nitrogen balloon and cooled to 0ºC using an ice water bath. Under a nitrogen atmosphere, lithium aluminum hydride ($LiAlH_4$) (2.0 M in THF; 1.21 mL, 2.43 mmol) was added drop-wisely to the reaction mixture. A change to a bright orange color was observed within 1 minutes after completing $LiAlH_4$ addition. The reaction mixture was stirred on ice for 0.5-1 hour and quenched with saturated sodium potassium tartrate upon completion. The crude mixture was washed with brine washes (3 times), dried over Na_2SO_4, and further purified by flash chromatography to yield a white solid (0.732 g, 73%).

^{1}H NMR (400 MHz, cdcl$_3$) δ 9.41 (s, 1H), 7.29 (d, *J* = 8.3 Hz, 10H), 7.20 (d, *J* = 7.5 Hz, 10H), 7.04 (d, *J* = 6.5 Hz, 1H), 6.93 (s, 1H), 5.00 (q, *J* = 11.2 Hz, 4H), 4.27 (s, 1H), 2.50 – 2.17 (m, 4H), 1.94 – 1.85 (m, 1H), 1.47 (q, *J* = 9.3 Hz, 2H), 0.92 (d, *J* = 6.5 Hz, 6H).

benzyl 2-(diethoxyphosphoryl)acetate (**4**)

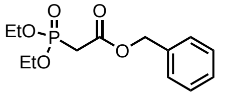

To a dried flask was added benzyl-2-bromoacetate (0.692 mL, 4.37 mmol) and triethyl phosphite (0.823 mL, 4.80 mmol). The reaction mixture was then heated to 80ºC for 1 hour to remove ethyl bromide through distillation. The reaction was then stirred at 130ºC overnight. Upon completion, the crude mixture was washed with $NaHCO_3$, brine, dried over Na_2SO_4, and further purified by flash chromatography to yield the final product (1.24g, ~100%).

^{1}H NMR (400 MHz, cdcl$_3$) δ 7.41 – 7.29 (m, 5H), 5.18 (s, 2H), 4.12 (p, *J* = 7.3 Hz, 4H), 3.09 – 2.93 (m, 2H), 1.29 (t, *J* = 7.1 Hz, 6H).

benzyl (S,E)-4-((S)-2-(((benzyloxy)carbonyl)amino)-4-methylpentanamido)-7-oxo-7-(tritylamino)hept-2-enoate (**5**)

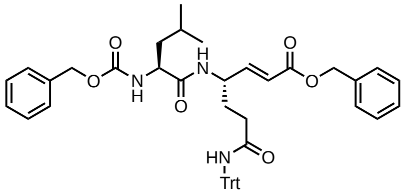

To a cold solution of benzyl 2-(diethoxyphophoryl)acetate **4** (41.2 mg, 0.143 mmol) in THF (0.26 ml) at 0ºC was added sodium hydride (6.27 mg, 0.157 mmol) under nitrogen. After 30-min stirring, **3** (81 mg, 0.131 mmol) dissolved in THF (0.26 mL) was added. The reaction was stirred for another 1.5 hours at room temperature and quenched with 5% $KHSO_4$ solution (0.52 ml). The reaction mixture was washed with brine and brine washes (3 times), dried over Na_2SO_4, and further purified by flash chromatography to yield a white solid (90.3 mg, 92%).

^{1}H NMR (400 MHz, cdcl$_3$) δ 7.40 – 7.28 (m, 12H), 7.20 (dt, *J* = 15.3, 7.3 Hz, 12H), 6.96 (d, *J* = 7.8 Hz, 1H), 6.82 (q, *J* = 5.8 Hz, 2H), 5.89 (t, *J* = 14.0 Hz, 1H), 5.16 (s, 2H), 5.11 – 4.99 (m, 2H), 4.94 (d, *J* = 9.2 Hz, 2H), 4.54 (s, 1H), 4.06 (s, 1H), 2.35 (tt, *J* = 17.3, 8.2 Hz, 2H), 1.95 (s, 1H), 1.60 (d, *J* = 11.5 Hz, 3H), 1.41 (dd, *J* = 12.4, 7.1 Hz, 1H), 0.94 – 0.87 (m, 6H).

benzyl (S,E)-7-amino-4-((S)-2-(((benzyloxy)carbonyl)amino)-4-methylpentanamido)-7-oxohept-2-enoate (**1a**)

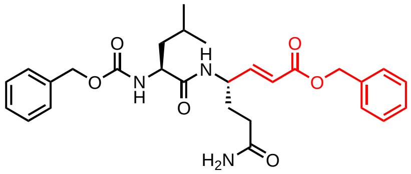

To a dried flask was added **5** (90 mg, 0.12 mmol) in dry dichloromethane (DCM) (1.20 mL). Trifluoroacetic acid (0.72 mL) was then added to the reaction mixture quickly. The reaction was then stirred for 30 minutes at room temperature. Bright yellow solution was observed. After 30-minute stirring, triisopropylsilane (73.6 μL, 0.36 mmol) was added, and the reaction was stirred for another 30 minutes at room temperature. The reaction mixture was observed with a color change from yellow to clear. Upon completion, the acidic crude mixture was neutralized with $NaHCO_3$. The neutral reaction mixture was then washed with brine, dried over Na_2SO_4, and further purified by flash chromatography to yield a white solid (55 mg, 90%).

^{1}H NMR (400 MHz, cdcl$_3$) δ 7.38 – 7.28 (m, 10H), 6.98 (t, *J* = 11.1 Hz, 1H), 6.85 (dd, *J* = 15.8, 5.1 Hz, 1H), 5.96 (s, 1H), 5.93 (s, 1H), 5.61 (s, 1H), 5.43 (d, *J* = 8.0 Hz, 1H), 5.16 (d, *J* = 5.6 Hz, 2H), 5.09 (d, *J* = 13.4 Hz, 2H), 4.59 (t, *J* = 9.2 Hz, 1H), 4.19 (q, *J* = 8.0 Hz, 1H), 2.20 (dh, *J* = 15.3, 7.3 Hz, 2H), 1.96 (s, 1H), 1.90 – 1.75 (m, 1H), 1.65 (dt, *J* = 17.4, 9.3 Hz, 2H), 1.53 (s, 1H), 0.93 (d, *J* = 5.7 Hz, 6H).

Scheme S2. Synthesis of Compound 1b

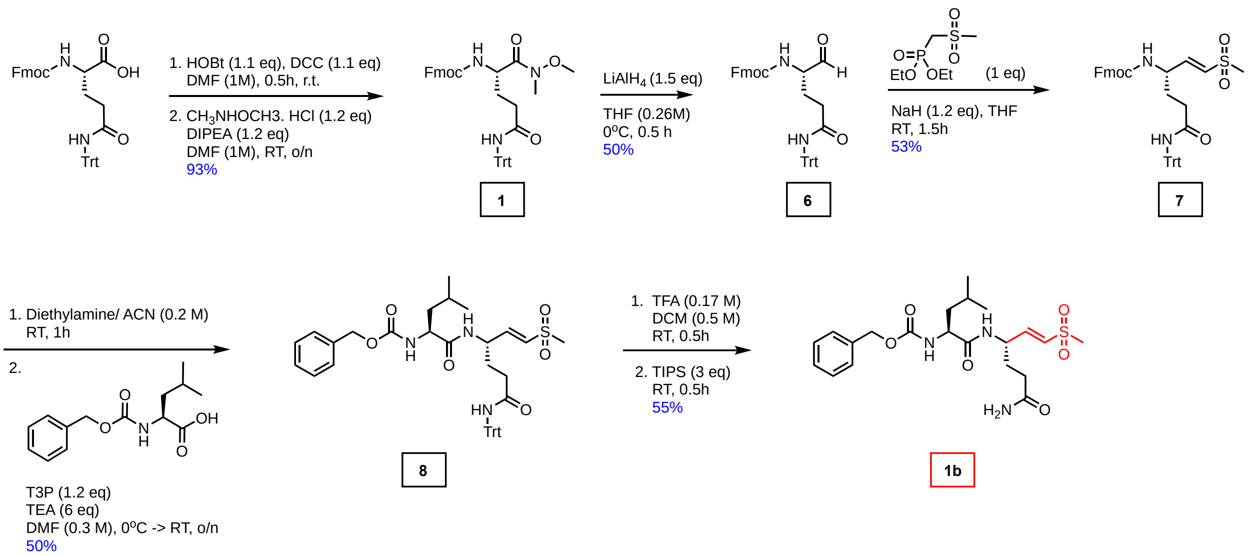

<u>*(9H-fluoren-9-yl)methyl (S)-(1,5-dioxo-5-(tritylamino)pentan-2-yl)carbamate*</u> (**6**)

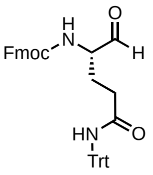

Similar to the synthetic procedure for compound **3**. Yielded white solid (0.78 g, 50%).

^{1}H NMR (600 MHz, cdcl$_3$) δ 9.44 (s, 1H), 7.73 – 7.68 (m, 2H), 7.57 (d, *J* = 7.6 Hz, 2H), 7.41 – 7.35 (m, 2H), 7.32 – 7.26 (m, 7H), 7.25 – 7.21 (m, 4H), 7.19 (d, *J* = 7.7 Hz, 6H), 6.79 (s, 1H), 5.56 (d, *J* = 6.7 Hz, 1H), 4.45 (t, *J* = 7.2 Hz, 2H), 4.20 (t, *J* = 6.6 Hz, 1H), 4.15 – 4.12 (m, 1H), 2.42 – 2.27 (m, 2H), 2.22 (d, *J* = 5.9 Hz, 1H), 1.82 (dd, *J* = 14.4, 7.4 Hz, 1H).

<u>*(9H-fluoren-9-yl)methyl (S,E)-(1-(methylsulfonyl)-6-oxo-6-(tritylamino)hex-1-en-3-yl)carbamate*</u> (**7**)

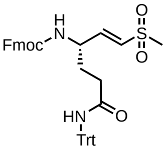

Similar to the synthetic procedure for compound **5** with the use of diethyl (methylsulfonyl)methylphosphonate. Yielded a white/ pale yellow solid (0.48 g, 53%).

^{1}H NMR (600 MHz, cdcl$_3$) δ 7.73 (dd, *J* = 7.7, 2.3 Hz, 2H), 7.55 (t, *J* = 7.0 Hz, 2H), 7.38 (q, *J* = 7.5 Hz, 2H), 7.30 – 7.26 (m, 8H), 7.25 – 7.22 (m, 3H), 7.20 – 7.15 (m, 6H), 6.76 (dd, *J* = 15.0, 4.9 Hz, 1H), 6.67 (s, 1H), 6.36 (d, *J* = 15.1 Hz, 1H), 5.48 (d, *J* = 7.7 Hz, 1H), 4.49 – 4.38 (m, 2H), 4.34 (s, 1H), 4.18 (t, *J* = 6.6 Hz, 1H), 2.88 (s, 3H), 2.35 (s, 2H), 1.98 (s, 1H), 1.84 (d, *J* = 6.8 Hz, 1H).

<u>*benzyl((S)-4-methyl-1-(((S,E)-1-(methylsulfonyl)-6-oxo-6-(tritylamino)hex-1-en-3-yl)amino)-1-oxopentan-2-yl)carbamate*</u> (**8**)

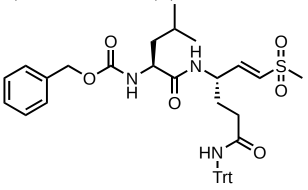

Similar to the synthetic procedure for compound **2**. Yielded white solid (25.1 mg, 50%).

^{1}H NMR (400 MHz, cdcl$_3$) δ 7.58 (d, *J* = 7.2 Hz, 1H), 7.35 – 7.26 (m, 10H), 7.22 (d, *J* = 1.8 Hz, 2H), 7.17 (dd, *J* = 8.1, 1.7 Hz, 8H), 6.80 (s, 1H), 6.75 (dd, *J* = 15.0, 4.8 Hz, 1H), 6.45 (d, *J* = 15.1 Hz, 1H), 5.03 (d, *J* = 12.3 Hz, 1H), 4.89 (d, *J* = 12.2 Hz, 1H), 4.73 (d, *J* = 7.8 Hz, 1H), 4.53 (s, 1H), 4.06 (d, *J* = 11.2 Hz, 1H), 2.89 (d, *J* = 3.8 Hz, 3H), 2.51 – 2.41 (m, 1H), 2.40 – 2.29 (m, 1H), 1.68 – 1.55 (m, 4H), 0.98 – 0.89 (m, 6H).

benzyl ((S)-1-(((S,E)-6-amino-1-(methylsulfonyl)-6-oxohex-1-en-3-yl)amino)-4-methyl-1-oxopentan-2-yl)carbamate (**1b**)

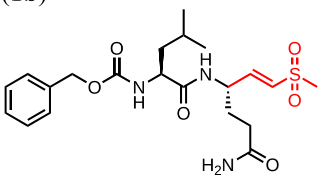

Similar to the synthetic procedure for compound **1a**. Yielded white (10.8 g, 55%).

^{1}H NMR (400 MHz, cdcl$_3$) δ 7.58 (s, 1H), 7.32 (s, 5H), 6.79 (d, *J* = 14.7 Hz, 1H), 6.56 (d, *J* = 15.1 Hz, 1H), 6.14 (s, 1H), 6.02 (s, 1H), 5.64 (s, 1H), 5.09 (s, 2H), 4.66 (s, 1H), 4.24 (s, 1H), 2.92 (s, 3H), 2.25 (s, 3H), 2.01 – 1.82 (m, 2H), 0.93 (s, 6H).

Scheme S3. Synthesis of Compound 1c

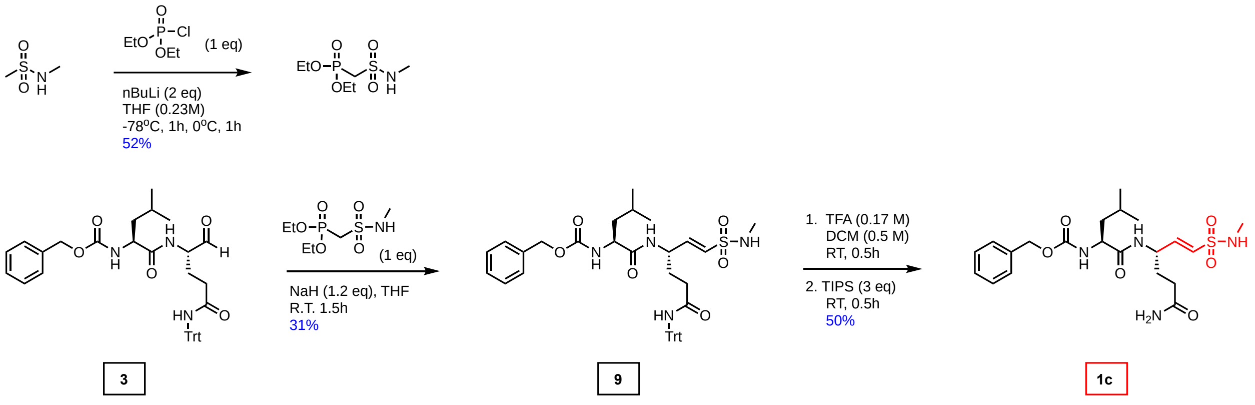

---

diethyl ((N-methylsulfamoyl)methyl)phosphonate

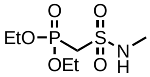

To a dried flask flushed with nitrogen was added N-methylmethanesulfonamide (600 mg, 5.50 mmol) in anhydrous THF (24 mL). The reaction flask was further charged with nitrogen using a nitrogen balloon. At -78°C in an acetone-dry ice bath, n-butyllithium was added dropwisely to the reaction mixture followed by 1-hour stirring at -78°C. After 1 hour, diethyl chlorophosphonate was then added slowly, and an ice water bath was used to allow the reaction to reach to 0°C. The reaction was then stirred for another 1 hour at 0°C followed by washing with ammonium chloride NH_4Cl (3 times), brine, dried over Na_2SO_4, and purified using flash chromatography, which yielded the product as clear oil (699.5 mg, 52%)

^{1}H NMR (600 MHz, cdcl$_3$) δ 5.26 (d, J = 6.7 Hz, 1H), 4.26 – 4.17 (m, 4H), 3.59 (d, J = 16.2 Hz, 2H), 2.84 – 2.81 (m, 3H), 1.36 (t, J = 7.0 Hz, 6H).

benzyl((S)-4-methyl-1-(((S,E)-1-(N-methylsulfamoyl)-6-oxo-6-(tritylamino)hex-1-en-3-yl)amino)-1-oxopentan-2-yl)carbamate (**9**)

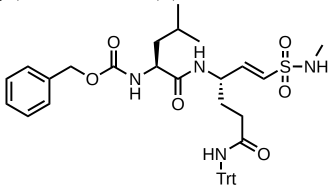

Similar to the synthetic procedure for compound **5**. Yielded white solid (17.4 mg, 31%).

^{1}H NMR (600 MHz, cdcl$_3$) δ 7.27 (d, J = 7.0 Hz, 9H), 7.24 – 7.21 (m, 5H), 7.18 (t, J = 8.0 Hz, 7H), 6.55 (td, J = 14.2, 4.8 Hz, 1H), 6.21 (t, J = 13.1 Hz, 1H), 5.12 – 4.99 (m, 2H), 4.91 (q, J = 12.4 Hz, 1H), 4.59 – 4.42 (m, 2H), 4.22 (dtq, J = 10.0, 6.2, 3.4 Hz, 1H), 4.03 (qd, J = 8.2, 4.1 Hz, 1H), 2.63 – 2.53 (m, 3H), 2.39 – 2.26 (m, 2H), 1.95 – 1.89 (m, 1H), 1.81 (s, 1H), 1.72 (dt, J = 15.9, 6.6 Hz, 1H), 1.59 – 1.53 (m, 2H), 0.93 – 0.87 (m, 6H).

benzyl((*S*)-1-(((*S*,*E*)-6-amino-1-(*N*-methylsulfamoyl)-6-oxohex-1-en-3-yl)amino)-4-methyl-1-oxopentan-2-yl)carbamate (**1c**)

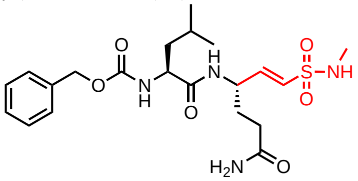

Similar to the synthetic procedure for compound **1a**. Yielded white solid (5.7 mg, 50%).

^{1}H NMR (400 MHz, cdcl$_3$) δ 7.36 (s, 6H), 6.67 – 6.57 (m, 1H), 6.31 (d, J = 15.5 Hz, 1H), 5.89 – 5.61 (m, 1H), 5.56 – 5.31 (m, 1H), 5.21 (s, 1H), 5.10 (d, J = 9.5 Hz, 2H), 4.61 (s, 1H), 4.32 (s, 1H), 4.15 (s, 1H), 2.76 – 2.59 (m, 3H), 2.27 (s, 2H), 2.03 (d, J = 13.7 Hz, 1H), 1.89 (s, 2H), 1.53 (d, J = 9.3 Hz, 2H), 0.95 (d, J = 6.2 Hz, 6H).

Scheme S4. Synthesis of Compound 1d

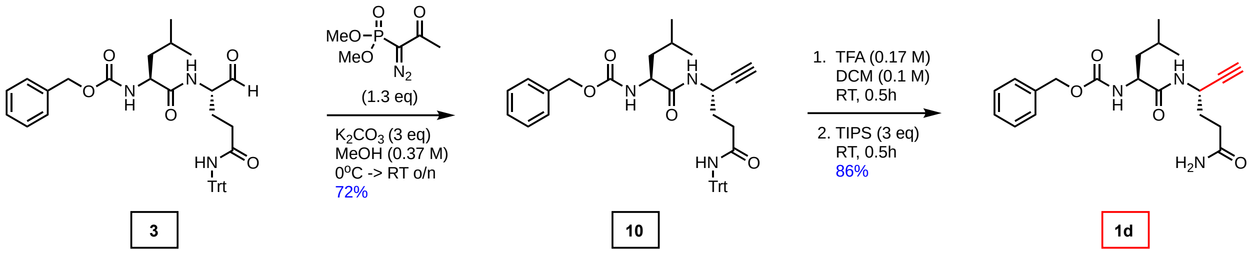

benzyl ((S)-4-methyl-1-oxo-1-(((S)-6-oxo-6-(tritylamino)hex-1-yn-3-yl)amino)pentan-2-yl)carbamate (**10**)

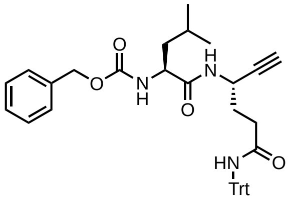

Compound **3** (79.2 mg, 0.127 mmol) was dissolved in dry methanol (0.346 mL). To the reaction flask was quickly added potassium carbonate (53.0 mg, 0.383 mmol). After 3-5 mins stirring, Bestmann-Ohira reagent was added dropwisely. The reaction was stirred overnight at room temperature. Upon completion, bicarbonate and brine washes were performed, followed by Na_2SO_4 drying and flash chromatography purification. The reaction yielded the product as a white solid (56.4 mg, 72%).

^{1}H NMR (400 MHz, cdcl$_3$) δ 7.33 (d, J = 8.5 Hz, 18H), 7.27 (s, 2H), 7.24 (s, 1H), 6.90 – 6.75 (m, 2H), 5.17 – 4.92 (m, 3H), 4.83 – 4.69 (m, 1H), 4.13 (s, 1H), 2.64 – 2.38 (m, 2H), 2.32 (s, 1H), 2.16 – 1.94 (m, 2H), 1.61 (s, 1H), 1.50 (d, J = 10.0 Hz, 1H), 0.96 (d, J = 5.6 Hz, 6H).

benzyl ((S)-1-(((S)-6-amino-6-oxohex-1-yn-3-yl)amino)-4-methyl-1-oxopentan-2-yl)carbamate (**1d**)

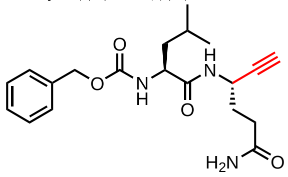

Similar to the synthetic procedure for compound **1a**. Yielded white solid (29.5 mg, 86%).

^{1}H NMR (400 MHz, cdcl$_3$) δ 7.34 (d, J = 4.8 Hz, 5H), 7.15 – 6.97 (m, 1H), 6.05 (s, 1H), 5.68 (s, 1H), 5.52 – 5.28 (m, 1H), 5.10 (d, J = 8.2 Hz, 2H), 4.73 (p, J = 7.6 Hz, 1H), 4.18 (q, J = 7.6 Hz, 1H), 2.41 – 2.25 (m, 3H), 2.00 (tt, J = 14.2, 6.7 Hz, 2H), 1.66 (dt, J = 14.6, 7.5 Hz, 2H), 1.57 – 1.48 (m, 1H), 0.93 (d, J = 5.9 Hz, 6H).

Scheme S5. Synthesis of Compound 1e

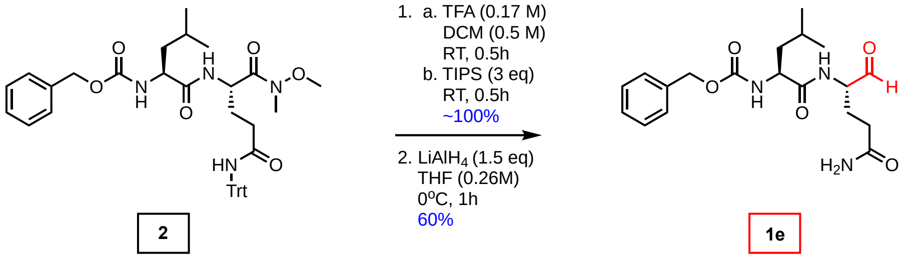

<u>*benzyl ((S)-1-(((S)-5-amino-1,5-dioxopentan-2-yl)amino)-4-methyl-1-oxopentan-2-yl)carbamate*</u> (**1e**)

Similar to the synthetic procedure for compound **1a** and **2**. Yielded white solid (21.8 mg, 60%).

^{1}H NMR (600 MHz, cdcl$_3$) δ 7.37 – 7.26 (m, 6H), 7.15 (s, 1H), 6.90 – 6.63 (m, 1H), 5.53 (s, 1H), 5.07 (q, *J* = 8.0 Hz, 2H), 4.85 (s, 1H), 4.28 – 4.06 (m, 2H), 2.40 (d, *J* = 9.1 Hz, 2H), 2.13 – 2.00 (m, 1H), 1.74 (s, 1H), 1.69 – 1.57 (m, 2H), 1.55 – 1.49 (m, 1H), 0.91 (t, *J* = 7.0 Hz, 6H).

Scheme S6. Synthesis of Compound 1f

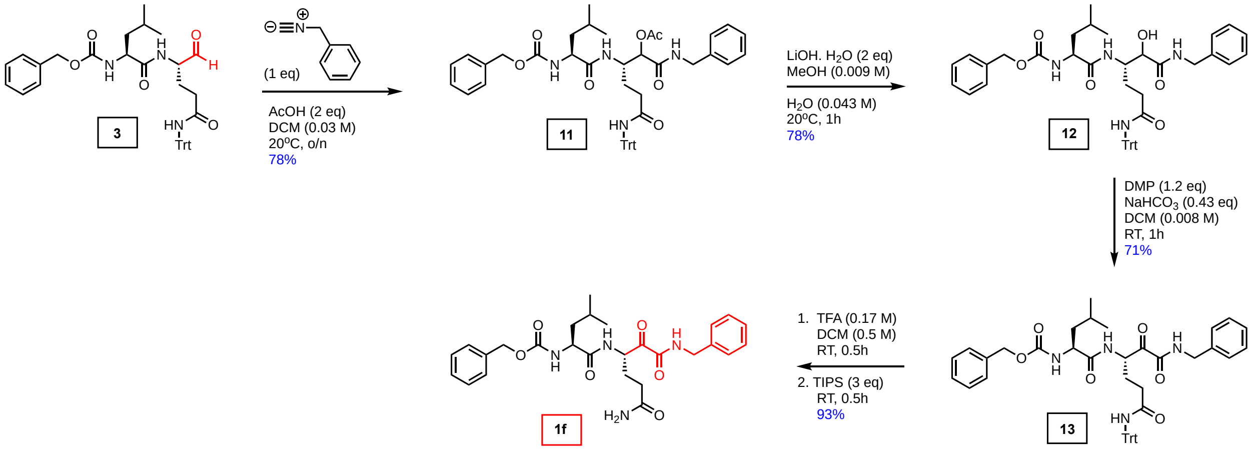

(5S,8S)-5-isobutyl-3,6,10-trioxo-8-(3-oxo-3-(tritylamino)propyl)-1,12-diphenyl-2-oxa-4,7,11-triazadodecan-9-yl acetate (**11**)

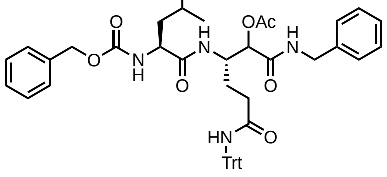

Acetic acid (38.8 mg, 0.65 mmol) and benzyl isocyanide (37.8 mg, 0.32 mmol) were added to the solution of compound **3** (200 mg, 0.32 mmol) in DCM (10.8 mL). The mixture was stirred at 20°C overnight. The reaction was concentrated and purified by chromatography on silica gel to give compound **11** (199.3 mg, 78%) as off-white solid.

^{1}H NMR (600 MHz, cdcl$_3$) δ 7.36 – 7.25 (m, 11H), 7.25 – 7.10 (m, 17H), 7.06 (s, 1H), 6.60 – 6.40 (m, 1H), 5.19 (d, *J* = 16.0 Hz, 1H), 5.09 – 4.84 (m, 3H), 4.46 – 4.32 (m, 2H), 4.23 (s, 1H), 4.07 (dq, *J* = 8.9, 5.2 Hz, 1H), 2.33 (s, 2H), 2.10 – 2.04 (m, 3H), 1.62 – 1.60 (m, 2H), 1.47 (dt, *J* = 9.3, 5.0 Hz, 1H), 0.98 – 0.87 (m, 6H).

benzyl((2S)-1-(((3S)-1-(benzylamino)-2-hydroxy-1,6-dioxo-6-(tritylamino)hexan-3-yl)amino)-4-methyl-1-oxopentan-2-yl)carbamate (**12**)

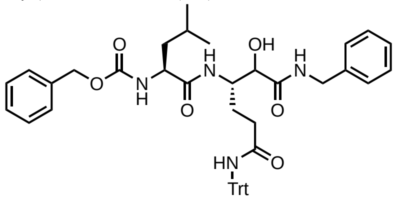

Compound **11** (199.3 mg, 0.250 mmol) was dissolved in MeOH (25 mL) and H_2O (5.8 mL). LiOH.H_2O (21 mg, 0.500 mmol) was added. The mixture was stirred at 20°C for 1 h. Then, the mixture was adjusted to pH = 6~7 with 1M HCl. Subsequently, the reaction was evaporated under vacuum, extracted using $NaHCO_3$ and brine. The organic phase was dried over Na_2SO_4 and purified by chromatography on silica gel to give compound **12** (146.9 mg, 78%) as light yellow solid.

^{1}H NMR (600 MHz, cdcl$_3$) δ 7.33 – 7.25 (m, 9H), 7.23 (d, *J* = 6.9 Hz, 8H), 7.21 – 7.16 (m, 10H), 7.10 (dq, *J* = 12.0, 6.1 Hz, 2H), 6.96 – 6.83 (m, 1H), 5.12 (d, *J* = 7.7 Hz, 1H), 5.05 – 4.84 (m, 3H), 4.42 (dd, *J* = 14.9, 6.3 Hz, 1H), 4.39 – 4.31 (m, 1H), 4.27 (dd, *J* = 14.8, 5.6 Hz, 1H), 4.09 – 3.99 (m, 2H), 3.86 (s, 1H), 2.36 (s, 2H), 1.99 – 1.91 (m, 1H), 1.74 (s, 1H), 1.59 (dd, *J* = 13.1, 6.5 Hz, 1H), 1.56 – 1.49 (m, 1H), 1.39 (ddd, *J* = 14.4, 9.3, 5.8 Hz, 1H), 0.89 (d, *J* = 5.9 Hz, 6H).

benzyl((S)-1-(((S)-1-(benzylamino)-1,2,6-trioxo-6-(tritylamino)hexan-3-yl)amino)-4-methyl-1-oxopentan-2-yl)carbamate (**13**)

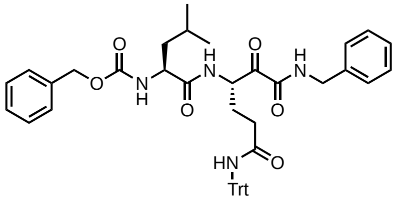

In a dried flask, compound **12** (147 mg, 0.195 mmol) was dissolved in anhydrous dichloromethane (DCM) (19.5 mL). To the reaction flask was then added Dess-Martin periodinane (100 mg, 0.237 mmol) and $NaHCO_3$ (7.03 mg, 0.084 mmol). The reaction mixture was stirred for one hour at room temperature. Upon completion, the crude mixture was washed with $NaHCO_3$, brine, dried over Na_2SO_4, and lastly purified using flash chromatography. The reaction yielded the product as off-white solid (104.6 mg, 71%).

^{1}H NMR (600 MHz, cdcl$_3$) δ 7.34 (d, J = 6.6 Hz, 1H), 7.28 (d, J = 6.5 Hz, 5H), 7.24 (d, J = 7.6 Hz, 7H), 7.23 – 7.14 (m, 12H), 7.03 (t, J = 6.1 Hz, 1H), 6.93 (d, J = 8.9 Hz, 1H), 5.23 (d, J = 8.5 Hz, 1H), 5.21 – 5.17 (m, 1H), 5.03 (d, J = 12.3 Hz, 1H), 4.96 (d, J = 12.5 Hz, 1H), 4.37 (t, J = 5.2 Hz, 2H), 4.21 – 4.14 (m, 1H), 2.39 (q, J = 5.9 Hz, 2H), 2.29 – 2.21 (m, 1H), 2.05 (s, 1H), 1.62 (tt, J = 14.8, 5.8 Hz, 2H), 1.43 (ddd, J = 14.2, 9.4, 5.4 Hz, 1H), 0.89 (q, J = 6.2 Hz, 6H).

benzyl((S)-1-(((S)-6-amino-1-(benzylamino)-1,2,6-trioxohexan-3-yl)amino)-4-methyl-1-oxopentan-2-yl)carbamate (**1f**)

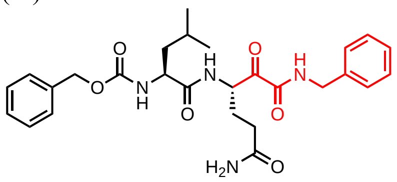

Similar to the synthetic procedure for compound **1a**. Yielded white solid (47.9 mg, 93%).

^{1}H NMR (400 MHz, cdcl$_3$) δ 7.76 (s, 1H), 7.40 (s, 1H), 7.29 (s, 5H), 7.22 (d, J = 8.0 Hz, 5H), 6.72 (s, 1H), 6.06 (s, 1H), 5.47 (s, 1H), 5.02 (d, J = 10.7 Hz, 2H), 4.47 (s, 1H), 4.41 – 4.18 (m, 2H), 4.13 (s, 1H), 2.30 (s, 2H), 2.00 (s, 1H), 1.70 (s, 1H), 1.59 (s, 1H), 1.43 (dd, J = 13.4, 7.0 Hz, 2H), 0.87 (d, J = 6.9 Hz, 6H).

Scheme S7. Synthesis of Compound 1g

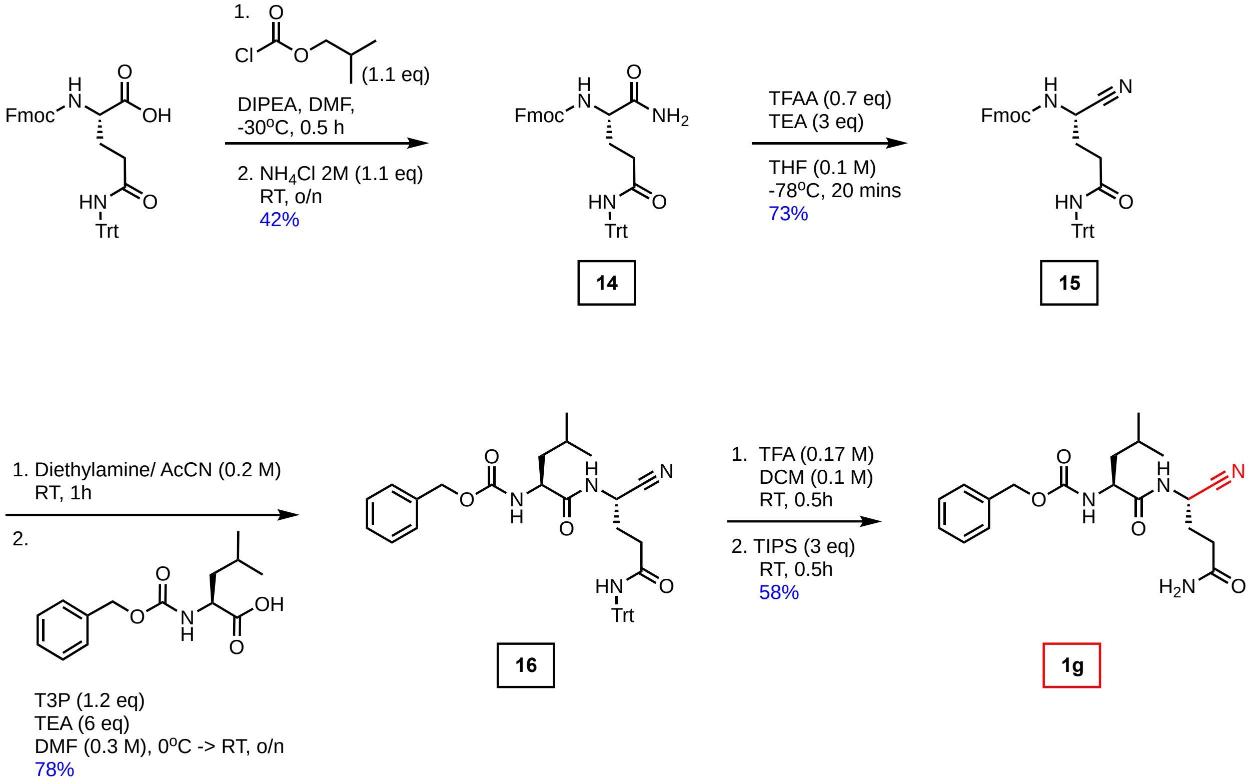

(9H-fluoren-9-yl)methyl (S)-(1-amino-1,5-dioxo-5-(tritylamino)pentan-2-yl)carbamate (**14**)

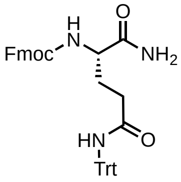

To Fmoc-Gln(Trt)-OH (1.003 g, 1.64 mmol) in dry DMF (6.57 mL) was added DIPEA (424.5 mg, 3.28 mmol) under N_2 atmosphere. Under an ice-water/ NaBr bath, isobutyl chloroformate was added dropwisely at ~-30°C. Fuming effect was observed after each addition. The reaction mixture was stirred for another 0.5h at -30°C followed by overnight stirring. The general workup procedure involving $NaHCO_3$ and brine washes, Na_2SO_4 drying, and purification by flash chromatography was done to yield compound **14** as white solid (423 mg, 42%)

^{1}H NMR (400 MHz, acetone) δ 7.98 (s, 1H), 7.84 (d, J = 7.6 Hz, 2H), 7.70 (d, J = 7.5 Hz, 2H), 7.39 (t, J = 7.5 Hz, 2H), 7.34 – 7.14 (m, 17H), 6.97 (s, 1H), 6.71 (d, J = 7.9 Hz, 1H), 6.52 (s, 1H), 4.33 (d, J = 7.2 Hz, 2H), 4.20 (dt, J = 13.6, 7.2 Hz, 2H), 2.63 – 2.38 (m, 2H), 2.10 (dt, J = 14.1, 6.0 Hz, 1H), 1.95 – 1.86 (m, 1H).

(9H-fluoren-9-yl)methyl (S)-(1-cyano-4-oxo-4-(tritylamino)butyl)carbamate (**15**)

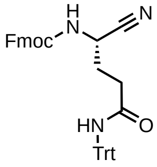

In a dried flask, compound **14** (50 mg, 0.082 mmol) was dissolved in dry THF. At 78°C under an acetone-dry ice bath, triethylamine (TEA) (34.3 µL, 0.25 mmol) was added quickly. The reaction mixture was stirred for 5 mins and was introduced with trifluoroacetic anhydride (TFAA) (8 µL, 0.057 mmol) drop-wisely at 78°C. The reaction was allowed stirring for another 15-20 mins and extracted with $NaHCO_3$, brine followed by $MgSO_4$ drying and flash chromatography purification to yield compound **15** as white solid (35.4 mg, 73%)

^{1}H NMR (400 MHz, acetone) δ 8.03 (s, 1H), 7.86 (dt, J = 7.6, 1.0 Hz, 2H), 7.68 (d, J = 7.7 Hz, 2H), 7.44 – 7.38 (m, 2H), 7.35 – 7.18 (m, 18H), 4.64 (q, J = 7.7 Hz, 1H), 4.42 (d, J = 7.0 Hz, 2H), 4.25 (t, J = 7.0 Hz, 1H), 2.72 – 2.57 (m, 2H), 2.11 (qd, J = 7.2, 2.6 Hz, 2H).

benzyl ((S)-1-(((S)-1-cyano-4-oxo-4-(tritylamino)butyl)amino)-4-methyl-1-oxopentan-2-yl)carbamate (**16**)

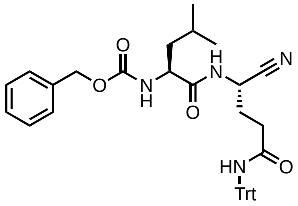

Similar to the synthetic procedure for compound **2**. Yielded white solid (24 mg, 78%).

^{1}H NMR (400 MHz, acetone) δ 8.11 (d, *J* = 7.9 Hz, 1H), 7.96 (s, 1H), 7.41 – 7.14 (m, 22H), 6.49 (d, *J* = 8.0 Hz, 1H), 5.04 (d, *J* = 12.5 Hz, 1H), 4.92 (d, *J* = 12.6 Hz, 1H), 4.84 (q, *J* = 7.7 Hz, 1H), 4.18 (q, *J* = 7.7 Hz, 1H), 2.60 (tq, *J* = 15.4, 7.4 Hz, 2H), 1.73 (dq, *J* = 13.1, 6.5 Hz, 1H), 1.60 (t, *J* = 7.2 Hz, 2H), 0.91 (dd, *J* = 9.9, 6.6 Hz, 6H).

benzyl ((S)-1-(((S)-4-amino-1-cyano-4-oxobutyl)amino)-4-methyl-1-oxopentan-2-yl)carbamate (**1g**)

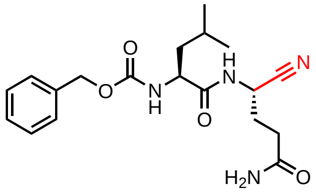

Similar to the synthetic procedure for compound **1a**. Yielded white solid (8.5 mg, 58%).

^{1}H NMR (400 MHz, acetone) δ 7.56 (d, *J* = 8.1 Hz, 1H), 7.42 – 7.27 (m, 5H), 7.05 (s, 1H), 6.64 (d, *J* = 7.5 Hz, 1H), 6.53 (s, 1H), 5.08 (s, 2H), 4.50 (td, *J* = 8.5, 4.7 Hz, 1H), 4.20 (q, *J* = 7.5 Hz, 1H), 2.49 (t, *J* = 7.6 Hz, 2H), 2.32 – 2.19 (m, 1H), 2.00 – 1.88 (m, 1H), 1.75 (dq, *J* = 13.1, 6.6 Hz, 1H), 1.65 (dd, *J* = 8.1, 5.3 Hz, 2H), 0.92 (t, *J* = 6.4 Hz, 6H).

Scheme S8. Synthesis of Compound 2d

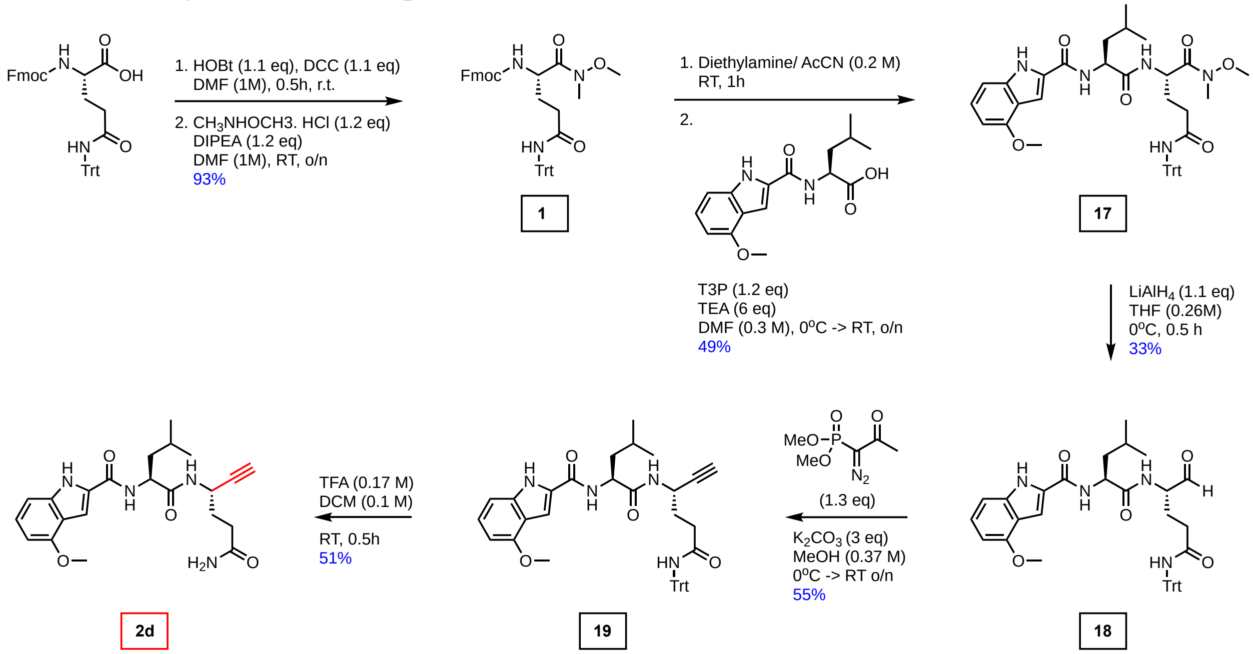

(S)-N1-methoxy-2-((S)-2-(4-methoxy-1H-indole-2-carboxamido)-4-methylpentanamido)-N1-methyl-N5-tritylpentanediamide (**17**)

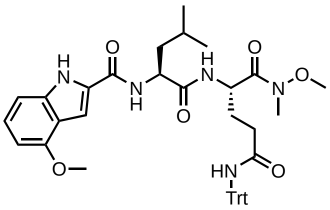

Similar to the synthetic procedure for compound **2**. Yielded white solid (96 mg, 49%).

^{1}H NMR (600 MHz, cdcl3) δ 10.78 (s, 1H), 8.55 – 8.39 (m, 1H), 7.98 (s, 1H), 7.23 (s, 1H), 7.14 (d, J = 8.4 Hz, 14H), 7.09 (p, J = 4.3 Hz, 4H), 7.01 (d, J = 8.3 Hz, 1H), 6.47 (d, J = 7.7 Hz, 1H), 5.24 – 5.14 (m, 2H), 3.93 (s, 3H), 3.60 (s, 3H), 3.27 (s, 3H), 2.24 (dq, J = 16.3, 6.6 Hz, 1H), 2.10 (d, J = 9.2 Hz, 2H), 1.78 – 1.65 (m, 4H), 0.90 (d, J = 5.5 Hz, 3H), 0.87 (d, J = 5.5 Hz, 3H).

N-((S)-1-(((S)-1,5-dioxo-5-(tritylamino)pentan-2-yl)amino)-4-methyl-1-oxopentan-2-yl)-4-methoxy-1H-indole-2-carboxamide (**18**)

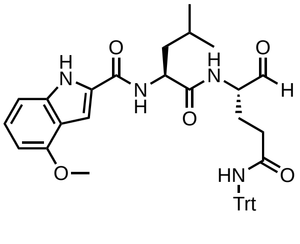

Similar to the synthetic procedure for compound **3**. Yielded white solid (21.3 mg, 33%).

^{1}H NMR (400 MHz, cdcl3) δ 9.42 (s, 1H), 9.17 (s, 1H), 7.50 (d, J = 6.8 Hz, 1H), 7.25 – 7.15 (m, 16H), 7.13 (d, J = 8.0 Hz, 1H), 7.05 (s, 1H), 6.93 (d, J = 2.2 Hz, 1H), 6.81 (d, J = 8.3 Hz, 1H), 6.55 (d, J = 8.0 Hz, 1H), 6.48 (d, J = 7.8 Hz, 1H), 4.68 (td, J = 8.6, 5.1 Hz, 1H), 4.34 – 4.25 (m, 1H), 3.91 (s, 3H), 2.46 – 2.28 (m, 2H), 2.19 (ddt, J = 12.9, 8.1, 5.1 Hz, 1H), 1.73 (tt, J = 15.3, 7.8 Hz, 4H), 0.95 (dd, J = 14.6, 6.0 Hz, 6H).

4-methoxy-N-((S)-4-methyl-1-oxo-1-(((S)-6-oxo-6-(tritylamino)hex-1-yn-3-yl)amino)pentan-2-yl)-1H-indole-2-carboxamide (**19**)

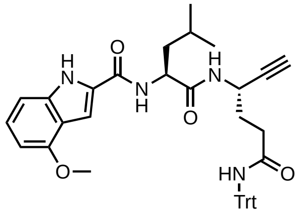

Similar to the synthetic procedure for compound **10**. Yielded white solid (11.6 mg, 55%).

^{1}H NMR (400 MHz, cdcl3) δ 9.10 (s, 1H), 7.25 – 7.07 (m, 17H), 6.97 (d, *J* = 2.2 Hz, 1H), 6.93 – 6.78 (m, 2H), 6.59 (s, 1H), 6.47 (t, *J* = 8.2 Hz, 1H), 4.73 – 4.62 (m, 1H), 4.57 (td, *J* = 8.6, 5.0 Hz, 1H), 3.90 (s, 3H), 2.41 (p, *J* = 6.9 Hz, 1H), 2.22 (d, *J* = 5.0 Hz, 1H), 1.93 (dd, *J* = 13.2, 6.5 Hz, 2H), 1.76 (dq, *J* = 11.7, 6.2 Hz, 2H), 1.66 – 1.55 (m, 2H), 1.01 – 0.87 (m, 6H).

N-((S)-1-(((S)-6-amino-6-oxohex-1-yn-3-yl)amino)-4-methyl-1-oxopentan-2-yl)-4-methoxy-1H-indole-2-carboxamide (**2d**)

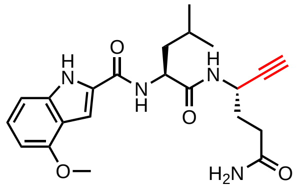

Similar to the synthetic procedure for compound **1a**. Compound **19** (9.4 mg, 0.014 mmol) was dissolved in DCM (143.6 μL). TFA (86 μL) was then added to the reaction flask at room temperature. The reaction was done after 30-minute and subjected to general procedure workup and flask chromatography purification to yielded the product as white solid (3 mg, 51%).

^{1}H NMR (400 MHz, cdcl3) δ 9.45 (s, 1H), 7.20 (t, *J* = 8.0 Hz, 1H), 7.15 – 7.05 (m, 2H), 7.01 (d, *J* = 8.3 Hz, 1H), 6.82 (d, *J* = 8.1 Hz, 1H), 6.50 (d, *J* = 7.8 Hz, 1H), 5.85 (s, 1H), 5.61 (s, 1H), 4.80 (d, *J* = 7.3 Hz, 1H), 4.66 (s, 1H), 3.93 (s, 3H), 2.41 (dt, *J* = 14.6, 6.9 Hz, 1H), 2.33 – 2.22 (m, 2H), 2.05 (d, *J* = 7.6 Hz, 2H), 1.81 – 1.65 (m, 3H), 0.96 (dd, *J* = 9.9, 5.6 Hz, 6H).

Scheme S9. Synthesis of Compound 2g

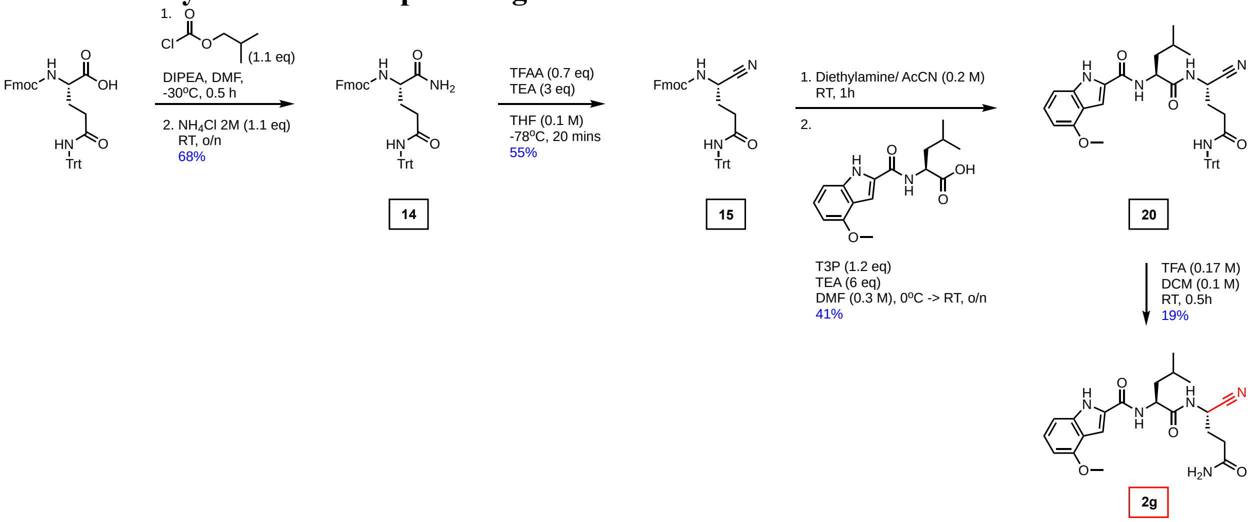

N-((S)-1-(((S)-1-cyano-4-oxo-4-(tritylamino)butyl)amino)-4-methyl-1-oxopentan-2-yl)-4-methoxy-1H-indole-2-carboxamide (**20**)

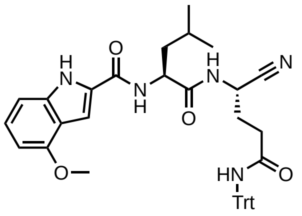

Similar to the synthetic procedure for compound **2**. Yielded white solid (31.5 mg, 41%).

^{1}H NMR (400 MHz, cdcl3) δ 9.62 (s, 1H), 8.08 (s, 1H), 7.25 – 7.15 (m, 12H), 7.15 – 7.11 (m, 5H), 7.02 – 6.96 (m, 2H), 6.94 – 6.83 (m, 2H), 6.42 (d, *J* = 7.8 Hz, 1H), 6.08 (d, *J* = 6.4 Hz, 1H), 4.69 – 4.37 (m, 2H), 3.94 (d, *J* = 1.2 Hz, 1H), 3.88 (s, 3H), 2.02 – 1.84 (m, 2H), 0.97 – 0.79 (m, 6H).

N-((S)-1-(((S)-4-amino-1-cyano-4-oxobutyl)amino)-4-methyl-1-oxopentan-2-yl)-4-methoxy-1H-indole-2-carboxamide (**2g**)

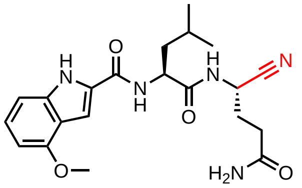

Similar to the synthetic procedure for compound **2d**. Yielded white solid (3.5 mg, 19%).

^{1}H NMR (400 MHz, cdcl3) δ 9.96 (s, 1H), 7.59 (s, 1H), 7.21 – 7.13 (m, 2H), 7.03 (d, *J* = 8.3 Hz, 1H), 6.93 (s, 1H), 6.69 (s, 1H), 6.49 (d, *J* = 7.7 Hz, 1H), 5.91 (s, 1H), 4.76 – 4.52 (m, 2H), 3.93 (s, 3H), 2.46 – 2.29 (m, 2H), 2.28 – 2.06 (m, 2H), 1.96 (d, *J* = 7.3 Hz, 1H), 1.80 (d, *J* = 9.9 Hz, 2H), 0.94 (dd, *J* = 10.3, 5.9 Hz, 6H).

Scheme S10. Synthesis of Compounds 3d-e

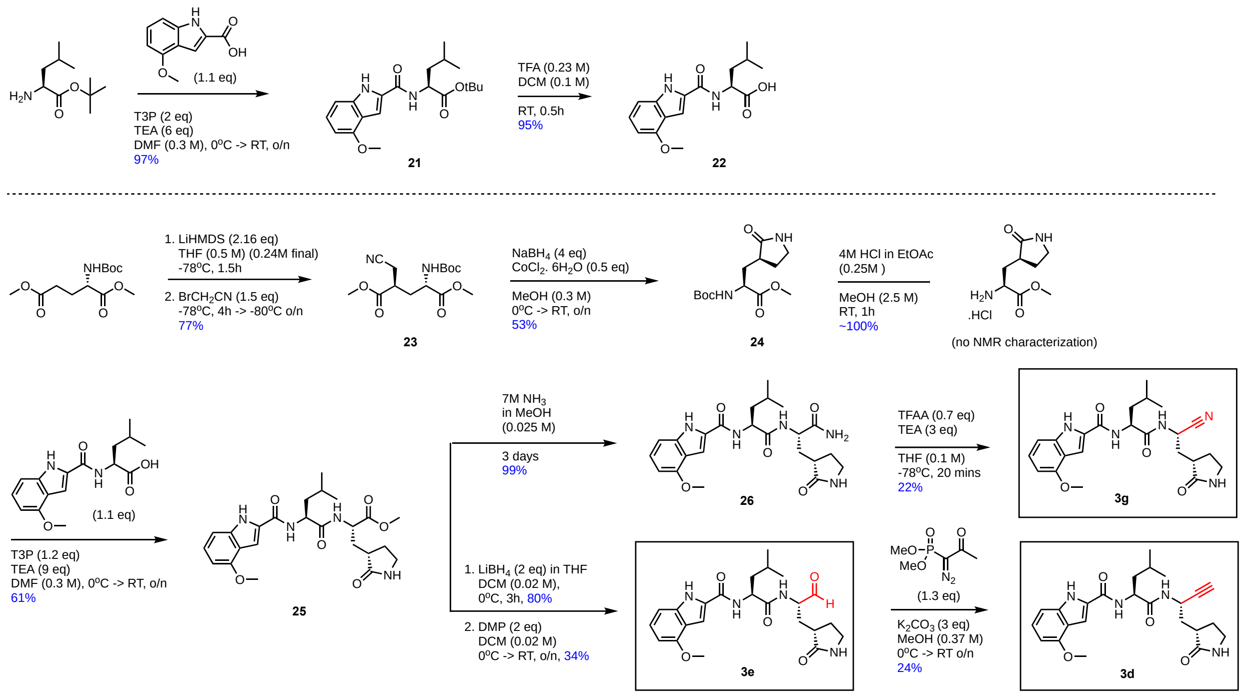

<u>*tert*-butyl (4-methoxy-1*H*-indole-2-carbonyl)-*L*-leucinate</u> (**21**)

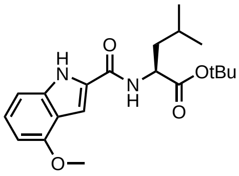

Similar to the synthetic procedure for compound **2**. Yielded orange solid (431.2 mg, 97%).

^{1}H NMR (400 MHz, cdcl$_3$) δ 9.37 (s, 1H), 7.20 (t, *J* = 8.0 Hz, 1H), 7.08 – 6.99 (m, 2H), 6.64 (d, *J* = 8.4 Hz, 1H), 6.50 (d, *J* = 7.7 Hz, 1H), 4.73 (td, *J* = 8.4, 5.0 Hz, 1H), 3.95 (s, 3H), 1.79 – 1.69 (m, 2H), 1.65 (dd, *J* = 10.5, 5.7 Hz, 1H), 0.99 (dd, *J* = 6.2, 3.6 Hz, 6H).

<u>*(4-methoxy-1H-indole-2-carbonyl)-L-leucine*</u> (**22**)

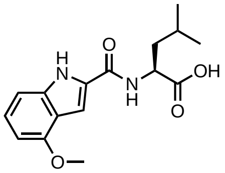

Similar to the synthetic procedure for compound **2a**. Yielded white solid (162.1 mg, 95%).

^{1}H NMR (400 MHz, cdcl$_3$) δ 9.85 (s, 1H), 7.19 (t, *J* = 8.0 Hz, 1H), 7.10 (d, *J* = 2.3 Hz, 1H), 7.02 (d, *J* = 8.3 Hz, 1H), 6.72 (d, *J* = 8.2 Hz, 1H), 6.49 (d, *J* = 7.7 Hz, 1H), 4.82 (td, *J* = 8.7, 4.4 Hz, 1H), 3.94 (s, 3H), 1.79 (h, *J* = 5.3 Hz, 2H), 1.73 – 1.65 (m, 1H), 0.96 (dd, *J* = 6.1, 2.6 Hz, 6H).

<u>*dimethyl (2S,4R)-2-((tert-butoxycarbonyl)amino)-4-(cyanomethyl)pentanedioate*</u> (**23**)

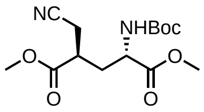

To a solution of *N*-Boc-l-(+)-glutamic acid dimethyl ester (2.338 g, 8.49 mmol) in THF (17 mL) was added dropwise a solution of lithium bis(trimethylsilyl)amide (LiHMDS) (1M in THF, 18.3 mL, 18.34 mmol) at −78°C under an nitrogen atmosphere. The resulting dark mixture was stirred at −78°C for 1 h. At the same time, bromoacetonitrile (887.4 µL, 12.7 mmol) was stirred with basic aluminum oxide for 2 h and then filtered. The freshly filtered bromoacetonitrile was added dropwise to the dianion solution over a period of 1 h while maintaining the temperature below −70°C. The reaction mixture was stirred at −78°C for additional 1–2 h and at -80°C overnight. The reaction was quenched with pre-cooled methanol (1.17 ml) in one portion and stirred for 30 min. The resulting methoxide was then quenched with a pre-cooled acetic acid in

THF solution (1.06 ml HOAc/ 7.02 mL THF) in one portion. After stirring for 30 min, the cooling bath was removed and replaced with water bath. The reaction mixture was allowed to warm up to 0°C and then poured into a brine solution in a separatory funnel. The layers were separated, and the organic layer was concentrated to afford a dark brown oil. Silica gel (800 g), activated carbon (200 g) and methylene chloride (2 L) were added to the Rotovap flask and spun on a Rotovap for 1 h without heat and vacuum. The slurry was then filtered and washed with another 2 L of methylene chloride. The light brown filtrate was concentrated to afford a light brown oil (2.048 g, 77% crude yield). The crude product was used in the next step without further purification.

^{1}H NMR (400 MHz, cdcl$_3$) δ 5.10 (d, J = 8.5 Hz, 1H), 4.38 (s, 1H), 3.76 (d, J = 4.1 Hz, 6H), 2.86 (dd, J = 12.9, 6.6 Hz, 1H), 2.78 (t, J = 5.1 Hz, 2H), 2.24 – 2.09 (m, 2H), 1.44 (s, 9H).

methyl (S)-2-((tert-butoxycarbonyl)amino)-3-((S)-2-oxopyrrolidin-3-yl)propanoate (**24**)

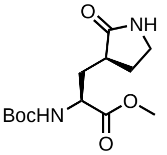

To the dried flask with compound **23** (2.048 g, 6.52 mmol) in dry methanol (MeOH) (21.7 mL) was added cobalt(II) chloride hexahydrate (CoCl. 6H_2O) (774.9 mg, 3.26 mmol). After 5-min stirring on ice, sodium borohydride ($NaBH_4$) (985.6 mg, 26 mmol) was added to the reaction mixture in portions over 30 minutes. The reaction was stirred overnight at room temperature. After completion, the solvent was removed, and the crude mixture quenched with 1M citric acid followed by washing with $NaHCO_3$ and brine, drying over Na_2SO_4 and purifying by flash chromatography to yield white solid (985 mg, 53%)

^{1}H NMR (400 MHz, cdcl$_3$) δ 5.58 (s, 1H), 5.45 (d, J = 8.5 Hz, 1H), 4.33 (s, 1H), 3.74 (s, 3H), 3.35 (dd, J = 8.5, 5.8 Hz, 2H), 2.46 (dq, J = 13.0, 6.5 Hz, 2H), 2.14 (td, J = 12.4, 3.7 Hz, 1H), 1.85 (ddt, J = 13.4, 8.7, 5.2 Hz, 2H), 1.44 (s, 9H).

methyl(S)-2-((S)-2-(4-methoxy-1H-indole-2-carboxamido)-4-methylpentanamido)-3-((S)-2-oxopyrrolidin-3-yl)propanoate (**25**)

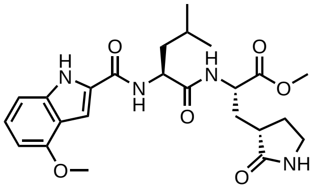

Similar to the synthetic procedure for compound **2**. Yielded pale yellow solid (389.8 mg, 61%).

^{1}H NMR (400 MHz, cdcl$_3$) δ 9.27 (s, 1H), 8.03 (d, J = 7.9 Hz, 1H), 7.19 (t, J = 8.0 Hz, 1H), 7.08 (d, J = 2.2 Hz, 1H), 7.01 (d, J = 8.4 Hz, 1H), 6.75 (s, 1H), 6.50 (d, J = 7.8 Hz, 1H), 5.80 (s, 1H), 4.85 – 4.74 (m, 1H), 4.51 (dt, J = 11.1, 5.0 Hz, 1H), 3.93 (d, J = 8.0 Hz, 3H), 3.74 (s, 3H), 3.30 (q, J = 8.8 Hz, 2H), 2.41 (s, 2H), 2.21 – 2.10 (m, 1H), 1.94 (dt, J = 14.1, 5.3 Hz, 1H), 1.88 – 1.73 (m, 3H), 1.70 – 1.63 (m, 1H), 0.99 (dd, J = 6.2, 3.2 Hz, 6H).

N-((S)-1-(((S)-1-amino-1-oxo-3-((S)-2-oxopyrrolidin-3-yl)propan-2-yl)amino)-4-methyl-1-oxopentan-2-yl)-4-methoxy-1H-indole-2-carboxamide (**26**)

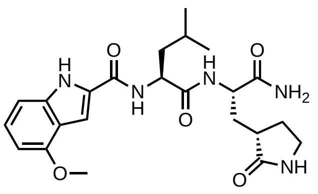

Compound **25** (93.5 mg, 0.20 mmol) was dissolved in anhydrous MeOH (0.373 mL). 7M ammonia (NH_3) in MeOH (7.84 mL) was then added in 3 portions (4:1:1) over three days. The reaction mixture was stirred at room temperature for 3 days. The crude product (92.2 mg, ~100%) was yielded after removing the solvent by rotavapor or under high vacuum pressure.

^{1}H NMR (400 MHz, cd$_3$od) δ 7.29 (s, 1H), 7.13 (t, J = 8.0 Hz, 1H), 7.02 (d, J = 8.3 Hz, 1H), 6.49 (d, J = 7.7 Hz, 1H), 4.60 (dd, J = 9.6, 4.9 Hz, 1H), 4.46 (dd, J = 11.2, 4.3 Hz, 1H), 3.91 (s, 3H), 3.27 – 3.18 (m, 2H), 2.51 (qd, J = 9.5, 4.5 Hz, 1H), 2.28 (dddd, J = 11.7, 8.8, 6.9, 2.8 Hz, 1H), 2.19 – 2.09 (m, 1H), 1.79 (ddd, J = 14.8, 10.4, 4.1 Hz, 5H), 0.98 (dd, J = 13.5, 5.9 Hz, 6H).

4-methoxy-N-((S)-4-methyl-1-oxo-1-(((S)-1-((S)-2-oxopyrrolidin-3-yl)but-3-yn-2-yl)amino)pentan-2-yl)-1H-indole-2-carboxamide (**3d**)

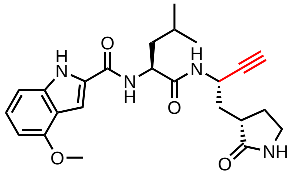

Similar to the synthetic procedure for compound **3**. Yielded white solid (7.5 mg, 24%).

^{1}H NMR (600 MHz, acetone) δ 10.76 (s, 1H), 7.89 (d, *J* = 8.5 Hz, 1H), 7.76 (d, *J* = 8.3 Hz, 1H), 7.27 (d, *J* = 2.3 Hz, 1H), 7.16 – 7.09 (m, 2H), 6.73 (s, 1H), 6.52 (dd, *J* = 6.8, 1.6 Hz, 1H), 4.88 (dtt, *J* = 12.4, 5.1, 2.4 Hz, 1H), 4.69 – 4.63 (m, 1H), 3.91 (d, *J* = 16.8 Hz, 3H), 3.26 – 3.15 (m, 2H), 2.71 (t, *J* = 2.6 Hz, 1H), 2.43 (qd, *J* = 9.3, 4.8 Hz, 1H), 2.30 (dddd, *J* = 12.5, 8.7, 6.9, 2.3 Hz, 1H), 2.19 (ddd, *J* = 14.5, 10.0, 4.9 Hz, 1H), 1.83 – 1.71 (m, 4H), 1.65 (ddd, *J* = 14.2, 9.3, 5.4 Hz, 1H), 0.96 (d, *J* = 6.6 Hz, 3H), 0.93 (d, *J* = 6.5 Hz, 3H).

N-((S)-1-(((S)-1-cyano-2-((S)-2-oxopyrrolidin-3-yl)ethyl)amino)-4-methyl-1-oxopentan-2-yl)-4-methoxy-1H-indole-2-carboxamide (**3g**)

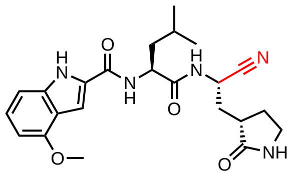

Similar to the synthetic procedure for compound **15**. Yielded white solid (18.2 mg, 22%).

^{1}H NMR (600 MHz, acetone) δ 10.82 (s, 1H), 8.53 (d, *J* = 7.7 Hz, 1H), 7.88 (d, *J* = 8.1 Hz, 1H), 7.28 (d, *J* = 2.4 Hz, 1H), 7.17 – 7.08 (m, 2H), 6.98 (s, 1H), 6.52 (dd, *J* = 7.1, 1.2 Hz, 1H), 5.09 (ddd, *J* = 10.1, 7.7, 6.2 Hz, 1H), 4.73 – 4.65 (m, 1H), 3.91 (s, 3H), 3.31 – 3.19 (m, 2H), 2.52 (dtd, *J* = 10.2, 8.2, 6.1 Hz, 1H), 2.34 – 2.23 (m, 2H), 1.94 (ddd, *J* = 14.1, 8.2, 6.3 Hz, 1H), 1.86 – 1.73 (m, 4H), 0.96 (d, *J* = 6.1 Hz, 3H), 0.94 (d, *J* = 6.2 Hz, 3H).

4-methoxy-N-((S)-4-methyl-1-oxo-1-(((S)-1-oxo-3-((S)-2-oxopyrrolidin-3-yl)propan-2-yl)amino)pentan-2-yl)-1H-indole-2-carboxamide (**3e**)

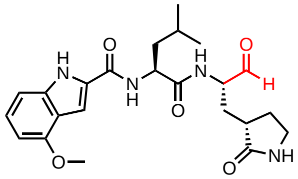

Compound **25** (140 mg, 0.3 mmol) was dissolved in dry DCM (19.2 mL). The reaction flask was charged with N_2 balloon. At 0ºC under N_2 atmosphere, lithium borohydride (2M in THF, 0.3 mL, 0.6 mmol) was added slowly to the reaction vessel. The reaction was stirred at 0ºC for 3 hours. Upon completion, the reaction was quenched with saturated ammonium chloride (NH_4Cl) and extracted with $NaHCO_3$ and brine. The crude product (105.6 mg, 80%) was obtained after removing organic solvent and used in the next step without further purification.

^{1}H NMR (600 MHz, acetone) δ 10.97 (s, 1H), 9.63 – 9.43 (m, 1H), 8.55 – 8.42 (m, 1H), 8.08 – 7.90 (m, 1H), 7.38 – 7.29 (m, 1H), 7.21 – 7.01 (m, 3H), 6.49 (th, *J* = 11.3, 3.4 Hz, 1H), 4.88 – 4.74 (m, 1H), 4.54 – 4.29 (m, 1H), 3.89 (dd, *J* = 8.8, 2.9 Hz, 3H), 3.30 – 3.17 (m, 2H), 2.54 – 2.40 (m, 1H), 2.35 – 2.16 (m, 2H), 1.88 – 1.71 (m, 5H), 0.95 (tq, *J* = 12.1, 7.6 Hz, 6H).

Scheme S11. Synthesis of Compounds 4d-h

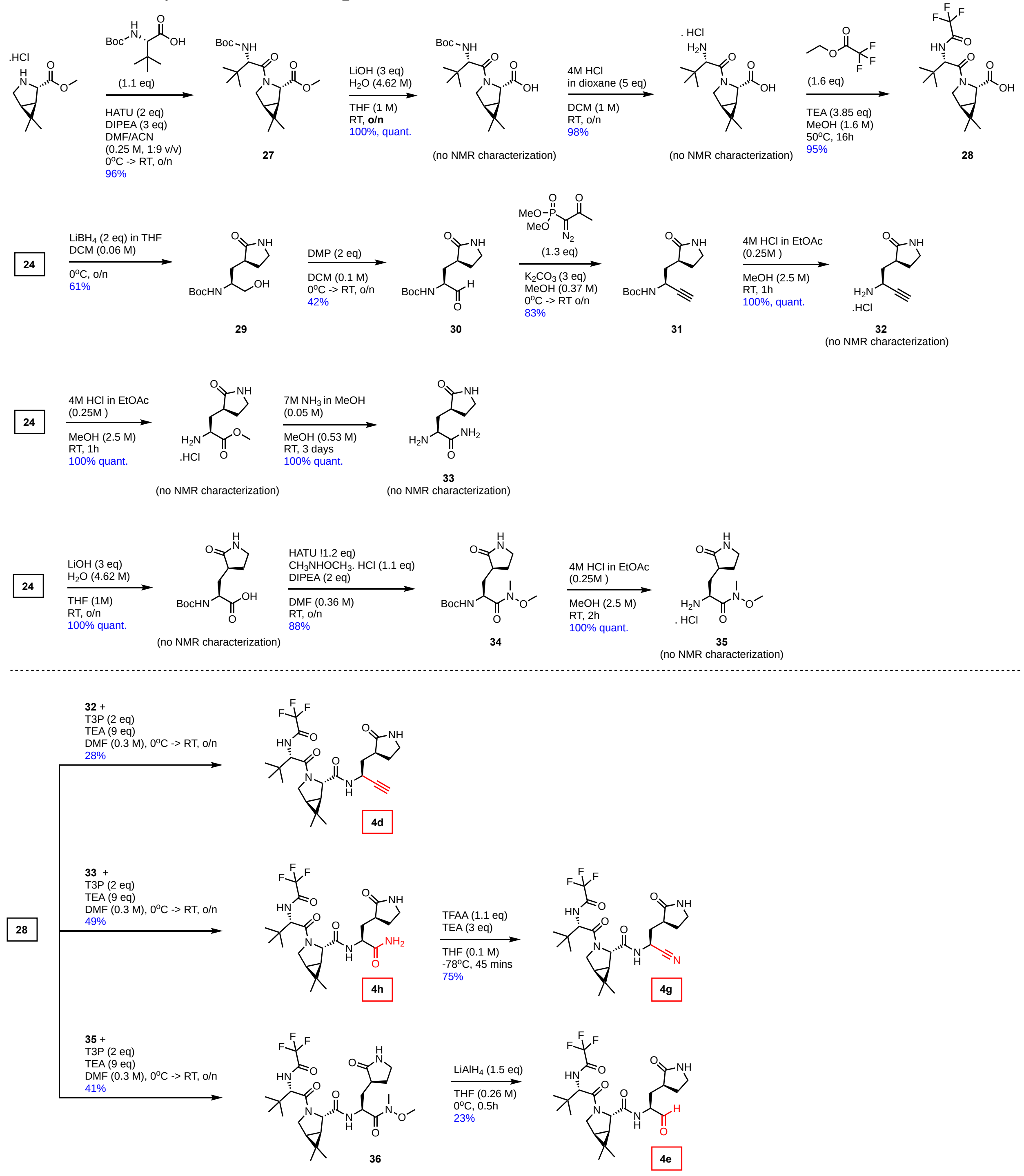

methyl (1R,2S,5S)-3-((S)-2-((tert-butoxycarbonyl)amino)-3,3-dimethylbutanoyl)-6,6-dimethyl-3-azabicyclo[3.1.0]hexane-2-carboxylate (**27**)

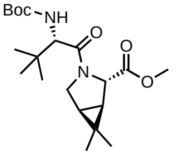

In a dried flask, (1R,2S,5S)-Methyl 6,6-dimethyl-3-azabicyclo[3.1.0]hexane-2-carboxylate hydrochloride (0.5 g, 2.43 mmol) and Boc-Tle-OH (0.6185 g, 2.67 mmol) were dissolved in DMF/ACN (1:9, v/v, 9.72 mL). At 0ºC, amide coupling reagent HATU (3.7 g, 4.86 mmol)was added following by dropwise addition of DIPEA (1.40 mL, 8.02 mmol). The reaction mixture was stirred at 0ºC for 3-5 minutes and then at room temperature overnight. After completion, the reaction vessel undergone solvent removal, extraction with water, $NaHCO_3$, and brine. The collected organic crude mixture was dried over Na_2SO_4 and purified using flash chromatography to yield compound **27** (0.8963 g, 96%)

1H NMR (400 MHz, cdcl3) δ 5.11 (d, *J* = 10.3 Hz, 1H), 4.47 (s, 1H), 4.21 (d, *J* = 10.3 Hz, 1H), 3.99 (d, *J* = 10.3 Hz, 1H), 3.90 – 3.83 (m, 1H), 3.75 (s, 3H), 1.46 – 1.37 (m, 11H), 1.03 (d, *J* = 6.1 Hz, 12H), 0.90 (s, 3H).

(1R,2S,5S)-3-((S)-3,3-dimethyl-2-(2,2,2-trifluoroacetamido)butanoyl)-6,6-dimethyl-3-azabicyclo[3.1.0]hexane-2-carboxylic acid (**28**)

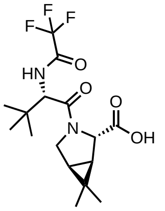

The starting material (672 mg, 2.2 mmol) was dissolved in anhydrous MeOH (2.2 mL). Under N_2 atmosphere, TEA (1.19 mL, 8.5 mmol) was added, generating a pale yellow cloudy reaction mixture. At 0ºC, ethyl trifluoroacetate (0.419 mL, 3.53 mmol) was added drop-wisely. The reaction was then heated up to 50ºC and stirred overnight. Upon completion, the reaction was acidified with 1M HCl to neutralize the acid product. The crude mixture was then extracted with water and brine, dried over Na_2SO_4, and purified using flash chromatography. The product as white solid (760 mg, 95%) was yielded.

^{1}H NMR (600 MHz, cdcl$_3$) δ 9.18 (s, 1H), 7.33 (d, *J* = 9.5 Hz, 1H), 4.59 (d, *J* = 9.5 Hz, 1H), 4.47 (s, 1H), 3.92 (dd, *J* = 10.4, 5.4 Hz, 1H), 3.86 (d, *J* = 10.4 Hz, 1H), 1.61 (d, *J* = 7.5 Hz, 1H), 1.52 (dd, *J* = 7.6, 5.3 Hz, 1H), 1.05 (d, *J* = 12.5 Hz, 12H), 0.88 (s, 3H).

tert-butyl ((S)-1-hydroxy-3-((S)-2-oxopyrrolidin-3-yl)propan-2-yl)carbamate (**29**)

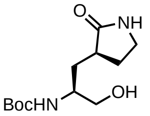

Similar to the synthetic procedure for compound **3e**. Yielded white solid (178.6 mg, 61%).

^{1}H NMR (600 MHz, acetone) δ 6.68 (s, 1H), 5.99 (d, *J* = 8.2 Hz, 1H), 3.90 (t, *J* = 5.9 Hz, 1H), 3.69 – 3.62 (m, 1H), 3.59 – 3.52 (m, 1H), 3.50 – 3.43 (m, 1H), 3.32 – 3.22 (m, 2H), 2.40 – 2.29 (m, 2H), 1.87 (ddd, *J* = 14.0, 10.6, 4.4 Hz, 1H), 1.75 (dq, *J* = 11.2, 8.8 Hz, 1H), 1.53 (ddd, *J* = 13.5, 8.7, 3.9 Hz, 1H), 1.39 (s, 9H).

tert-butyl ((S)-1-oxo-3-((S)-2-oxopyrrolidin-3-yl)propan-2-yl)carbamate (**30**)

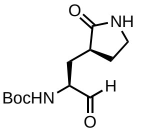

Similar to the synthetic procedure for compound **13**. Yielded white solid (233.3 mg, 42%).

^{1}H NMR (400 MHz, cdcl$_3$) δ 9.50 (s, 1H), 7.01 (s, 1H), 6.16 (d, *J* = 7.0 Hz, 1H), 4.11 (dq, *J* = 9.2, 5.1 Hz, 1H), 3.36 – 3.17 (m, 3H), 2.45 – 2.31 (m, 2H), 1.92 (dd, *J* = 9.1, 5.9 Hz, 1H), 1.88 – 1.81 (m, 1H), 1.38 (s, 9H).

tert-butyl ((S)-1-((S)-2-oxopyrrolidin-3-yl)but-3-yn-2-yl)carbamate (**31**)

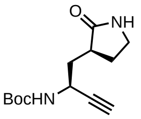

Similar to the synthetic procedure for compound **10**. Yielded white solid (148.4 mg, 83%).

^{1}H NMR (600 MHz, acetone) δ 6.84 – 6.58 (m, 1H), 6.46 (s, 1H), 4.64 – 4.45 (m, 1H), 3.36 – 3.21 (m, 2H), 2.72 (dd, *J* = 14.9, 2.3 Hz, 1H), 2.42 – 2.28 (m, 2H), 2.17 – 2.11 (m, 1H), 1.85 – 1.76 (m, 1H), 1.71 – 1.61 (m, 1H), 1.40 (s, 9H).

(1R,2S,5S)-3-((S)-3,3-dimethyl-2-(2,2,2-trifluoroacetamido)butanoyl)-N-((S)-1-(methoxy(methyl)amino)-1-oxo-3-((S)-2-oxopyrrolidin-3-yl)propan-2-yl)-6,6-dimethyl-3-azabicyclo[3.1.0]hexane-2-carboxamide (**36**)

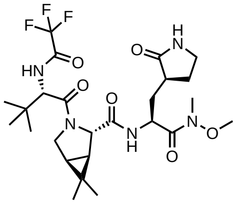

Similar to the synthetic procedure for compound **2**. Yielded white solid (247.2 mg, 41%).

^{1}H NMR (400 MHz, cd$_3$od) δ 4.97 (d, *J* = 11.5 Hz, 1H), 4.57 (s, 1H), 4.36 (d, *J* = 7.2 Hz, 1H), 4.00 (dd, *J* = 10.3, 5.5 Hz, 1H), 3.81 (d, *J* = 10.5 Hz, 5H), 3.33 (s, 1H), 3.29 – 3.25 (m, 1H), 3.19 (d, *J* = 10.8 Hz, 3H), 2.67 (t, *J* = 10.8 Hz, 1H), 2.43 – 2.33 (m, 1H), 2.19 – 2.06 (m, 1H), 1.88 – 1.69 (m, 2H), 1.64 (ddd, *J* = 14.2, 11.2, 3.2 Hz, 1H), 1.57 (dd, *J* = 7.7, 5.3 Hz, 1H), 1.48 (d, *J* = 7.6 Hz, 1H), 1.37 (d, *J* = 6.5 Hz, 1H), 1.06 (d, *J* = 4.2 Hz, 12H), 0.96 (s, 3H).

(1R,2S,5S)-3-((S)-3,3-dimethyl-2-(2,2,2-trifluoroacetamido)butanoyl)-6,6-dimethyl-N-((S)-1-((S)-2-oxopyrrolidin-3-yl)but-3-yn-2-yl)-3-azabicyclo[3.1.0]hexane-2-carboxamide (**4d**)

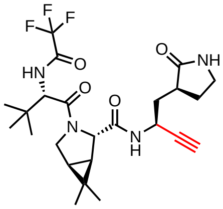

Similar to the synthetic procedure for compound **2**. Yielded white solid (49 mg, 28%).

^{1}H NMR (400 MHz, acetone) δ 8.16 (d, *J* = 9.1 Hz, 1H), 7.79 (d, *J* = 8.9 Hz, 1H), 6.84 (s, 1H), 4.88 (dddd, *J* = 11.2, 8.8, 4.2, 2.3 Hz, 1H), 4.60 (d, *J* = 9.1 Hz, 1H), 4.29 (s, 1H), 4.04 – 4.00 (m, 1H), 3.83 (d, *J* = 10.2 Hz, 1H), 3.30 – 3.14 (m, 2H), 2.77 (d, *J* = 2.4 Hz, 1H), 2.57 – 2.47 (m, 1H), 2.40 – 2.31 (m, 1H), 2.23 (ddd, *J* = 13.9, 11.3, 4.0 Hz, 1H), 1.81 – 1.72 (m, 1H), 1.68 – 1.55 (m, 2H), 1.39 (d, *J* = 7.7 Hz, 1H), 1.07 (s, 9H), 1.05 (s, 3H), 0.88 (s, 3H).

(1R,2S,5S)-3-((S)-3,3-dimethyl-2-(2,2,2-trifluoroacetamido)butanoyl)-6,6-dimethyl-N-((S)-1-oxo-3-((S)-2-oxopyrrolidin-3-yl)propan-2-yl)-3-azabicyclo[3.1.0]hexane-2-carboxamide (**4e**)

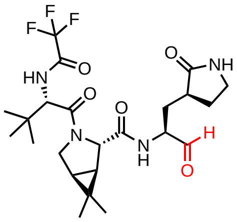

Similar to the synthetic procedure for compound **13**. Yielded white solid (32.5 mg, 23%)

^{1}H NMR (400 MHz, acetone) δ 9.55 (d, *J* = 4.0 Hz, 1H), 8.29 – 8.05 (m, 2H), 6.87 (s, 1H), 4.61 (d, *J* = 9.1 Hz, 1H), 4.42 (ddd, *J* = 13.9, 6.9, 3.2 Hz, 1H), 4.36 (s, 1H), 4.09 – 4.02 (m, 1H), 3.87 – 3.77 (m, 1H), 3.32 – 3.23 (m, 2H), 2.60 – 2.46 (m, 1H), 2.43 – 2.28 (m, 1H), 1.94 – 1.86 (m, 1H), 1.86 – 1.76 (m, 2H), 1.57 (dd, *J* = 7.7, 5.3 Hz, 1H), 1.49 (d, *J* = 7.7 Hz, 1H), 1.06 (d, *J* = 5.5 Hz, 12H), 0.89 (s, 3H).

(1R,2S,5S)-N-((S)-1-cyano-2-((S)-2-oxopyrrolidin-3-yl)ethyl)-3-((S)-3,3-dimethyl-2-(2,2,2-trifluoroacetamido)butanoyl)-6,6-dimethyl-3-azabicyclo[3.1.0]hexane-2-carboxamide (**4g**)

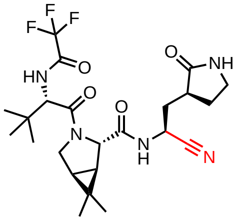

Similar to the synthetic procedure for compound **2** and **15**. Yielded white solid (40 mg, 76%).

^{1}H NMR (500 MHz, acetone) δ 8.36 (d, *J* = 8.1 Hz, 1H), 8.16 (d, *J* = 9.0 Hz, 1H), 6.93 (s, 1H), 5.14 – 5.06 (m, 1H), 4.61 (d, *J* = 9.0 Hz, 1H), 4.29 (s, 1H), 4.08 – 4.04 (m, 1H), 3.85 (d, *J* = 10.2 Hz, 1H), 3.35 – 3.23 (m, 2H), 3.00 – 2.85 (m, 1H), 2.57 (dtd, *J* = 10.4, 8.8, 5.4 Hz, 1H), 2.39 – 2.27 (m, 2H), 1.97 – 1.82 (m, 2H), 1.62 (dd, *J* = 7.6, 5.4 Hz, 1H), 1.45 (d, *J* = 7.6 Hz, 1H), 1.08 (d, *J* = 4.3 Hz, 12H), 0.90 (s, 3H).

(1R,2S,5S)-N-((S)-1-amino-1-oxo-3-((S)-2-oxopyrrolidin-3-yl)propan-2-yl)-3-((S)-3,3-dimethyl-2-(2,2,2-trifluoroacetamido)butanoyl)-6,6-dimethyl-3-azabicyclo[3.1.0]hexane-2-carboxamide (**4h**)

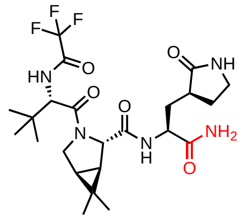

Similar to the synthetic procedure for compound **2**. Yielded white solid (92.1 mg, 9%).

^{1}H NMR (400 MHz, acetone) δ 8.45 (d, *J* = 9.0 Hz, 1H), 8.14 (d, *J* = 8.1 Hz, 1H), 7.24 (d, *J* = 7.2 Hz, 2H), 6.81 (s, 1H), 5.61 (s, 1H), 4.64 (d, *J* = 9.1 Hz, 1H), 4.57 (ddd, *J* = 15.6, 7.8, 3.2 Hz, 1H), 4.39 (s, 1H), 4.13 – 4.07 (m, 1H), 3.82 (d, *J* = 10.4 Hz, 1H), 3.27 (ddd, *J* = 16.6, 9.3, 7.0 Hz, 2H), 2.56 (qd, *J* = 9.3, 4.9 Hz, 1H), 2.39 – 2.29 (m, 1H), 2.11 (ddd, *J* = 13.8, 11.3, 5.0 Hz, 1H), 1.78 (ddd, *J* = 13.3, 9.3, 4.3 Hz, 2H), 1.53 (dq, *J* = 12.1, 6.2 Hz, 2H), 1.08 (s, 9H), 1.04 (s, 3H), 0.88 (s, 3H).

Scheme S12. Synthesis of Compound 4i

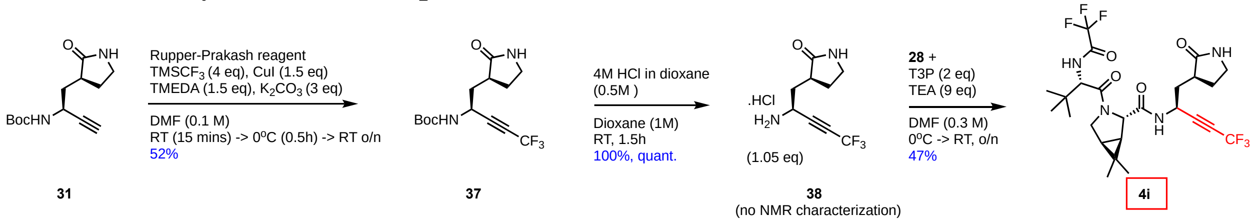

tert-butyl ((S)-5,5,5-trifluoro-1-((S)-2-oxopyrrolidin-3-yl)pent-3-yn-2-yl)carbamate (**37**)

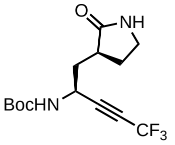

The dried reaction flask was charged with CuI (65.9 mg, 0.48 mmol), K_2CO_3 (131.8 mg, 0.95 mmol), TMEDA (55.4 mg, 0.48 mmol) in anhydrous DMF (1.51 mL). The reaction was stirred under air at room temperature for 15 minutes. Rupper-Prakash reagent, $TMSCF_3$, (89 µL, 0.635 mmol) was then added, and the reaction was stirred under air for another 5 mintues. In the meantime, the vessel containing compound **31** and $TMSCF_3$ (89 µL, 0.635 mmol) dissolved in DMF (1.51 mL) was pre-cooled to 0°C . This mixture was added to the initial flask slowly at 0°C for 10-15 minutes. The reaction mixture was then stirred at 0°C for another 30 minutes under air atmosphere followed by overnight stirring at room temperature. After completion, the reaction mixture was washed with water, brine and undergone the general workup procedure. The flash chromatography was done to yield the final product as white solid (85.5 mg, 84%)

^{1}H NMR (400 MHz, cdcl$_3$) δ 6.61 (s, 1H), 5.59 – 5.31 (m, 1H), 4.78 – 4.54 (m, 1H), 3.33 (t, J = 8.0 Hz, 2H), 2.42 (d, J = 17.3 Hz, 2H), 2.25 (ddd, J = 14.5, 9.6, 4.5 Hz, 1H), 1.89 – 1.71 (m, 2H), 1.44 (s, 9H).

(1R,2S,5S)-3-((S)-3,3-dimethyl-2-(2,2,2-trifluoroacetamido)butanoyl)-6,6-dimethyl-N-((S)-5,5,5-trifluoro-1-((S)-2-oxopyrrolidin-3-yl)pent-3-yn-2-yl)-3-azabicyclo[3.1.0]hexane-2-carboxamide (**4i**)

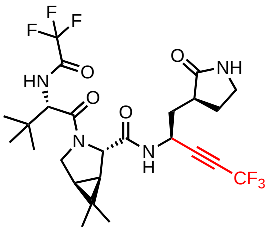

Similar to the synthetic procedure for compound **2**. Yielded white solid (34 mg, 47%).

^{1}H NMR (400 MHz, acetone) δ 8.17 (dd, J = 14.8, 9.3 Hz, 2H), 7.88 (s, 1H), 6.89 (s, 1H), 5.19 – 5.11 (m, 1H), 4.64 (d, J = 9.0 Hz, 1H), 4.32 (d, J = 6.7 Hz, 1H), 3.92 – 3.81 (m, 1H), 3.43 – 3.22 (m, 3H), 2.64 – 2.54 (m, 1H), 2.43 – 2.26 (m, 2H), 1.91 – 1.79 (m, 2H), 1.63 (dd, J = 7.8, 5.6 Hz, 1H), 1.46 (d, J = 3.4 Hz, 1H), 1.11 (d, J = 3.1 Hz, 9H), 1.08 (d, J = 5.3 Hz, 3H), 0.94 – 0.89 (m, 3H).

Scheme S13. Synthesis of clickable probes Alk-4d & Alk-4i

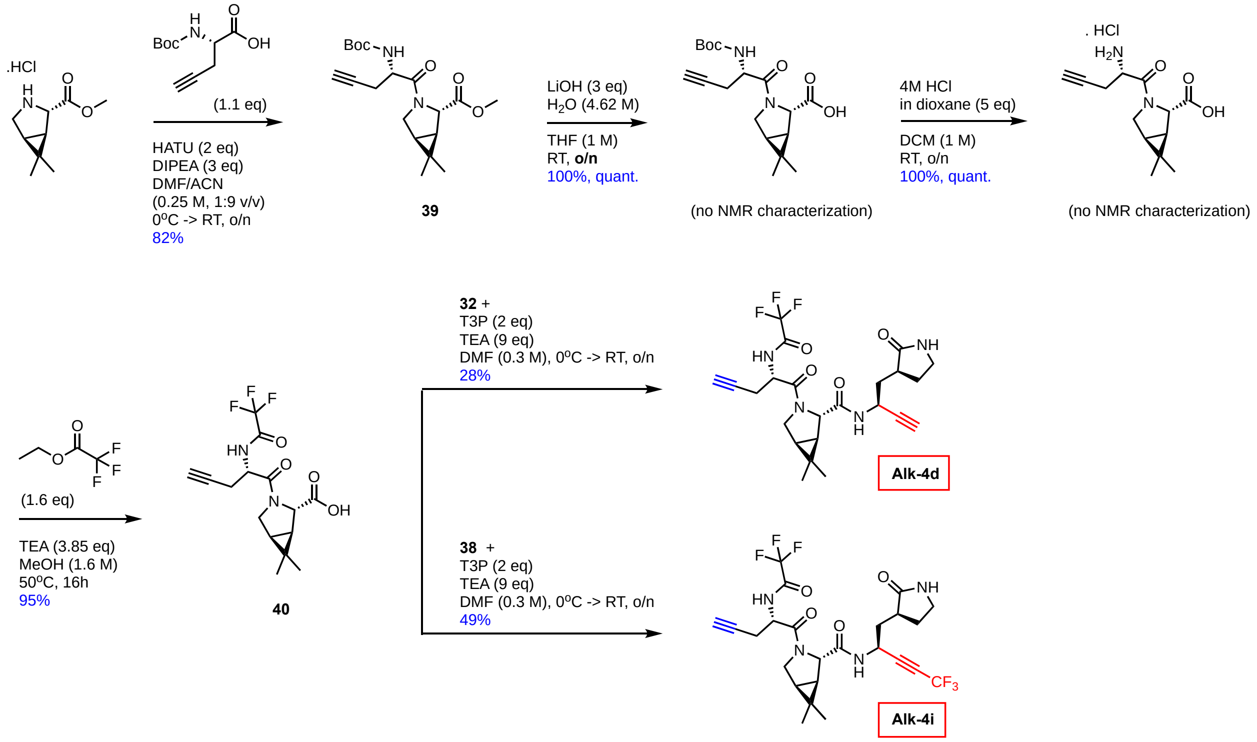

methyl (1R,2S,5S)-3-((S)-2-((tert-butoxycarbonyl)amino)pent-4-ynoyl)-6,6-dimethyl-3-azabicyclo[3.1.0]hexane-2-carboxylate (**39**)

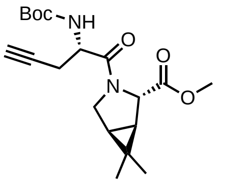

Similar to the synthetic procedure for compound **27**. Yielded white solid (730 mg, 82%).

^{1}H NMR (400 MHz, cdcl$_3$) δ 5.22 (d, J = 9.2 Hz, 1H), 4.58 (dt, J = 9.1, 6.5 Hz, 1H), 4.44 (s, 1H), 3.93 (dd, J = 10.1, 4.9 Hz, 1H), 3.86 (d, J = 10.2 Hz, 1H), 3.78 (s, 1H), 3.74 (s, 3H), 2.70 – 2.61 (m, 1H), 2.57 – 2.49 (m, 1H), 2.04 (d, J = 2.7 Hz, 1H), 1.41 (s, 13H), 1.05 (s, 3H).

(1R,2S,5S)-6,6-dimethyl-N-((S)-1-((S)-2-oxopyrrolidin-3-yl)but-3-yn-2-yl)-3-((S)-2-(2,2,2-trifluoroacetamido)pent-4-ynoyl)-3-azabicyclo[3.1.0]hexane-2-carboxamide (**Alk-4d**)

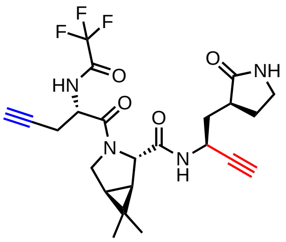

Similar to the synthetic procedure for compound **2**. Yielded white solid (121 mg, 57%).

^{1}H NMR (400 MHz, acetone) δ 8.97 (d, J = 8.0 Hz, 1H), 7.80 (d, J = 8.6 Hz, 1H), 7.08 (s, 1H), 4.92 – 4.83 (m, 2H), 4.32 (s, 1H), 4.12 – 4.05 (m, 1H), 3.91 (d, J = 10.3 Hz, 1H), 3.30 (dtd, J = 16.0, 8.6, 4.1 Hz, 2H), 2.82 – 2.71 (m, 3H), 2.60 (t, J = 2.6 Hz, 1H), 2.52 (ddd, J = 12.4, 6.7, 3.4 Hz, 1H), 2.38 (tdd, J = 11.1, 7.5, 2.3 Hz, 1H), 2.24 (ddd, J = 13.9, 10.7, 4.5 Hz, 1H), 1.84 – 1.76 (m, 1H), 1.70 (ddd, J = 14.5, 10.0, 4.9 Hz, 1H), 1.61 (dd, J = 7.6, 5.1 Hz, 1H), 1.50 (d, J = 7.6 Hz, 1H), 1.10 (s, 3H), 0.97 (s, 3H).

(1R,2S,5S)-6,6-dimethyl-N-((S)-5,5,5-trifluoro-1-((S)-2-oxopyrrolidin-3-yl)pent-3-yn-2-yl)-3-((S)-2-(2,2,2-trifluoroacetamido)pent-4-ynoyl)-3-azabicyclo[3.1.0]hexane-2-carboxamide (**Alk-4i**)

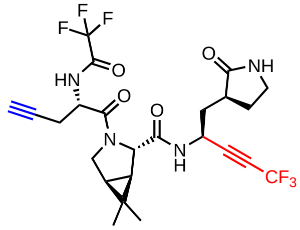

Similar to the synthetic procedure for compound **2**. Yielded white solid (26.4 mg, 49%).

^{1}H NMR (400 MHz, acetone) δ 9.06 (d, *J* = 6.9 Hz, 1H), 8.66 (d, *J* = 9.3 Hz, 1H), 7.16 (d, *J* = 15.1 Hz, 1H), 4.85 (s, 1H), 4.82 – 4.74 (m, 1H), 4.04 (s, 1H), 3.96 (dd, *J* = 10.5, 5.5 Hz, 1H), 3.65 (d, *J* = 10.6 Hz, 1H), 3.28 (q, *J* = 9.6 Hz, 2H), 2.59 (td, *J* = 6.0, 2.9 Hz, 2H), 2.32 (q, *J* = 3.6 Hz, 2H), 1.85 (t, *J* = 4.6 Hz, 1H), 1.77 (t, *J* = 11.2 Hz, 1H), 1.53 – 1.38 (m, 3H), 1.32 (d, *J* = 7.6 Hz, 1H), 0.92 (s, 3H), 0.77 (s, 3H).

3. ^{1}H-NMR Characterization of All Compounds Prepared for This Study

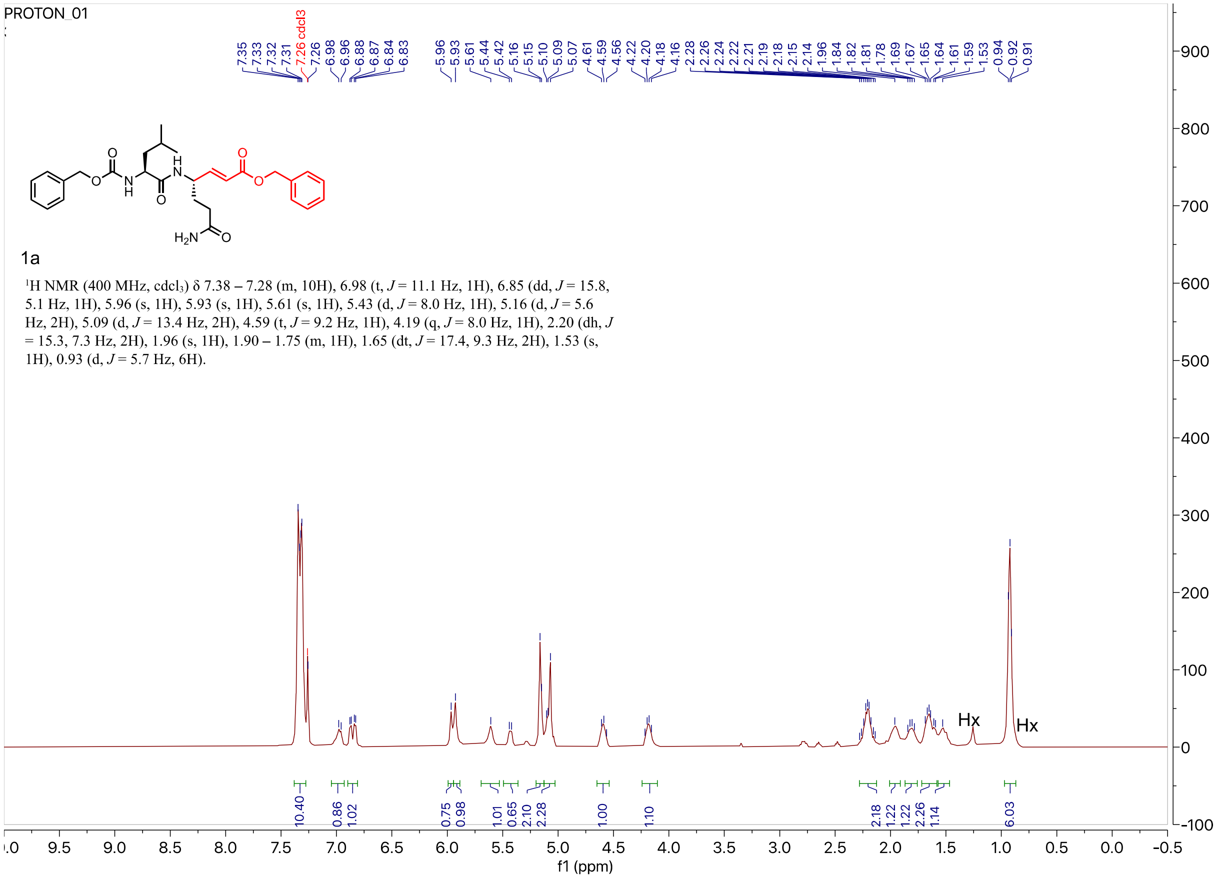

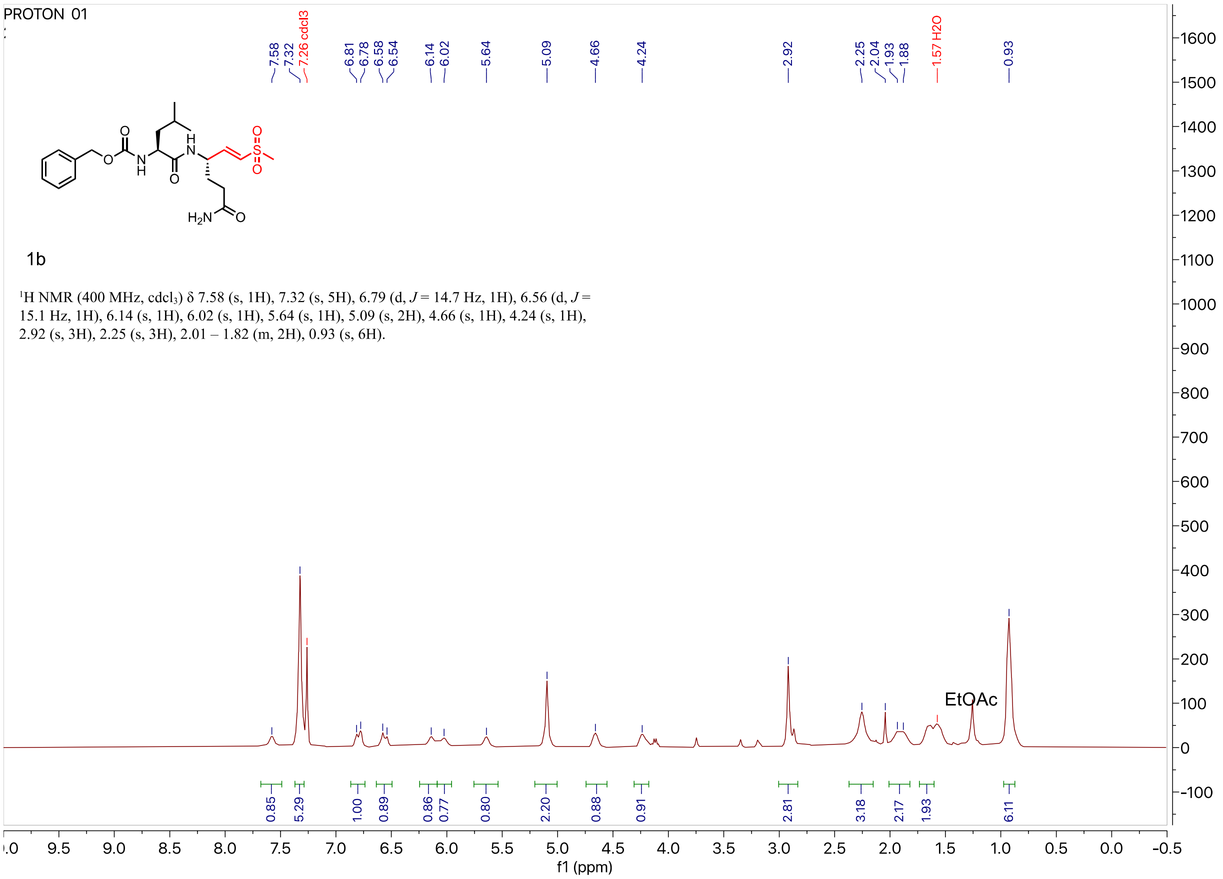

^{1}H NMR (400 MHz, cdcl$_3$) δ 7.58 (s, 1H), 7.32 (s, 5H), 6.79 (d, *J* = 14.7 Hz, 1H), 6.56 (d, *J* = 15.1 Hz, 1H), 6.14 (s, 1H), 6.02 (s, 1H), 5.64 (s, 1H), 5.09 (s, 2H), 4.66 (s, 1H), 4.24 (s, 1H), 2.92 (s, 3H), 2.25 (s, 3H), 2.01 – 1.82 (m, 2H), 0.93 (s, 6H).

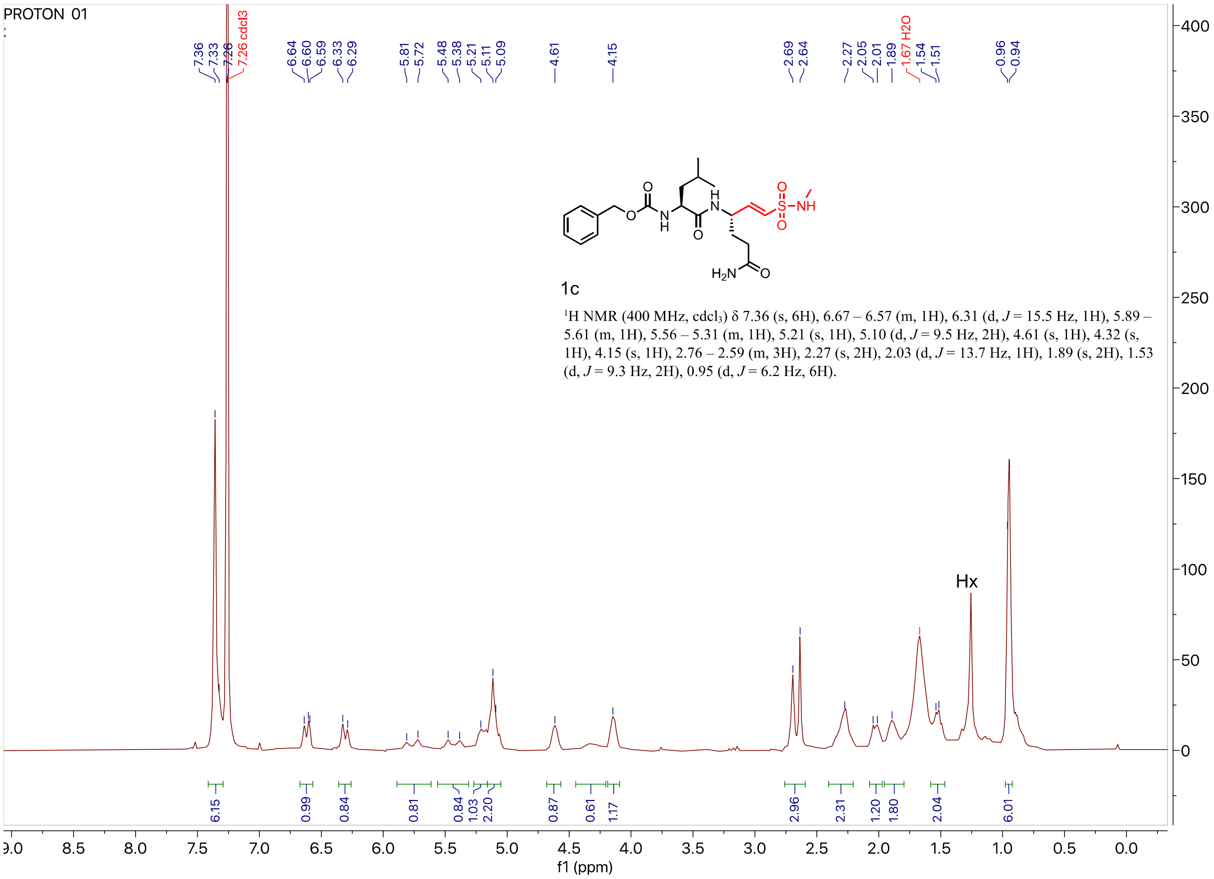
PROTON 01
1c
^{1}H NMR (400 MHz, cdcl$_3$) δ 7.36 (s, 6H), 6.67 – 6.57 (m, 1H), 6.31 (d, J = 15.5 Hz, 1H), 5.89 – 5.61 (m, 1H), 5.56 – 5.31 (m, 1H), 5.21 (s, 1H), 5.10 (d, J = 9.5 Hz, 2H), 4.61 (s, 1H), 4.32 (s, 1H), 4.15 (s, 1H), 2.76 – 2.59 (m, 3H), 2.27 (s, 2H), 2.03 (d, J = 13.7 Hz, 1H), 1.89 (s, 2H), 1.53 (d, J = 9.3 Hz, 2H), 0.95 (d, J = 6.2 Hz, 6H).
Hx
7.26 cdcl3
1.67 H2O
f1 (ppm)

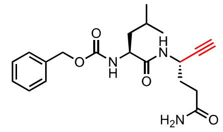

1d

^{1}H NMR (400 MHz, cdcl$_3$) δ 7.34 (d, J = 4.8 Hz, 5H), 7.15 – 6.97 (m, 1H), 6.05 (s, 1H), 5.68 (s, 1H), 5.52 – 5.28 (m, 1H), 5.10 (d, J = 8.2 Hz, 2H), 4.73 (p, J = 7.6 Hz, 1H), 4.18 (q, J = 7.6 Hz, 1H), 2.41 – 2.25 (m, 3H), 2.00 (tt, J = 14.2, 6.7 Hz, 2H), 1.66 (dt, J = 14.6, 7.5 Hz, 2H), 1.57 – 1.48 (m, 1H), 0.93 (d, J = 5.9 Hz, 6H).

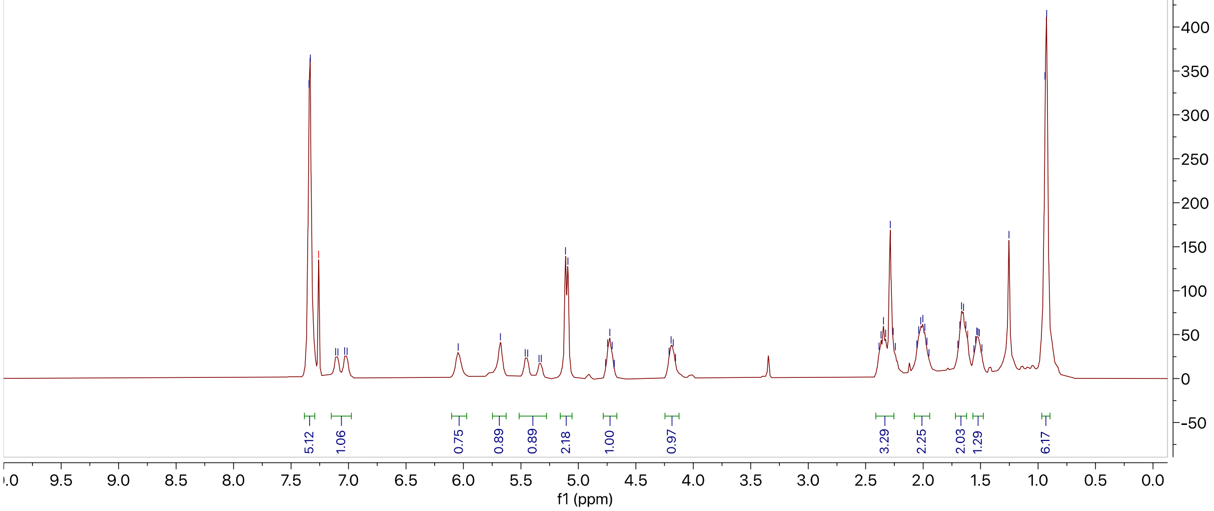

PROTON 01

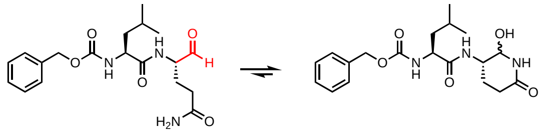

1e

^{1}H NMR (600 MHz, cdcl$_3$) δ 7.37 – 7.26 (m, 6H), 7.15 (s, 1H), 6.90 – 6.63 (m, 1H), 5.53 (s, 1H), 5.07 (q, J = 8.0 Hz, 2H), 4.85 (s, 1H), 4.28 – 4.06 (m, 2H), 2.40 (d, J = 9.1 Hz, 2H), 2.13 – 2.00 (m, 1H), 1.74 (s, 1H), 1.69 – 1.57 (m, 2H), 1.55 – 1.49 (m, 1H), 0.91 (t, J = 7.0 Hz, 6H).

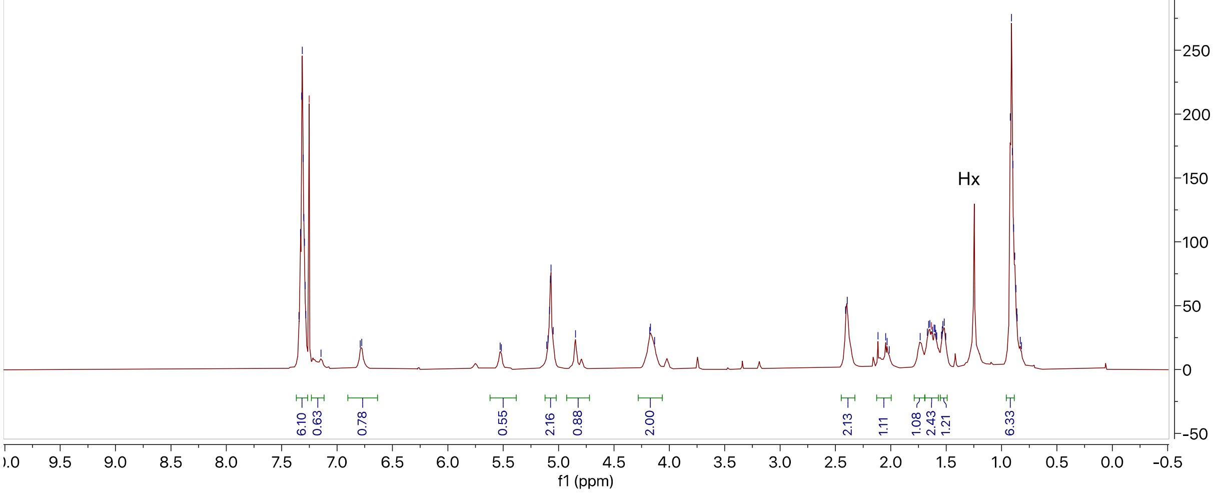

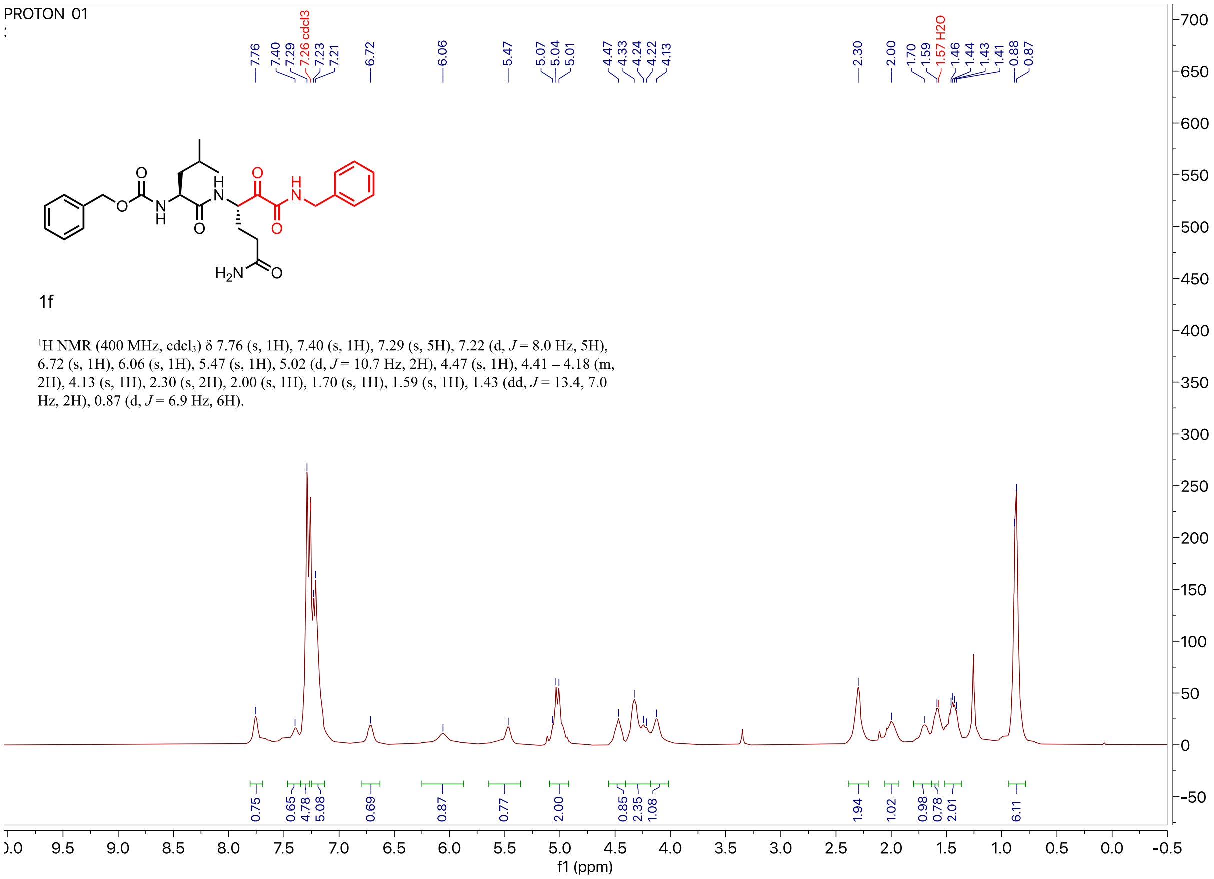

^{1}H NMR (400 MHz, cdcl$_3$) δ 7.76 (s, 1H), 7.40 (s, 1H), 7.29 (s, 5H), 7.22 (d, J = 8.0 Hz, 5H), 6.72 (s, 1H), 6.06 (s, 1H), 5.47 (s, 1H), 5.02 (d, J = 10.7 Hz, 2H), 4.47 (s, 1H), 4.41 – 4.18 (m, 2H), 4.13 (s, 1H), 2.30 (s, 2H), 2.00 (s, 1H), 1.70 (s, 1H), 1.59 (s, 1H), 1.43 (dd, J = 13.4, 7.0 Hz, 2H), 0.87 (d, J = 6.9 Hz, 6H).

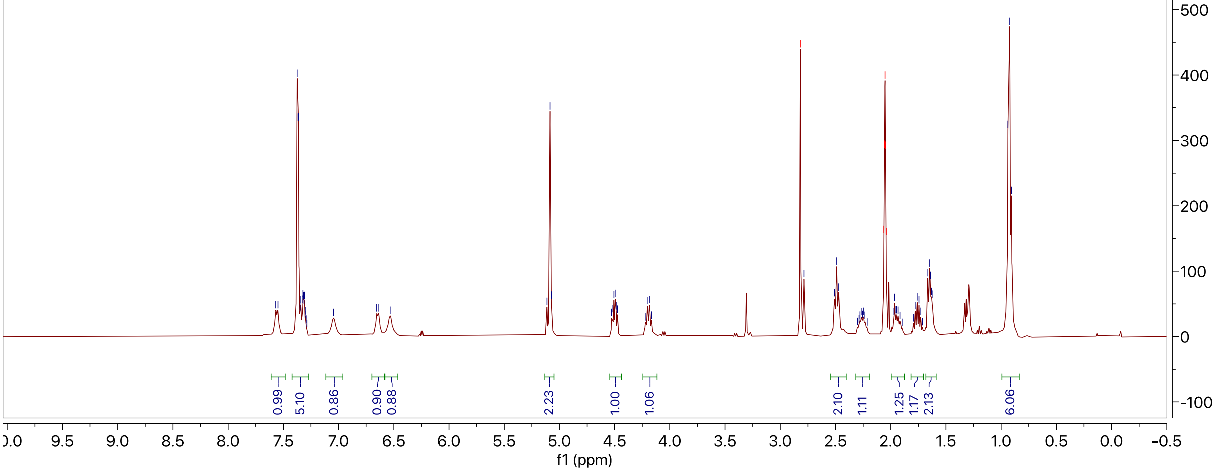

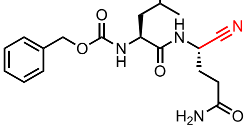

1g

^{1}H NMR (400 MHz, acetone) δ 7.56 (d, *J* = 8.1 Hz, 1H), 7.42 – 7.27 (m, 5H), 7.05 (s, 1H), 6.64 (d, *J* = 7.5 Hz, 1H), 6.53 (s, 1H), 5.08 (s, 2H), 4.50 (td, *J* = 8.5, 4.7 Hz, 1H), 4.20 (q, *J* = 7.5 Hz, 1H), 2.49 (t, *J* = 7.6 Hz, 2H), 2.32 – 2.19 (m, 1H), 2.00 – 1.88 (m, 1H), 1.75 (dq, *J* = 13.1, 6.6 Hz, 1H), 1.65 (dd, *J* = 8.1, 5.3 Hz, 2H), 0.92 (t, *J* = 6.4 Hz, 6H).

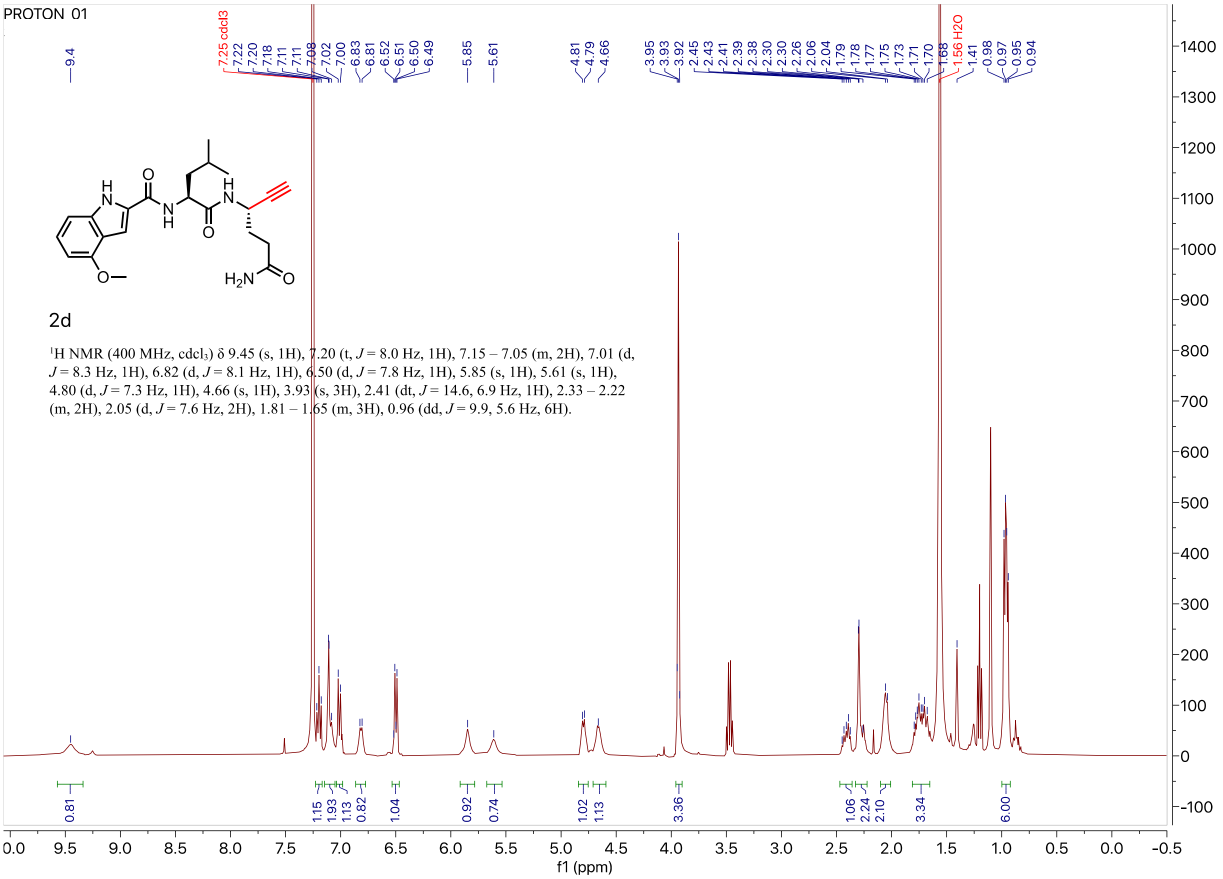

2d

^{1}H NMR (400 MHz, cdcl$_3$) δ 9.45 (s, 1H), 7.20 (t, J = 8.0 Hz, 1H), 7.15 – 7.05 (m, 2H), 7.01 (d, J = 8.3 Hz, 1H), 6.82 (d, J = 8.1 Hz, 1H), 6.50 (d, J = 7.8 Hz, 1H), 5.85 (s, 1H), 5.61 (s, 1H), 4.80 (d, J = 7.3 Hz, 1H), 4.66 (s, 1H), 3.93 (s, 3H), 2.41 (dt, J = 14.6, 6.9 Hz, 1H), 2.33 – 2.22 (m, 2H), 2.05 (d, J = 7.6 Hz, 2H), 1.81 – 1.65 (m, 3H), 0.96 (dd, J = 9.9, 5.6 Hz, 6H).

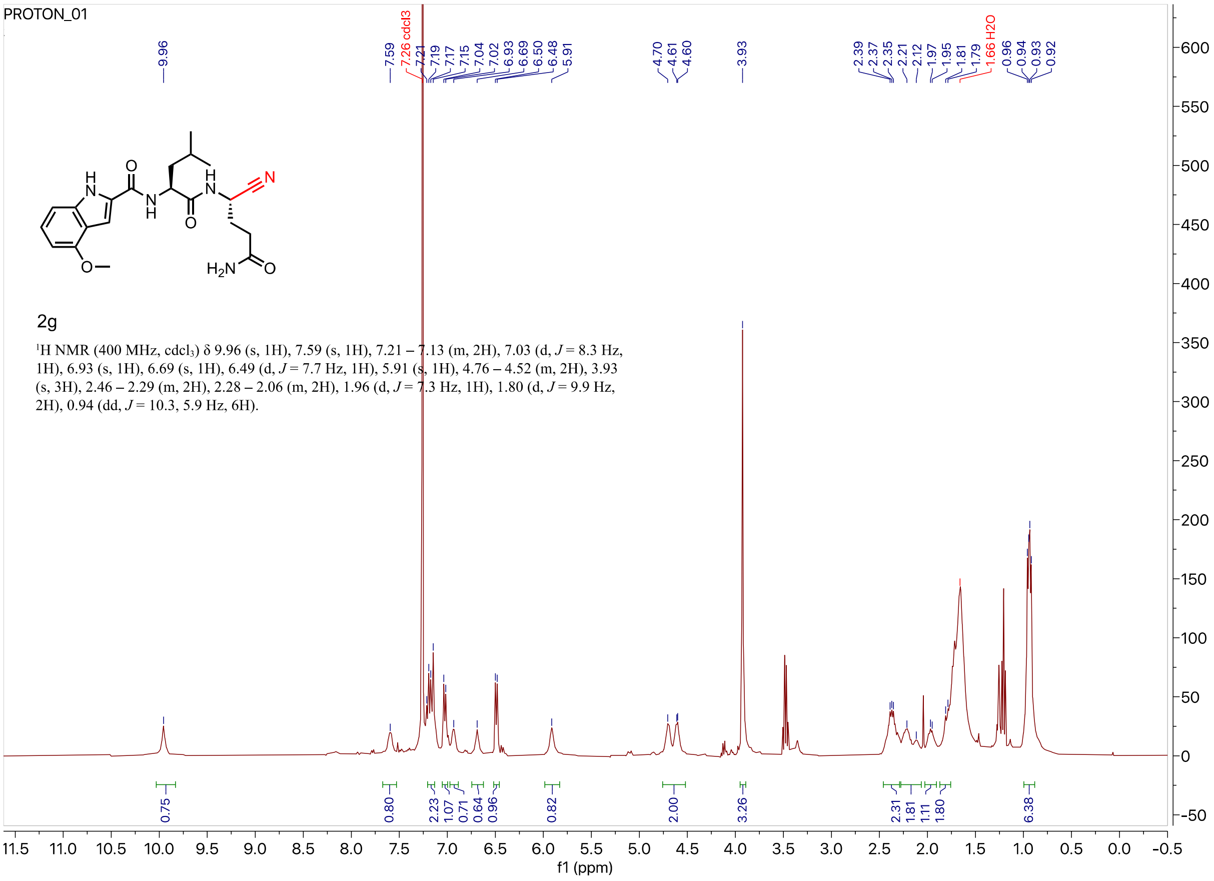
PROTON_01
9.96
7.59
7.26 cdcl3
7.21
7.19
7.17
7.15
7.04
7.02
6.93
6.69
6.50
6.48
5.91
4.70
4.61
4.60
3.93
2.39
2.37
2.35
2.21
2.12
1.97
1.95
1.81
1.79
1.66 H2O
0.96
0.94
0.93
0.92
2g
¹H NMR (400 MHz, cdcl₃) δ 9.96 (s, 1H), 7.59 (s, 1H), 7.21 – 7.13 (m, 2H), 7.03 (d, J = 8.3 Hz, 1H), 6.93 (s, 1H), 6.69 (s, 1H), 6.49 (d, J = 7.7 Hz, 1H), 5.91 (s, 1H), 4.76 – 4.52 (m, 2H), 3.93 (s, 3H), 2.46 – 2.29 (m, 2H), 2.28 – 2.06 (m, 2H), 1.96 (d, J = 7.3 Hz, 1H), 1.80 (d, J = 9.9 Hz, 2H), 0.94 (dd, J = 10.3, 5.9 Hz, 6H).
0.75
0.80
2.23
1.07
0.71
0.64
0.96
0.82
2.00
3.26
2.31
1.81
1.11
1.80
6.38
f1 (ppm)

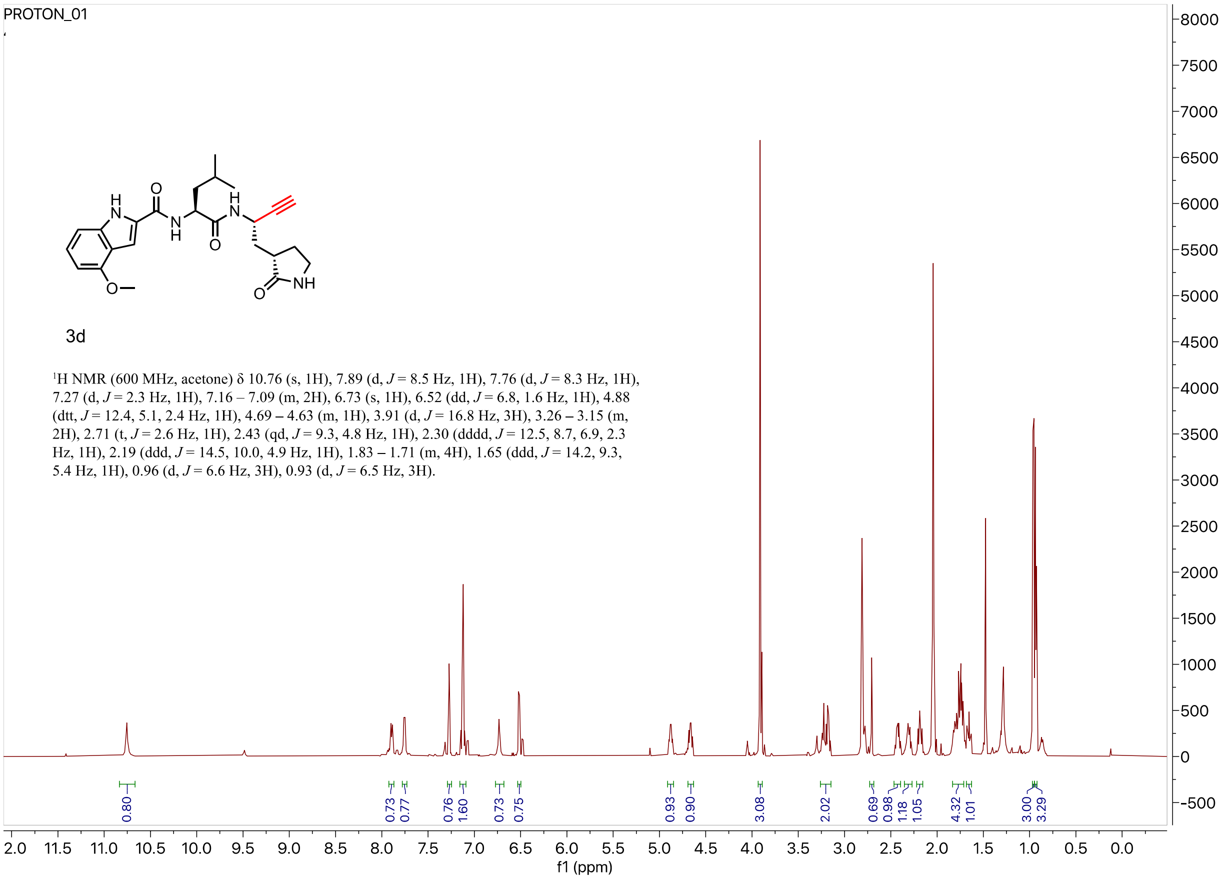

3d

^{1}H NMR (600 MHz, acetone) δ 10.76 (s, 1H), 7.89 (d, *J* = 8.5 Hz, 1H), 7.76 (d, *J* = 8.3 Hz, 1H), 7.27 (d, *J* = 2.3 Hz, 1H), 7.16 – 7.09 (m, 2H), 6.73 (s, 1H), 6.52 (dd, *J* = 6.8, 1.6 Hz, 1H), 4.88 (dtt, *J* = 12.4, 5.1, 2.4 Hz, 1H), 4.69 – 4.63 (m, 1H), 3.91 (d, *J* = 16.8 Hz, 3H), 3.26 – 3.15 (m, 2H), 2.71 (t, *J* = 2.6 Hz, 1H), 2.43 (qd, *J* = 9.3, 4.8 Hz, 1H), 2.30 (dddd, *J* = 12.5, 8.7, 6.9, 2.3 Hz, 1H), 2.19 (ddd, *J* = 14.5, 10.0, 4.9 Hz, 1H), 1.83 – 1.71 (m, 4H), 1.65 (ddd, *J* = 14.2, 9.3, 5.4 Hz, 1H), 0.96 (d, *J* = 6.6 Hz, 3H), 0.93 (d, *J* = 6.5 Hz, 3H).

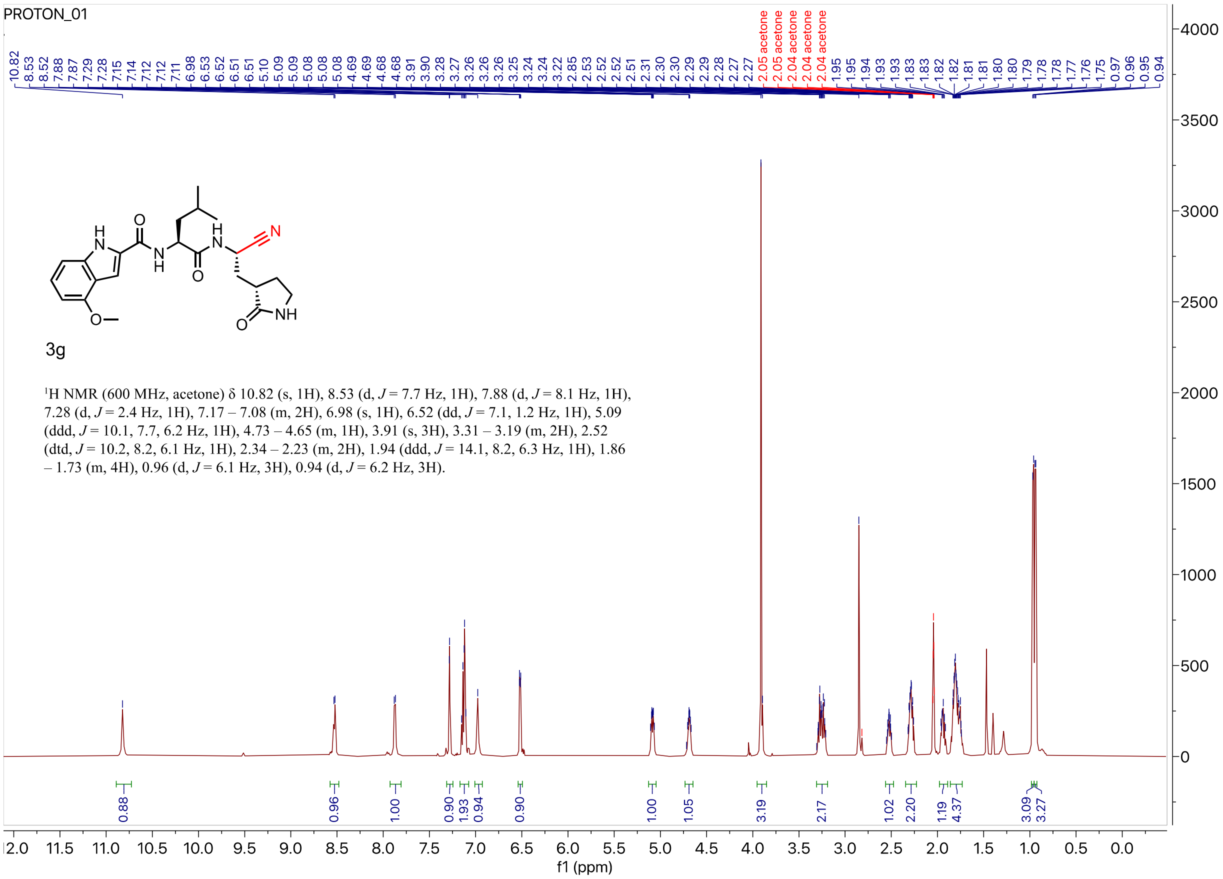

3g

^{1}H NMR (600 MHz, acetone) δ 10.82 (s, 1H), 8.53 (d, *J* = 7.7 Hz, 1H), 7.88 (d, *J* = 8.1 Hz, 1H), 7.28 (d, *J* = 2.4 Hz, 1H), 7.17 – 7.08 (m, 2H), 6.98 (s, 1H), 6.52 (dd, *J* = 7.1, 1.2 Hz, 1H), 5.09 (ddd, *J* = 10.1, 7.7, 6.2 Hz, 1H), 4.73 – 4.65 (m, 1H), 3.91 (s, 3H), 3.31 – 3.19 (m, 2H), 2.52 (dtd, *J* = 10.2, 8.2, 6.1 Hz, 1H), 2.34 – 2.23 (m, 2H), 1.94 (ddd, *J* = 14.1, 8.2, 6.3 Hz, 1H), 1.86 – 1.73 (m, 4H), 0.96 (d, *J* = 6.1 Hz, 3H), 0.94 (d, *J* = 6.2 Hz, 3H).

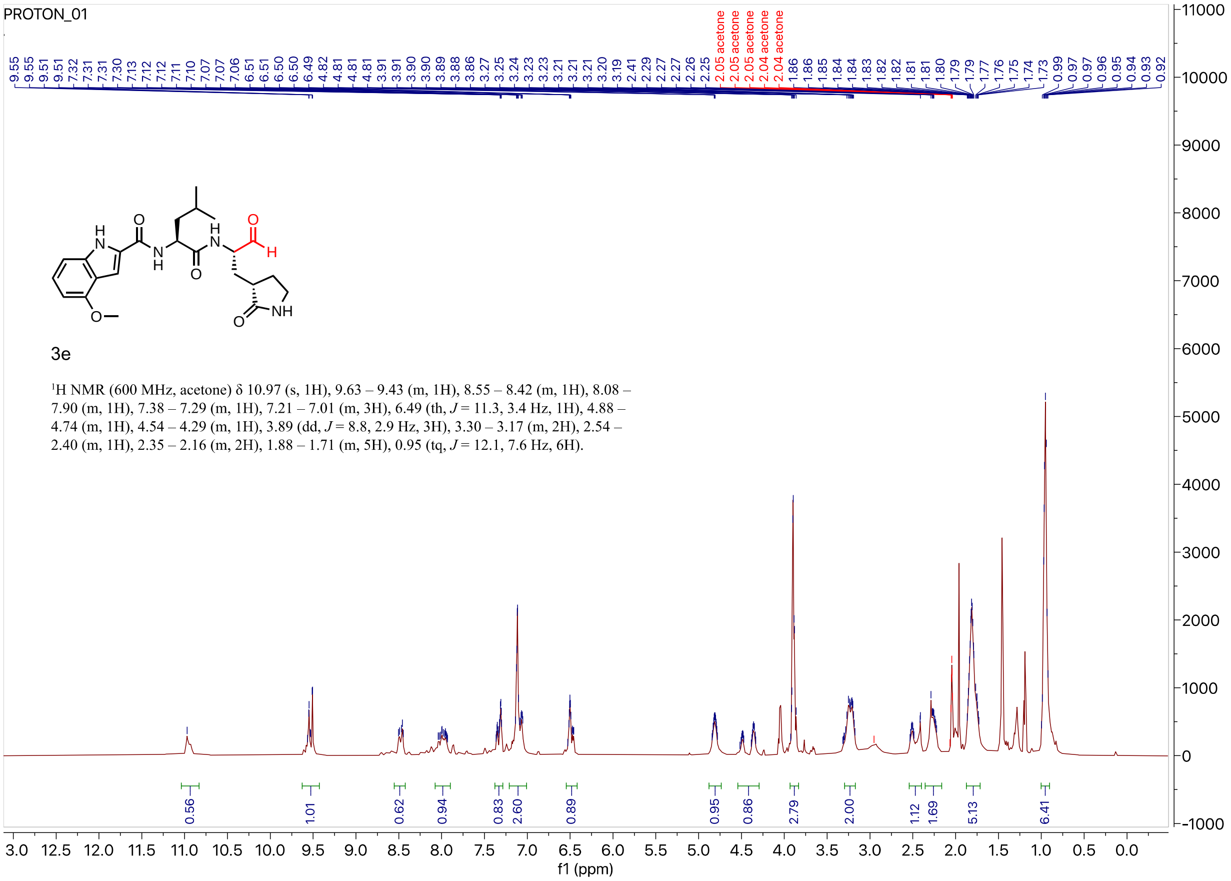

3e

^{1}H NMR (600 MHz, acetone) δ 10.97 (s, 1H), 9.63 – 9.43 (m, 1H), 8.55 – 8.42 (m, 1H), 8.08 – 7.90 (m, 1H), 7.38 – 7.29 (m, 1H), 7.21 – 7.01 (m, 3H), 6.49 (th, *J* = 11.3, 3.4 Hz, 1H), 4.88 – 4.74 (m, 1H), 4.54 – 4.29 (m, 1H), 3.89 (dd, *J* = 8.8, 2.9 Hz, 3H), 3.30 – 3.17 (m, 2H), 2.54 – 2.40 (m, 1H), 2.35 – 2.16 (m, 2H), 1.88 – 1.71 (m, 5H), 0.95 (tq, *J* = 12.1, 7.6 Hz, 6H).

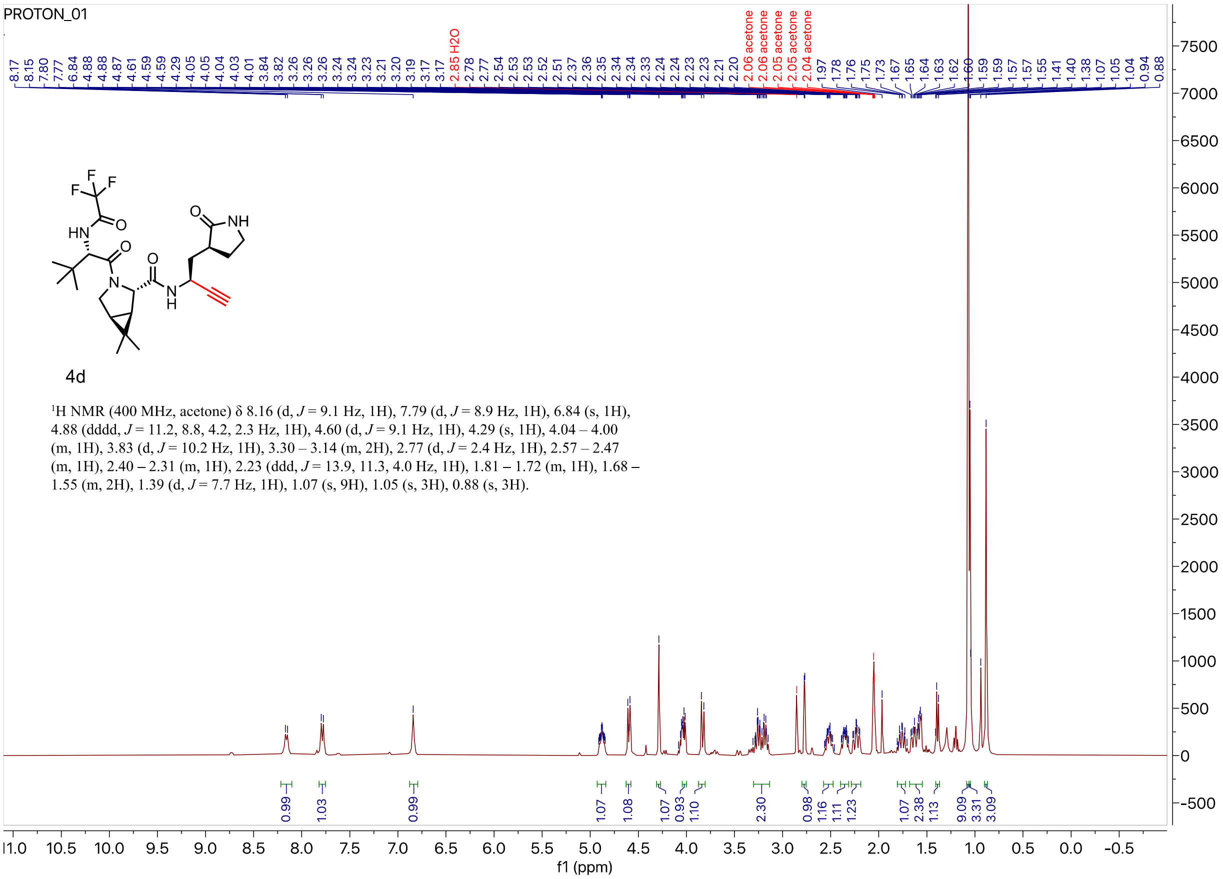

4d

^{1}H NMR (400 MHz, acetone) δ 8.16 (d, *J* = 9.1 Hz, 1H), 7.79 (d, *J* = 8.9 Hz, 1H), 6.84 (s, 1H), 4.88 (dddd, *J* = 11.2, 8.8, 4.2, 2.3 Hz, 1H), 4.60 (d, *J* = 9.1 Hz, 1H), 4.29 (s, 1H), 4.04 – 4.00 (m, 1H), 3.83 (d, *J* = 10.2 Hz, 1H), 3.30 – 3.14 (m, 2H), 2.77 (d, *J* = 2.4 Hz, 1H), 2.57 – 2.47 (m, 1H), 2.40 – 2.31 (m, 1H), 2.23 (ddd, *J* = 13.9, 11.3, 4.0 Hz, 1H), 1.81 – 1.72 (m, 1H), 1.68 – 1.55 (m, 2H), 1.39 (d, *J* = 7.7 Hz, 1H), 1.07 (s, 9H), 1.05 (s, 3H), 0.88 (s, 3H).

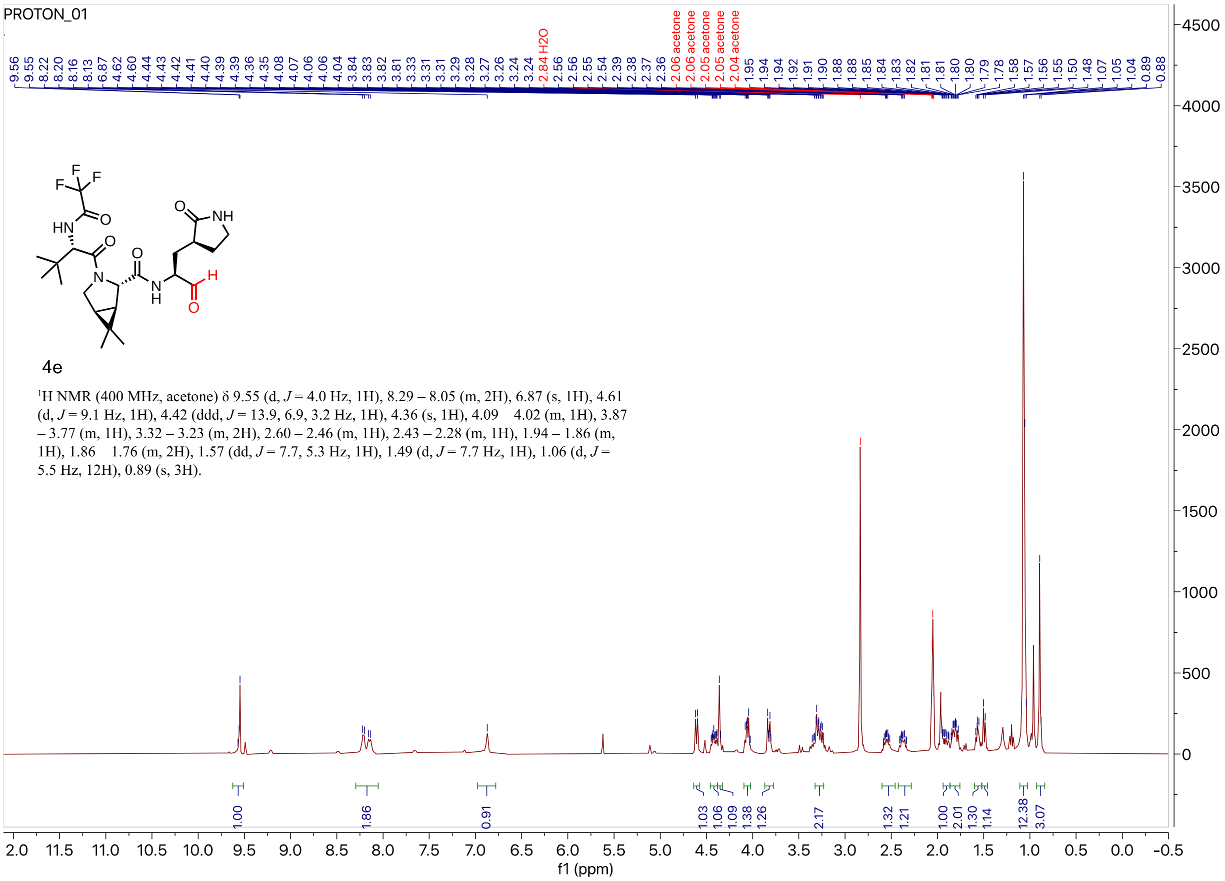

^{1}H NMR (400 MHz, acetone) δ 9.55 (d, J = 4.0 Hz, 1H), 8.29 – 8.05 (m, 2H), 6.87 (s, 1H), 4.61 (d, J = 9.1 Hz, 1H), 4.42 (ddd, J = 13.9, 6.9, 3.2 Hz, 1H), 4.36 (s, 1H), 4.09 – 4.02 (m, 1H), 3.87 – 3.77 (m, 1H), 3.32 – 3.23 (m, 2H), 2.60 – 2.46 (m, 1H), 2.43 – 2.28 (m, 1H), 1.94 – 1.86 (m, 1H), 1.86 – 1.76 (m, 2H), 1.57 (dd, J = 7.7, 5.3 Hz, 1H), 1.49 (d, J = 7.7 Hz, 1H), 1.06 (d, J = 5.5 Hz, 12H), 0.89 (s, 3H).

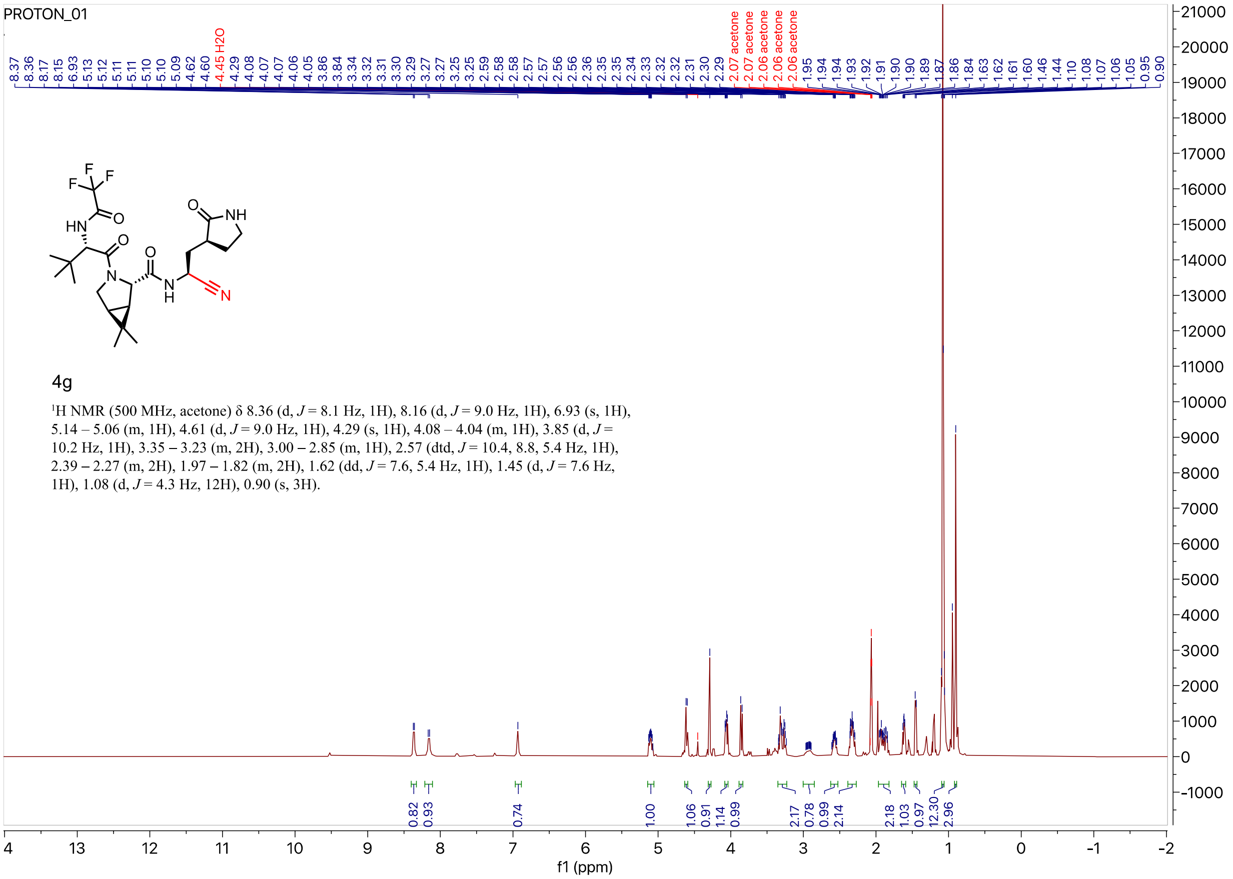
PROTON_01
4g
^{1}H NMR (500 MHz, acetone) δ 8.36 (d, J = 8.1 Hz, 1H), 8.16 (d, J = 9.0 Hz, 1H), 6.93 (s, 1H), 5.14 – 5.06 (m, 1H), 4.61 (d, J = 9.0 Hz, 1H), 4.29 (s, 1H), 4.08 – 4.04 (m, 1H), 3.85 (d, J = 10.2 Hz, 1H), 3.35 – 3.23 (m, 2H), 3.00 – 2.85 (m, 1H), 2.57 (dtd, J = 10.4, 8.8, 5.4 Hz, 1H), 2.39 – 2.27 (m, 2H), 1.97 – 1.82 (m, 2H), 1.62 (dd, J = 7.6, 5.4 Hz, 1H), 1.45 (d, J = 7.6 Hz, 1H), 1.08 (d, J = 4.3 Hz, 12H), 0.90 (s, 3H).
4.45 H2O
2.07 acetone
f1 (ppm)

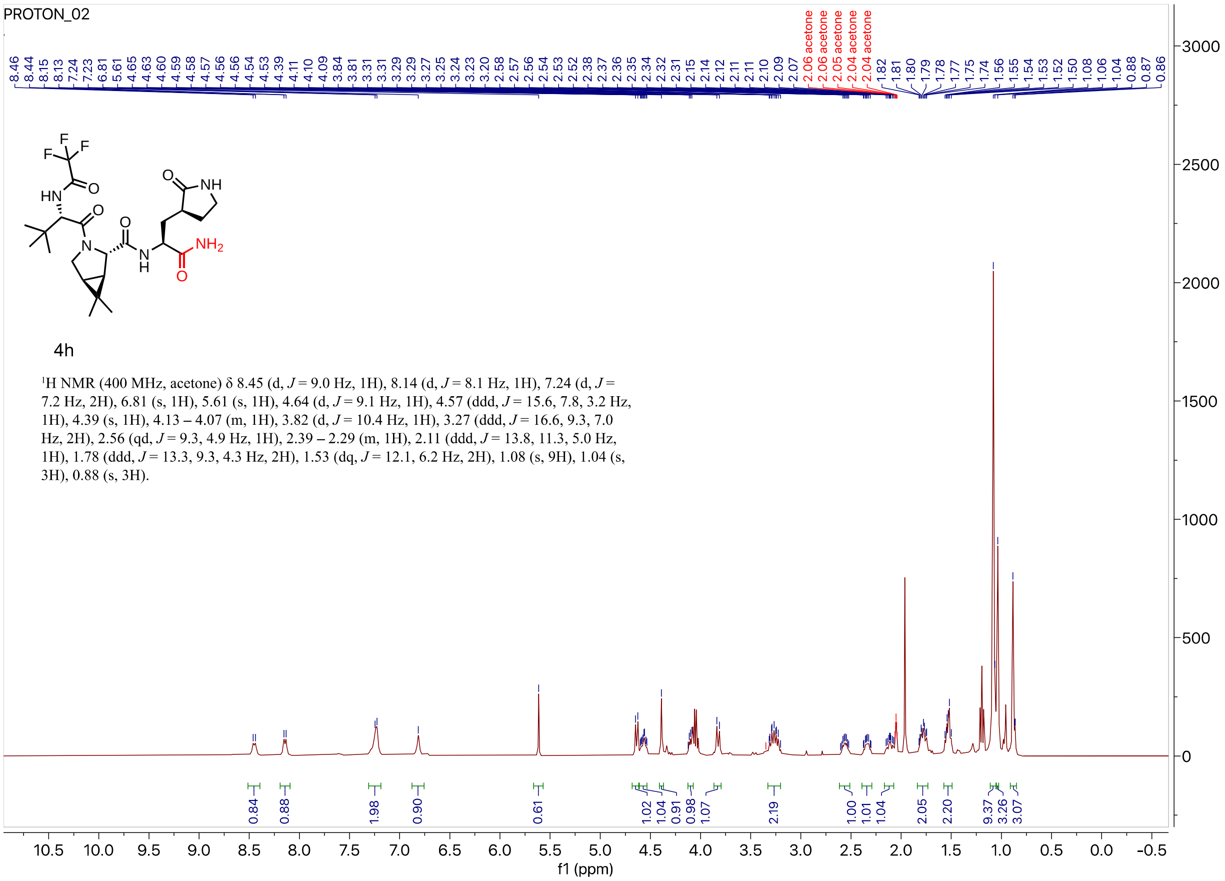

4h

^{1}H NMR (400 MHz, acetone) δ 8.45 (d, *J* = 9.0 Hz, 1H), 8.14 (d, *J* = 8.1 Hz, 1H), 7.24 (d, *J* = 7.2 Hz, 2H), 6.81 (s, 1H), 5.61 (s, 1H), 4.64 (d, *J* = 9.1 Hz, 1H), 4.57 (ddd, *J* = 15.6, 7.8, 3.2 Hz, 1H), 4.39 (s, 1H), 4.13 – 4.07 (m, 1H), 3.82 (d, *J* = 10.4 Hz, 1H), 3.27 (ddd, *J* = 16.6, 9.3, 7.0 Hz, 2H), 2.56 (qd, *J* = 9.3, 4.9 Hz, 1H), 2.39 – 2.29 (m, 1H), 2.11 (ddd, *J* = 13.8, 11.3, 5.0 Hz, 1H), 1.78 (ddd, *J* = 13.3, 9.3, 4.3 Hz, 2H), 1.53 (dq, *J* = 12.1, 6.2 Hz, 2H), 1.08 (s, 9H), 1.04 (s, 3H), 0.88 (s, 3H).

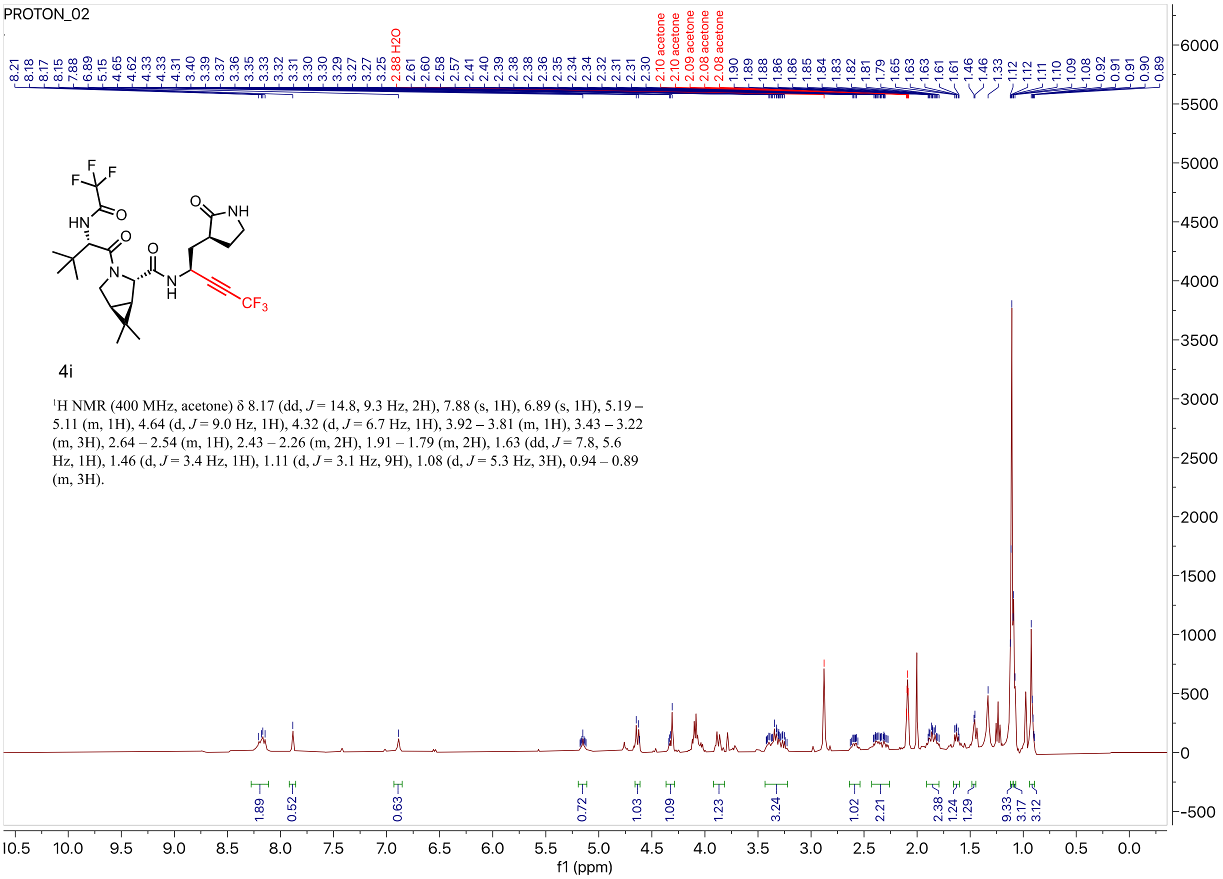

4i

^{1}H NMR (400 MHz, acetone) δ 8.17 (dd, *J* = 14.8, 9.3 Hz, 2H), 7.88 (s, 1H), 6.89 (s, 1H), 5.19 – 5.11 (m, 1H), 4.64 (d, *J* = 9.0 Hz, 1H), 4.32 (d, *J* = 6.7 Hz, 1H), 3.92 – 3.81 (m, 1H), 3.43 – 3.22 (m, 3H), 2.64 – 2.54 (m, 1H), 2.43 – 2.26 (m, 2H), 1.91 – 1.79 (m, 2H), 1.63 (dd, *J* = 7.8, 5.6 Hz, 1H), 1.46 (d, *J* = 3.4 Hz, 1H), 1.11 (d, *J* = 3.1 Hz, 9H), 1.08 (d, *J* = 5.3 Hz, 3H), 0.94 – 0.89 (m, 3H).

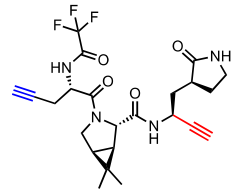

Alk-4d

^{1}H NMR (400 MHz, acetone) δ 8.97 (d, *J* = 8.0 Hz, 1H), 7.80 (d, *J* = 8.6 Hz, 1H), 7.08 (s, 1H), 4.92 – 4.83 (m, 2H), 4.32 (s, 1H), 4.12 – 4.05 (m, 1H), 3.91 (d, *J* = 10.3 Hz, 1H), 3.30 (dtd, *J* = 16.0, 8.6, 4.1 Hz, 2H), 2.82 – 2.71 (m, 3H), 2.60 (t, *J* = 2.6 Hz, 1H), 2.52 (ddd, *J* = 12.4, 6.7, 3.4 Hz, 1H), 2.38 (tdd, *J* = 11.1, 7.5, 2.3 Hz, 1H), 2.24 (ddd, *J* = 13.9, 10.7, 4.5 Hz, 1H), 1.84 – 1.76 (m, 1H), 1.70 (ddd, *J* = 14.5, 10.0, 4.9 Hz, 1H), 1.61 (dd, *J* = 7.6, 5.1 Hz, 1H), 1.50 (d, *J* = 7.6 Hz, 1H), 1.10 (s, 3H), 0.97 (s, 3H).

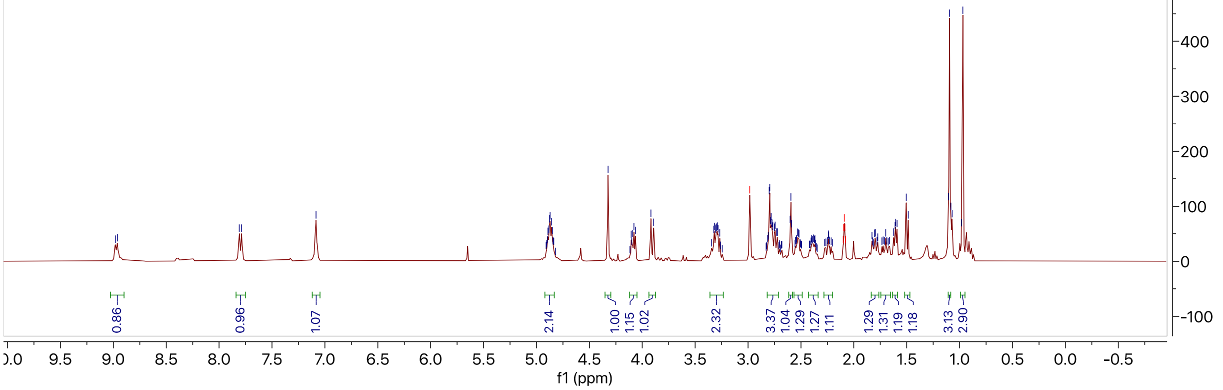

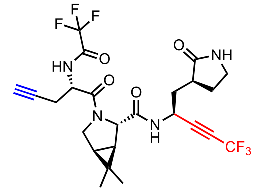

Alk-4i

^{1}H NMR (400 MHz, acetone) δ 9.06 (d, *J* = 6.9 Hz, 1H), 8.66 (d, *J* = 9.3 Hz, 1H), 7.16 (d, *J* = 15.1 Hz, 1H), 4.85 (s, 1H), 4.82 – 4.74 (m, 1H), 4.04 (s, 1H), 3.96 (dd, *J* = 10.5, 5.5 Hz, 1H), 3.65 (d, *J* = 10.6 Hz, 1H), 3.28 (q, *J* = 9.6 Hz, 2H), 2.59 (td, *J* = 6.0, 2.9 Hz, 2H), 2.32 (q, *J* = 3.6 Hz, 2H), 1.85 (t, *J* = 4.6 Hz, 1H), 1.77 (t, *J* = 11.2 Hz, 1H), 1.53 – 1.38 (m, 3H), 1.32 (d, *J* = 7.6 Hz, 1H), 0.92 (s, 3H), 0.77 (s, 3H).

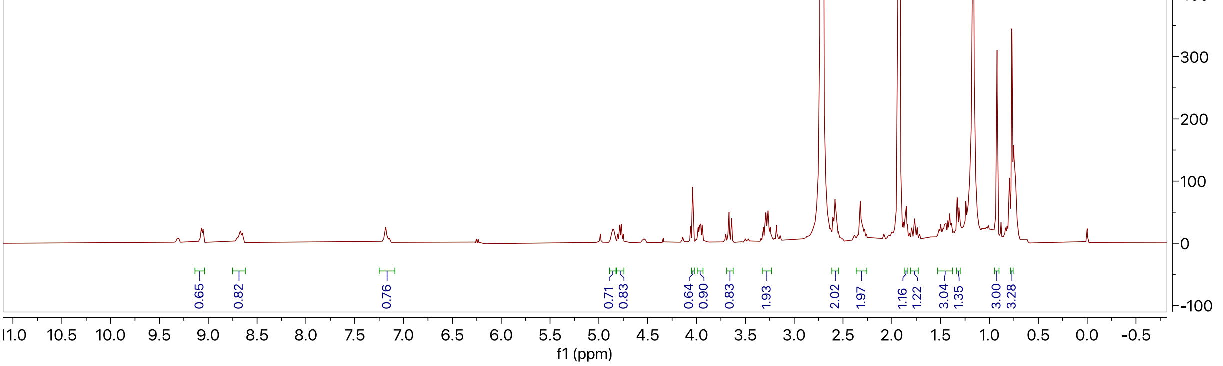

4. Purity Analyses of Representative Compounds via HPLC

4d

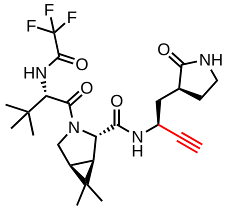

<Sample Information>

Sample Name	: 4d-		
Sample ID	:		
Data Filename	: 4d-1.lcd		
Method Filename	: analytical CN 27 min correct.lcm		
Batch Filename	:		
Vial #	: 1-1	Sample Type	: Unknown
Injection Volume	: 20 uL		
Date Acquired	: 6/29/2023 12:00:35 PM	Acquired by	: System Administrator
Date Processed	: 6/29/2023 5:20:07 PM	Processed by	: System Administrator

<Chromatogram>

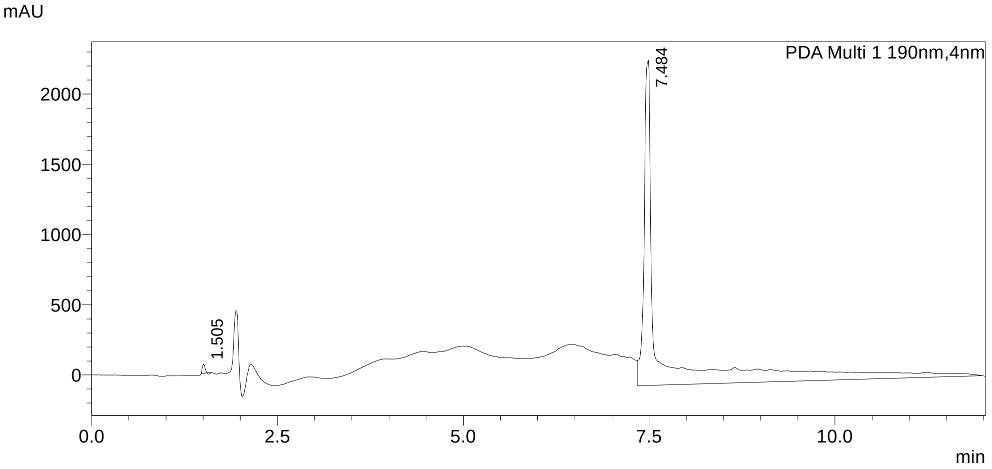

<Peak Table>

PDA Ch1 190nm

Peak#	Ret. Time	Area	Height	Area%
1	1.505	140728	65292	0.447
2	7.484	31355281	2312506	99.553
Total		31496009	2377798	100.000

4g

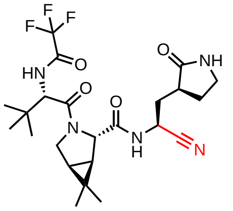

<Sample Information>

Sample Name	: 4g-		
Sample ID	:		
Data Filename	: 4g-1.lcd		
Method Filename	: analytical CN 27 min correct.lcm		
Batch Filename	:		
Vial #	: 1-1	Sample Type	: Unknown
Injection Volume	: 20 uL		
Date Acquired	: 6/29/2023 12:18:24 PM	Acquired by	: System Administrator
Date Processed	: 6/29/2023 5:14:25 PM	Processed by	: System Administrator

<Chromatogram>

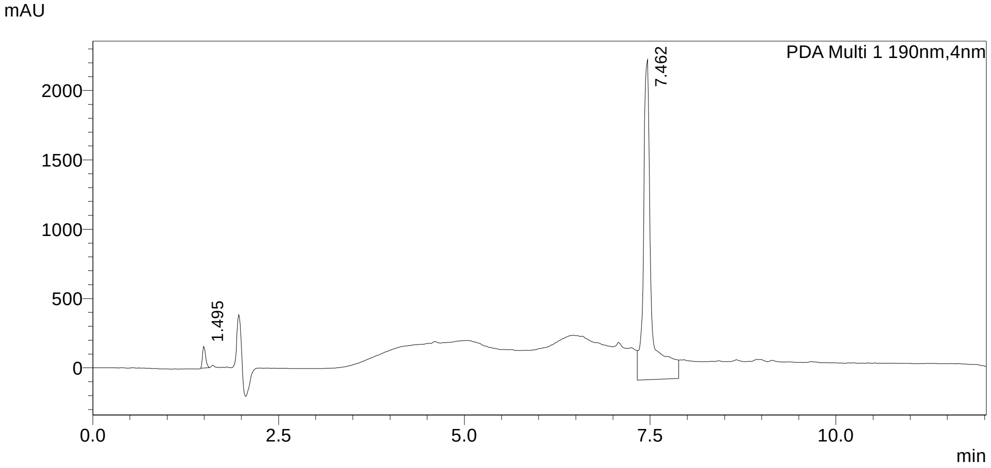

<Peak Table>

PDA Ch1 190nm

Peak#	Ret. Time	Area	Height	Area%
1	1.495	455030	156450	2.653
2	7.462	16698876	2306050	97.347
Total		17153906	2462500	100.000

4i

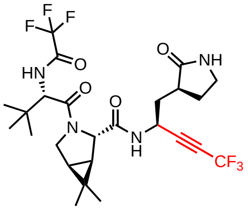

<Sample Information>

Sample Name	: 4i-		
Sample ID	:		
Data Filename	: 4i-6.lcd		
Method Filename	: analytical CN 27 min correct.lcm		
Batch Filename	:		
Vial #	: 1-1	Sample Type	: Unknown
Injection Volume	: 20 uL		
Date Acquired	: 6/29/2023 4:28:43 PM	Acquired by	: System Administrator
Date Processed	: 6/30/2023 7:38:36 PM	Processed by	: System Administrator

<Chromatogram>

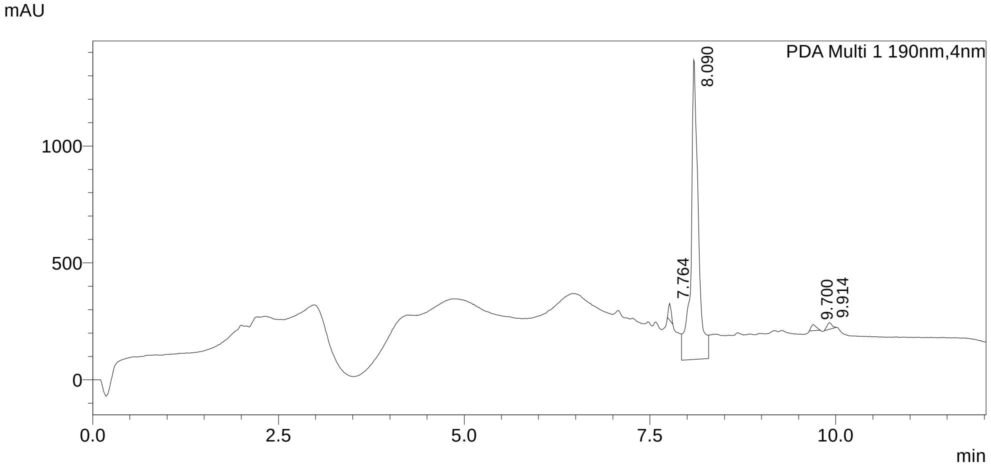

<Peak Table>

Peak Table

PDA Ch1 190nm

Peak#	Ret. Time	Area	Height	Area%
1	7.764	172750	72023	1.913
2	8.090	8597847	1281243	95.235
3	9.700	125843	25760	1.394
4	9.914	131630	27467	1.458
Total		9028070	1406493	100.000

Alk-4d

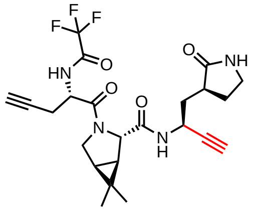

<Sample Information>

Sample Name	: Alk-4d-		
Sample ID	:		
Data Filename	: Alk-4d-8.lcd		
Method Filename	: analytical CN 27 min correct.lcm		
Batch Filename	:		
Vial #	: 1-1	Sample Type	: Unknown
Injection Volume	: 20 uL		
Date Acquired	: 6/29/2023 1:52:16 PM	Acquired by	: System Administrator
Date Processed	: 6/30/2023 7:10:09 PM	Processed by	: System Administrator

<Chromatogram>

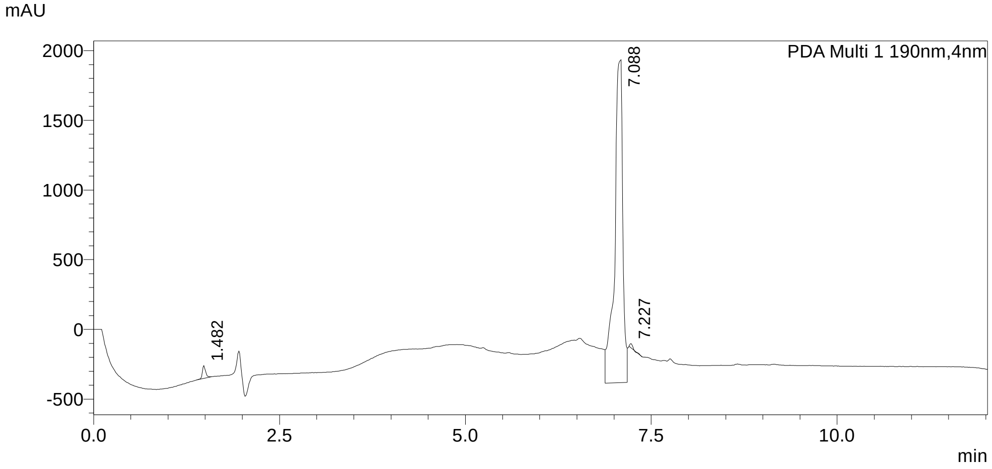

<Peak Table>

Peak Table

PDA Ch1 190nm

Peak#	Ret. Time	Area	Height	Area%
1	1.482	286705	90849	1.607
2	7.088	17511828	2315021	98.145
3	7.227	44220	28529	0.248
Total		17842753	2434399	100.000

Alk-4i

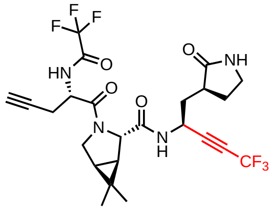

<Sample Information>

Sample Name	: Alk-4d final		
Sample ID	:		
Data Filename	: Alk-4i-5.lcd		
Method Filename	: analytical CN 27 min correct.lcm		
Batch Filename	:		
Vial #	: 1-1	Sample Type	: Unknown
Injection Volume	: 20 uL		
Date Acquired	: 6/29/2023 5:39:11 PM	Acquired by	: System Administrator
Date Processed	: 6/30/2023 6:59:03 PM	Processed by	: System Administrator

<Chromatogram>

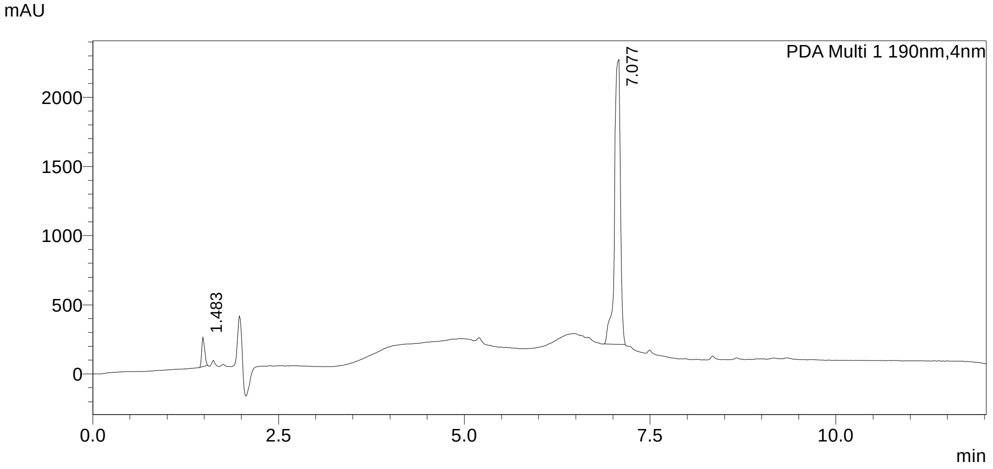

<Peak Table>

Peak Table

PDA Ch1 190nm

Peak#	Ret. Time	Area	Height	Area%
1	1.483	590339	209625	4.973
2	7.077	11280367	2051964	95.027
Total		11870706	2261588	100.000
